# Supplementary material for: MDIC3: Matrix decomposition to infer cell-cell communication
Source: Patterns (N Y). 2024 Jan 11;5(2):100911. doi: 10.1016/j.patter.2023.100911 (PMC10873161; doi:10.1016/j.patter.2023.100911)
Supplement: Document S1. Figures S1–S7, Tables S2–S13, and Notes S1–S8 [file mmc1.pdf]

Patterns, Volume *5*

Supplemental information

MDIC3: Matrix decomposition

to infer cell-cell communication

Yi Liu, Yuelei Zhang, Xiao Chang, and Xiaoping Liu

Supplemental Figures

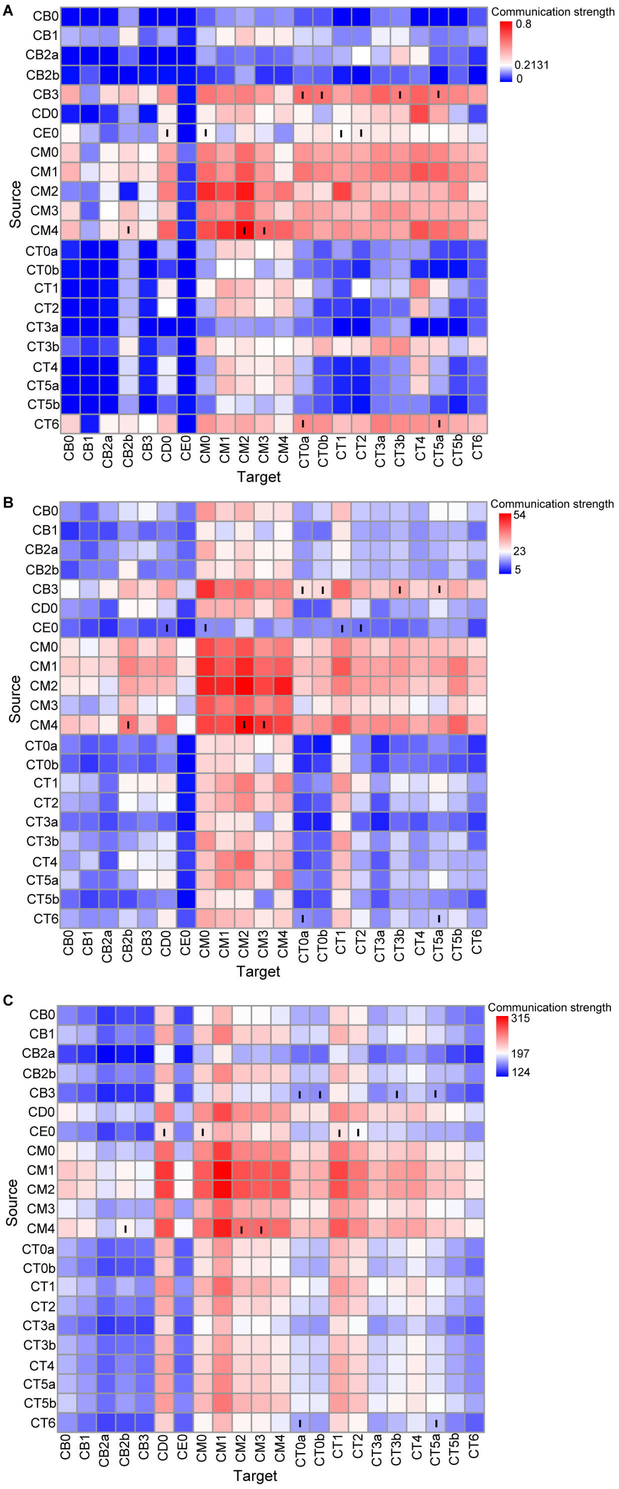

Figure S1. Heatmaps of the inferred intercellular communications on lupus nephritis dataset by existing methods. The heat maps showing the communication among cell types in human lupus nephritis were inferred by **(A)** CellChat, **(B)** Cellphonedb, and **(C)** iTALK. The black "I" indicates interactions mentioned in this main text. The row represents the "source" of a communication, and the column represents the "target" of the communication. For example, the black "I" in row "CB3" and column "CT0a" indicates an intercellular communication from CB3 to CT0a.

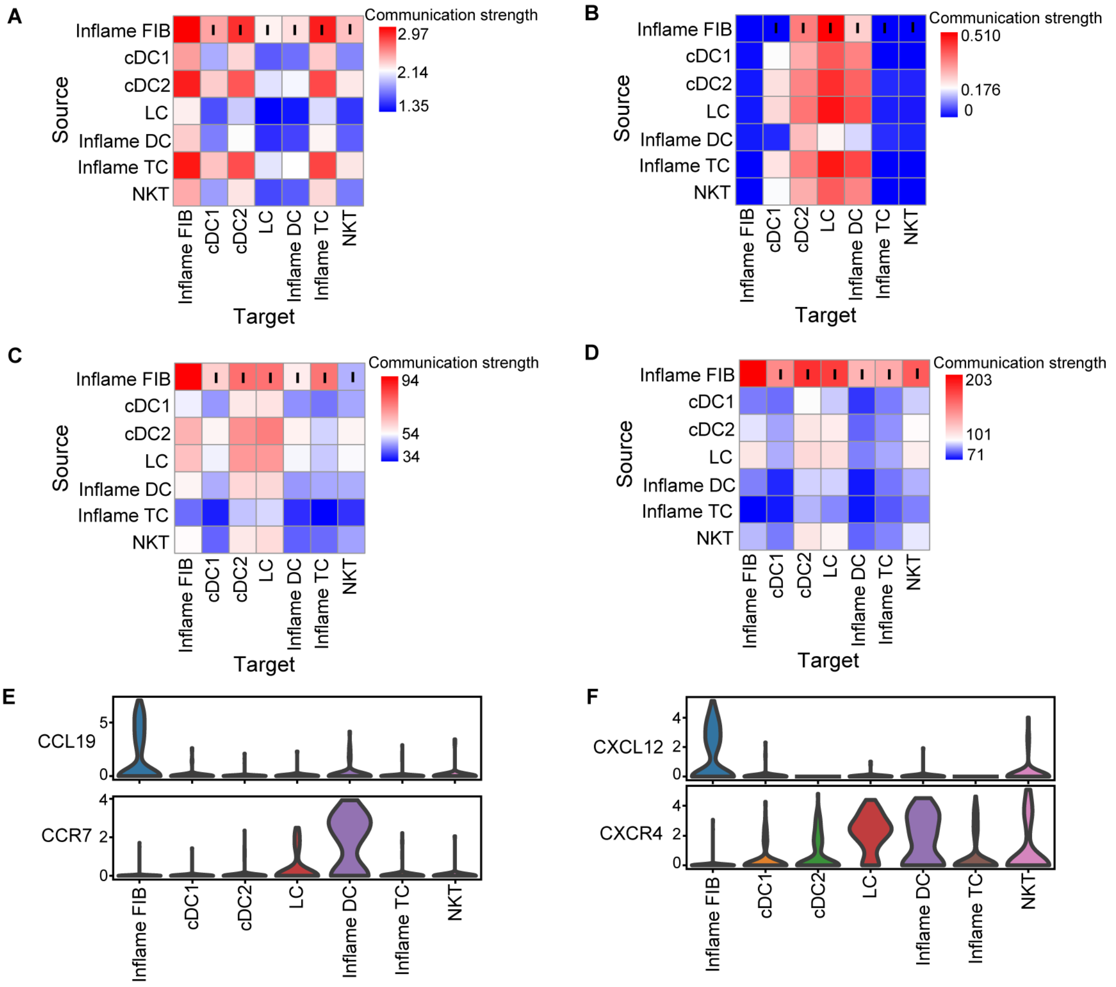

Figure S2. Case study on human lesional skin dataset. The heat maps showing the communication results among cell types in human lesional skin were inferred by **(A)** MDIC3, **(B)** CellChat, **(C)** Cellphonedb, and **(D)** iTALK, respectively. The black "I" indicates interactions mentioned in this main text. The row represents the "source" of a communication, and the column represents the "target" of the communication. For example, the black "I" in row "Inflame FIB" and column "cDC1" means an intercellular communication from Inflame FIB to cDC1. Inflame FIB: inflammatory fibroblasts; cDC: conventional dendritic cell; Inflame DC: inflammatory dendritic cell; LC: Langerhans cell; Inflame TC: inflammatory T cell; NKT: natural killer T cell. **(E)** Violin plot shows the expression of *CCL19-CCR7* signaling in different human lesional skin cell types. **(F)** Violin plot shows the expression of *CXCL12-CXCR4* signaling in different human lesional skin cell types.

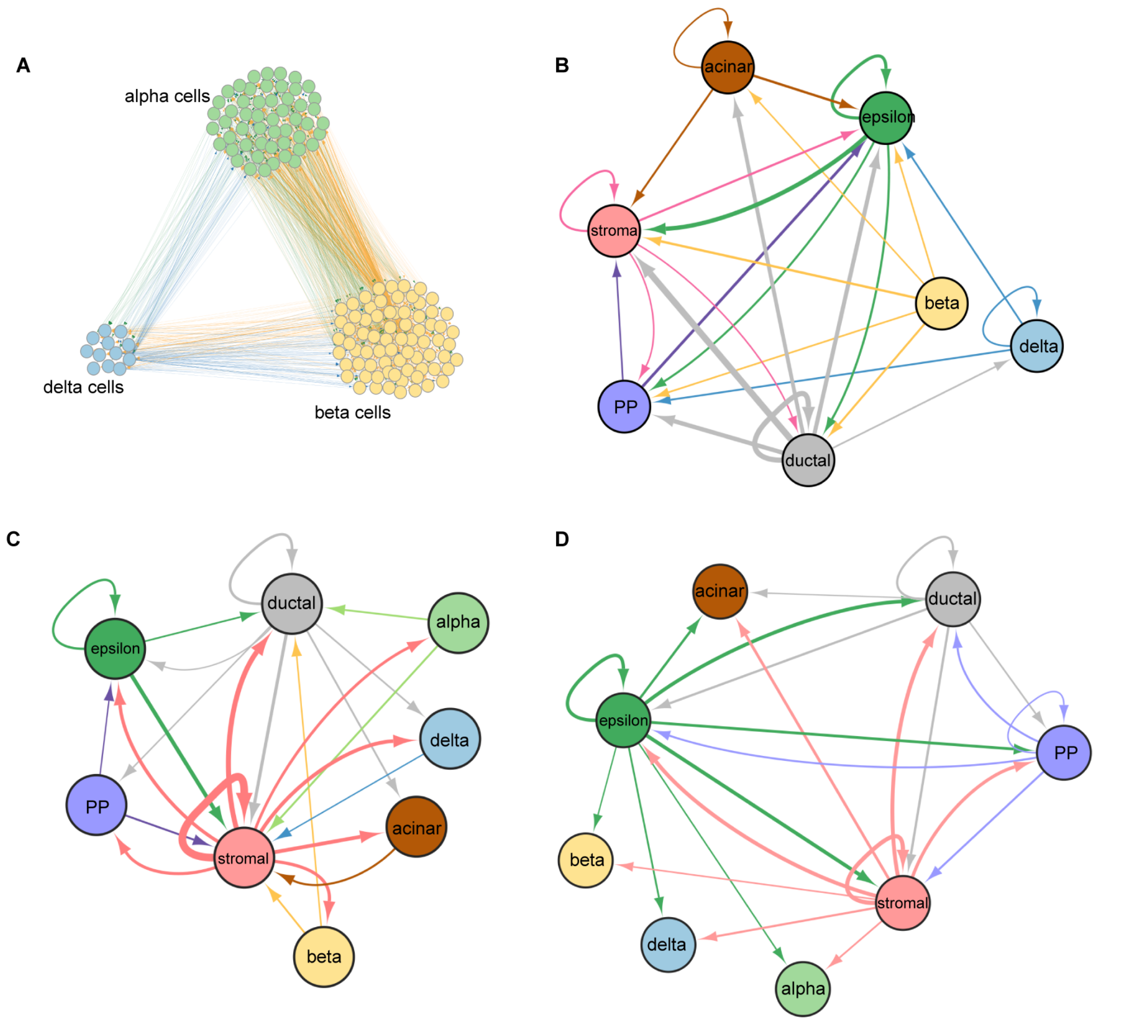

Figure S3. Intercellular communications among human islet cells by MDIC3, CellChat, Cellphonedb, and iTALK. (A) Cell-cell communications among alpha cells, beta cells, and delta cells were inferred by MDIC3. The line with different color indicates that the communication signal is sent from the cell with the same color as the line. The figure shows paracrine interactions among the three cell types. The communication networks among cell types in human islet were inferred by **(B)** CellChat, **(C)** Cellphonedb, and **(D)** iTALK, respectively. The arrow indicates the direction of signal transference, and the edge width represents the communication strength.

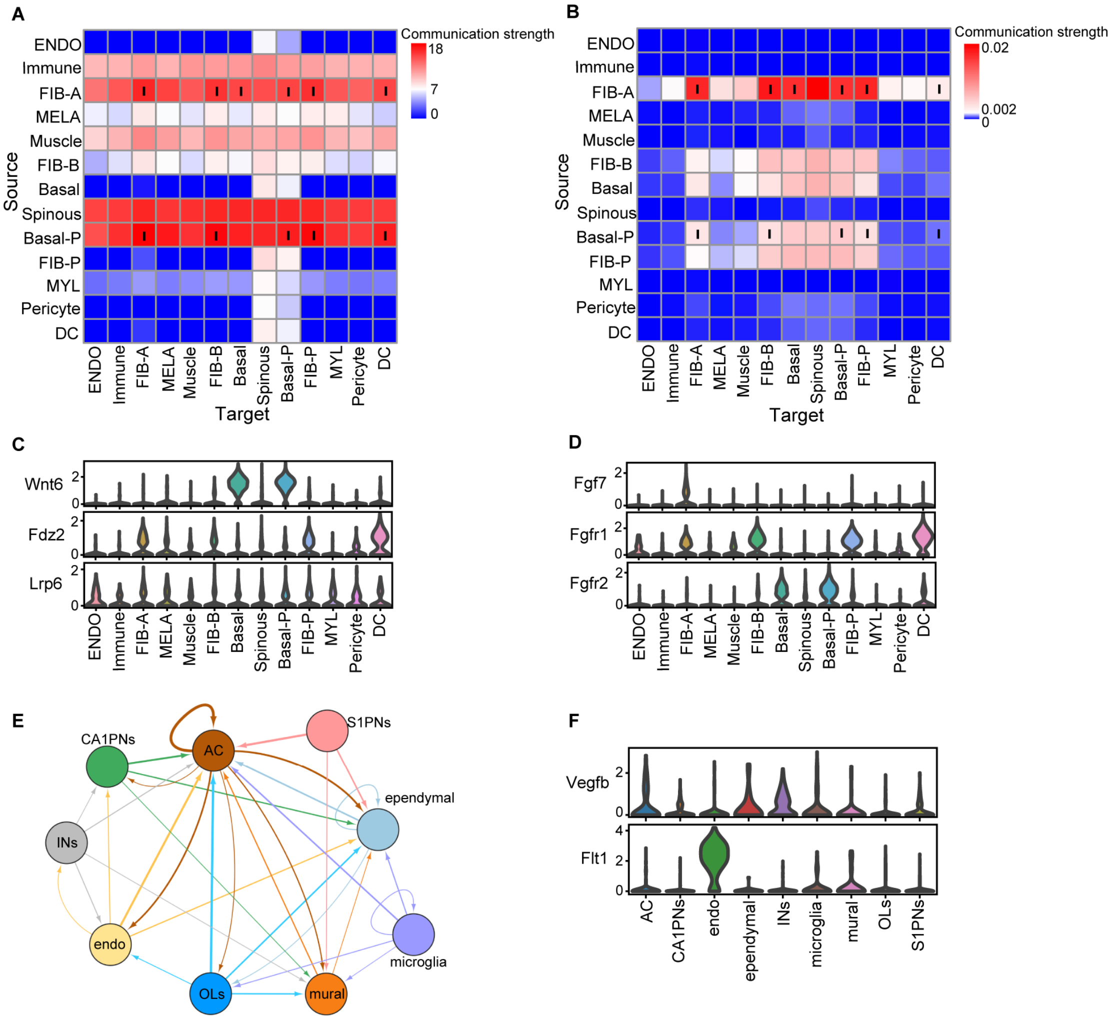

Figure S4. Case study on mouse species datasets. (A) and **(B)** are heatmaps showing the communication results among cell types in E14.5 mouse skin inferred by MDIC3 and CellChat, respectively. Black "I" indicates interactions mentioned in this main text. The row represents the "source" of a communication, and the column represents the "target" of the communication. For example, the black "I" in row "FIB-A" and column "FIB-B" means an intercellular communication from FIB-A to FIB-B. **(C)** Violin plot shows the expression of *Wnt6 - (Fzd2+Lrp6)* gene pairs under the WNT signaling in E14.5 mouse skin cell types. **(D)** Violin plot shows the expression *Fgf7-Fgfr1* and *Fgf7-Fgfr2* gene pairs under the FGF signaling in E14.5 mouse skin cell types. ENDO: endothelial cells; Immune: immune cells; FIB-A: fibroblast type A; MELA: melanocyte; Muscle: muscle cells; FIB-B: fibroblast type B; Basal: basal cells; Spinous: spinous epithelial cells; Basal-P: proliferative basal cells; FIB-P: proliferative fibroblasts; MYL: myeloid cells; DC: Dendritic cells. **(E)** The communication network among cell types in mouse brain was inferred by CellChat. The arrow indicates the direction of signal transference. The edge width represents the communication strength. S1PNs: S1 pyramidal neurons; CA1PNs: CA1 pyramidal neurons; INs: interneurons; OLs: oligodendrocytes; AC: astrocytes; endo: vascular endothelial cells. **(F)** Violin plot shows the expression of *Vegfb-Flt1* L-R signaling for different mouse brain cell types.

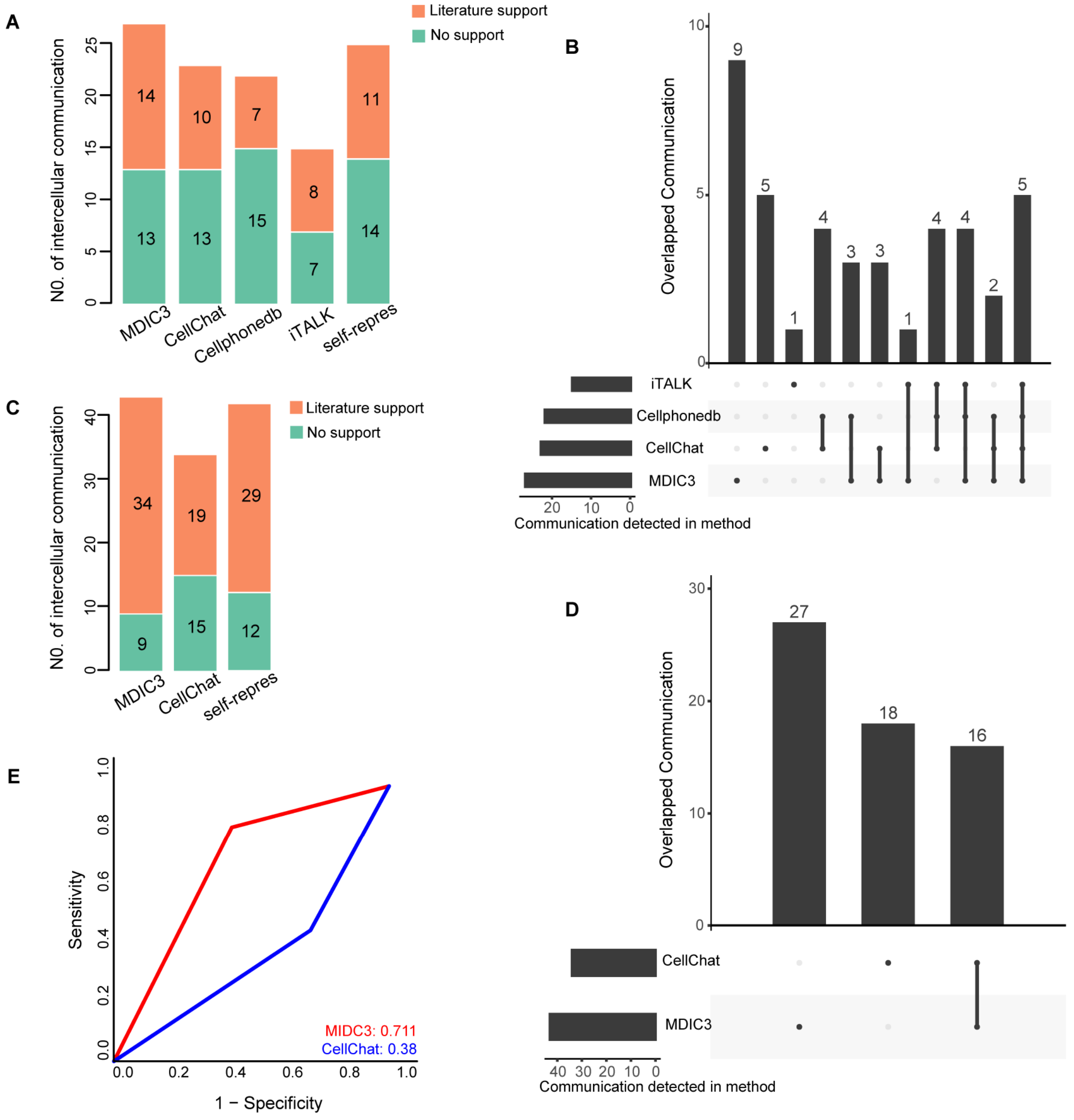

Figure S5. Comparison of the predictive performance of MDIC3 with other methods using scRNA-seq. (A) Comparison of literature support (Table S3) for the intercellular communications by MDIC3 and four other tools (CellChat, Cellphonedb, iTALK, and the two-side self-representation model) on human lesional skin dataset. The horizontal ordinate represents the five methods, and the vertical ordinate is the number of intercellular communications from the five methods. The orange bar represents the number of intercellular communications with literature support, and the green bar represents the number of intercellular communications without literature support from PubMed database (https://pubmed.ncbi.nlm.nih.gov/). **(B)** UpSetR plot of intercellular communications on human lesional skin dataset from four tools (MDIC3, CellChat, Cellphonedb, and iTALK). The horizontal bar graph at the bottom left represents the total number of cell-cell communications detected by different methods. Use a dot to refer to the corresponding method on the left. If cell-cell communications were only detected by one method, only one gray dot is darkened, and the number of the detected cell-cell communications are shown in bar graph form above. The intersection of the cell-cell communication results inferred by

different methods are shown by multiple black dots and a line connecting them, and the number of the intersection of intercellular communication results is represented by the bar graph form above. **(C)** Comparison of literature support (Table S4) for intercellular communications by MDIC3, CellChat and the two-side self-representation model on mouse brain dataset. **(D)** UpSetR plot of intercellular communications on mouse brain dataset from MDIC3 and CellChat. **(E)** ROC curves of MDIC3 and CellChat for literature support (Table S4) on mouse brain dataset.

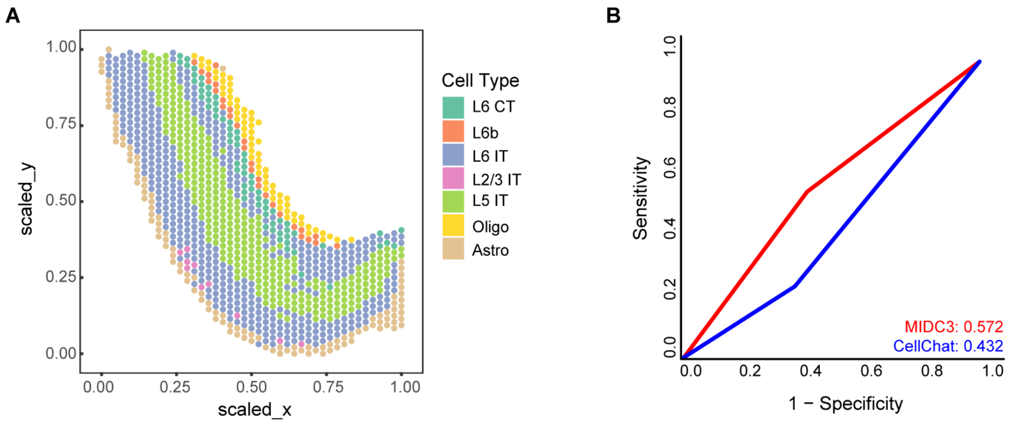

Figure S6. Comparison of the predictive performance of MDIC3 with CellChat using spatial dataset. (A) Spatial plot with pie chart shows the spatial location of each spot. The spot labels were selected by the cell type with the largest proportion of each spot. **(B)** ROC curves of MDIC3 and CellChat for literature support (Table S5) on mouse brain 10X visium spatial dataset.

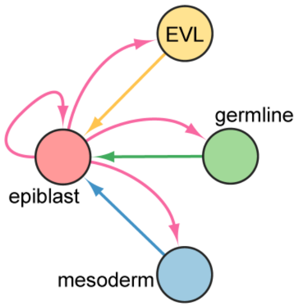

Figure S7. The communication networks among cell types in 4hpf zebrafish embryo inferred by MDIC3

Supplemental Tables

Table S2. Literature curated of the inferred cellular communications between human islet endocrine cells

Cellular Communication	MDIC3	CellChat	Cellphonedb	iTALK	Self-representation	PMID
alpha-alpha	1	0	0	0	1	19817798
alpha-beta	1	0	0	0	1	23022232;19817798
alpha-delta	1	0	0	0	1	32067063;19602585;682984;27390011
alpha-epsilon	0	0	0	0	0	no
alpha-pp	0	0	0	0	1	no
beta-alpha	1	0	0	0	1	23022232;19817798
beta-beta	1	0	0	0	1	26384384;19817798
beta-delta	1	0	0	0	1	no
beta-epsilon	0	1	0	0	0	no
beta-pp	1	1	0	0	1	no
delta-alpha	1	0	0	0	1	32067063
delta-beta	1	0	0	0	1	32067063
delta-delta	0	1	0	0	1	32067063;27390011;27408771
delta-epsilon	0	1	0	0	0	no
delta-pp	0	1	0	0	0	no
epsilon-alpha	0	0	0	1	0	19817798;27408771
epsilon-beta	1	0	0	1	0	19817798;27408771
epsilon-delta	0	0	0	1	0	32067063; 27390011
epsilon-epsilon	0	1	1	1	0	no
epsilon-pp	0	1	0	1	0	no
pp-alpha	1	0	0	0	1	32067063;25445712
pp-beta	1	0	0	0	1	32067063;28069397

pp-delta	0	0	0	0	0	no
pp-epsilon	0	1	1	1	0	no
pp-pp	0	0	0	1	0	no

Table S3. Literature curated of the inferred cellular communications between human lesional skin cells

Cellular Communication	MDIC3	CellChat	Cellphonedb	iTALK	Self-representation	PMID
Inflame FIB-Inflame FIB	1	0	1	1	1	no
Inflame FIB-cDC1	1	0	1	1	1	20538798
Inflame FIB-cDC2	1	1	1	1	1	20538798
Inflame FIB-LC	1	1	1	1	1	20538798
Inflame FIB-Inflame DC	1	1	1	1	1	20538798；32035984
Inflame FIB-Inflame TC	1	0	1	1	1	32035984；25182982
Inflame FIB-NKT	1	0	0	1	1	20538798；33516870
cDC1-Inflame FIB	1	0	0	0	1	no
cDC1-cDC2	1	1	1	0	1	no
cDC1-LC	0	1	1	0	0	no
cDC1-Inflame DC	0	1	0	0	0	no
cDC1-Inflame TC	1	0	0	0	1	20538798；15813816；15113590；11876749；19667061
cDC2-Inflame FIB	1	0	1	0	1	no
cDC2-cDC1	1	1	1	0	1	no
cDC2-cDC2	1	1	1	1	1	no
cDC2-LC	0	1	1	1	1	no
cDC2-Inflame DC	0	1	1	0	1	no
cDC2-Inflame TC	1	0	0	0	1	20538798；15813816；15113590；11876749；19667061
cDC2-NKT	1	0	1	0	1	no
LC-Inflame FIB	1	0	1	1	1	no
LC-cDC1	0	1	0	0	0	no
LC-cDC2	0	1	1	1	0	no

LC-LC	0	1	1	1	0	no
LC-Inflame DC	0	1	0	0	0	no
LC-NKT	0	0	0	1	0	no
Inflame DC-Inflame FIB	1	0	1	0	1	no
Inflame DC-cDC2	0	1	1	0	1	no
Inflame DC-LC	0	1	1	0	0	no
Inflame DC-Inflame TC	1	0	0	0	0	20538798；15813816；15113590；11876749；19667061
Inflame TC-Inflame FIB	1	0	0	0	1	no
Inflame TC-cDC1	1	1	0	0	1	10808183
Inflame TC-cDC2	1	1	0	0	1	10808183
Inflame TC-LC	0	1	0	0	0	10808183
Inflame TC-Inflame DC	1	1	0	0	0	10808183
Inflame TC-Inflame TC	1	0	0	0	1	16815153；10808183；15813816
Inflame TC-NKT	1	0	0	0	0	no
NKT-Inflame FIB	1	0	0	0	1	no
NKT-cDC2	1	1	1	1	0	16978532
NKT-LC	0	1	1	1	0	16978532
NKT-Inflame DC	0	1	0	0	0	16978532
NKT-Inflame TC	1	0	0	0	0	no

Table S4. Literature curated of the inferred cellular communications between mouse brain cells

Cellular Communication	MDIC3	CellChat	Self-representation	PMID
astrocytes-astrocytes	0	1	0	21119776;19243701
astrocytes-ependymal	0	1	0	no
astrocytes-oligodendrocytes	1	1	1	21119776;12966207
astrocytes-ca1pyramidal	1	1	1	24204341
astrocytes-s1pyramidal	1	0	1	16212146
astrocytes-endothelial	0	1	1	19243701
astrocytes-mural	0	1	0	27775719
microglia-astrocytes	1	1	0	35201268;32765501
microglia-microglia	1	1	0	25171395
microglia-ependymal	0	1	0	no
microglia-oligodendrocytes	1	1	1	23981039
microglia-ca1pyramidal	1	0	1	30319362；23650361；31677479
microglia-s1pyramidal	1	0	0	30319362；23650361；31677479
microglia-interneurons	1	0	0	30319362；23650361；31677479
microglia-endothelial	1	0	0	35201268
microglia-mural	1	1	0	23416119
ependymal-astrocytes	0	1	0	21119776
ependymal-ependymal	0	1	0	no
ependymal-oligodendrocytes	0	1	0	21119776;12966207
oligodendrocytes-astrocytes	1	1	1	23717302
oligodendrocytes-microglia	1	0	1	23981039
oligodendrocytes-ependymal	0	1	0	no
oligodendrocytes-oligodendrocytes	1	0	1	33208471
oligodendrocytes-ca1pyramidal	1	0	1	31410988

oligodendrocytes-s1pyramidal	1	0	1	31410988
oligodendrocytes-interneurons	1	0	1	31410988
oligodendrocytes-endothelial	1	1	1	33969732
oligodendrocytes-mural	0	1	1	no
ca1pyramidal-astrocytes	1	1	1	27775719
ca1pyramidal-microglia	0	0	1	31729301
ca1pyramidal-ependymal	0	1	0	no
ca1pyramidal-oligodendrocytes	1	0	1	23717302
ca1pyramidal-ca1pyramidal	1	0	1	28069946；29547843
ca1pyramidal-s1pyramidal	1	0	1	no
ca1pyramidal-interneurons	1	0	1	no
ca1pyramidal-endothelial	1	0	1	31677479;14550787;28535372;25535736
ca1pyramidal-mural	0	1	1	no
s1pyramidal-astrocytes	1	1	1	27775719
s1pyramidal-ependymal	0	1	1	no
s1pyramidal-oligodendrocytes	1	0	0	23717302
s1pyramidal-ca1pyramidal	1	0	1	no
s1pyramidal-s1pyramidal	1	0	1	20369290
s1pyramidal-interneurons	1	0	1	20369290
s1pyramidal-endothelial	1	0	1	31677479;14550787;28535372;25535736
s1pyramidal-mural	0	1	1	no
s1pyramidal-microglia	0	0	1	31729301
interneurons-astrocytes	1	1	1	27775719
interneurons-microglia	1	0	0	31729301;25535736
interneurons-ependymal	1	0	0	no
interneurons-oligodendrocytes	1	0	1	23717302

interneurons-ca1pyramidal	1	1	1	no
interneurons-s1pyramidal	1	0	1	no
interneurons-interneurons	1	0	1	11777931
interneurons-endothelial	1	1	1	31677479;14550787;28535372;25535736
interneurons-mural	1	1	0	no
endothelial-astrocytes	1	1	1	15283992
endothelial-ependymal	0	1	0	no
endothelial-oligodendrocytes	1	0	1	31410988
endothelial-ca1pyramidal	1	1	1	no
endothelial-s1pyramidal	1	0	1	no
endothelial-interneurons	0	1	1	no
mural-astrocytes	0	1	0	30894190;23982198
mural-ependymal	0	1	0	no

Table S5. Results of MDIC3 and CellChat applied on mouse brain 10X visium spatial dataset

Cellular Communication	MDIC3	CellChat	label
Astro-Astro	1	1	1
Astro-L2/3 IT	0	0	1
Astro-L5 IT	1	0	0
Astro-L6 CT	1	0	0
Astro-L6 IT	1	0	1
Astro-L6b	0	0	0
Astro-Oligo	0	1	0
L2/3 IT-Astro	0	1	1
L2/3 IT-L2/3 IT	0	0	1
L2/3 IT-L5 IT	0	0	0
L2/3 IT-L6 CT	0	0	0
L2/3 IT-L6 IT	0	0	1
L2/3 IT-L6b	0	0	0
L2/3 IT-Oligo	0	1	0
L5 IT-Astro	1	1	0
L5 IT-L2/3 IT	0	0	0
L5 IT-L5 IT	1	0	1
L5 IT-L6 CT	1	0	1
L5 IT-L6 IT	1	1	1
L5 IT-L6b	1	0	0
L5 IT-Oligo	1	1	0
L6 CT-Astro	1	1	0
L6 CT-L2/3 IT	0	0	0
L6 CT-L5 IT	1	0	1

L6 CT-L6 CT	1	0	1
L6 CT-L6 IT	1	0	1
L6 CT-L6b	0	0	1
L6 CT-Oligo	0	1	0
L6 IT-Astro	1	1	1
L6 IT-L2/3 IT	0	0	1
L6 IT-L5 IT	1	0	1
L6 IT-L6 CT	1	0	1
L6 IT-L6 IT	1	0	1
L6 IT-L6b	1	0	1
L6 IT-Oligo	1	1	0
L6b-Astro	0	1	0
L6b-L2/3 IT	0	0	0
L6b-L5 IT	1	0	0
L6b-L6 CT	0	0	1
L6b-L6 IT	1	0	1
L6b-L6b	0	0	1
L6b-Oligo	0	1	1
Oligo-Astro	0	1	0
Oligo-L2/3 IT	0	0	0
Oligo-L5 IT	1	0	0
Oligo-L6 CT	0	0	0
Oligo-L6 IT	1	0	0
Oligo-L6b	0	0	1
Oligo-Oligo	0	1	1

Table S6. L-R pairs involved in the communication between inflame FIB and inflame DC by using MDIC3 and CellChat

	**MDIC3**	**CellChat**
Inflame FIB-Inflame DC	*CCL19-CCR7*<br>*CXCL12-CXCR4*	*CCL19-CCR7*<br>*CXCL12-CXCR4*<br>*MIF-(CD74+CXCR4)*

Table S7. L-R pairs involved in the communication between Basal-P and FIB-B by using MDIC3 and CellChat

	MDIC3	CellChat
(Basal-P)-(FIB-B)	*Pdgfa-Pdgfra*	*Pdgfa-Pdgfra*
	Pdgfc-Pdgfra	*Pdgfc-Pdgfra*
	Ngf-Ngfr	*Ngf-Ngfr*
	Pdgfa-Pdgfrb	*Pdgfa-Pdgfrb*
	Wnt10a-(Fzd2+Lrp5)	*Mdk-Sdc2*
	Wnt10a-(Fzd2+Lrp6)	*Mdk-Lrp1*
	Wnt16-(Fzd2+Lrp5)	*Ptn-Sdc2*
	Wnt16-(Fzd2+Lrp6)	*Ptn-Ncl*
	Wnt4-(Fzd2+Lrp5)	
	Wnt4-(Fzd2+Lrp6)	
	Wnt3-(Fzd2+Lrp5)	
	Wnt3 -(Fzd2+Lrp6)	
	Wnt6-(Fzd2+Lrp5)	
	Wnt6-(Fzd2+Lrp6)	
	Wnt7b-(Fzd2+Lrp5)	
	Wnt7b-(Fzd2+Lrp6)	
	Mpzl1-Mpzl1	

Table S8. L-R pairs involved in autocrine communication of Basal by using MDIC3 and CellChat

	MDIC3	CellChat
	Wnt10a-(Fzd6+Lrp5)	*Pdgfa-Pdgfrb*
	Wnt10a-(Fzd6+Lrp6)	*Mdk-Sdc1*
	Wnt16-(Fzd6+Lrp5)	*Mdk-Sdc4*
	Wnt16-(Fzd6+Lrp6)	*Mdk-(ITGA6+ITGB1)*
	Wnt3-(Fzd10+Lrp5)	*Mdk-Ncl*
	Wnt3-(Fzd10+Lrp6)	*Ptn-Sdc1*
	Wnt4-(Fzd10+Lrp5)	*Ptn-Sdc2*
	Wnt4-(Fzd10+Lrp6)	*Ptn-Sdc4*
	Wnt7b-(Fzd10+Lrp5)	*Ptn-Nc1*
	Wnt7b-(Fzd10+Lrp6)	
	Wnt10a-(Fzd10+Lrp5)	
Basal-Basal	*Wnt10a-(Fzd10+Lrp6)*	
	Wnt7b-(Fzd6+Lrp5)	
	Wnt7b-(Fzd6+Lrp6)	
	Wnt6-(Fzd10+Lrp6)	
	Wnt3-(Fzd6+Lrp5)	
	Wnt3-(Fzd6+Lrp6)	
	Wnt16-(Fzd10+Lrp5)	
	Wnt16-(Fzd10+Lrp6)	
	Wnt4-(Fzd6+Lrp5)	
	Wnt4-(Fzd6+Lrp6)	
	Wnt6-(Fzd6+Lrp5)	
	Wnt6-(Fzd6+Lrp6)	

Wnt6-(Fzd10+Lrp5)
Cdh1-Cdh1
Cadm1-Cadm1
Bmp7-(Bmpr1a+Acvr2a)
Bmp7-(Bmpr1a+Acvr2b)
Bmp7-(Bmpr1a+Bmpr2)
Agrn-Dag1
Lamb1-Dag1
Lamb1-(Itga6+Itgb4)
Col4a1-Sdc4
Col4a2-Sdc4

Table S9. Comparison of the feature of MDIC3 with other tools

		MDIC3	CellChat	Cellphonedb	iTALK
**Input**	Need for L-R database	N	Y	Y	Y
	Species restriction	any species	human and mouse	human	human
**Approach**	Basis of inference level	single cells	cell groups	cell groups	Cell groups
	Inference score	global	L-R or pathway	L-R	L-R
**Output**	Predict global communication	Y	N	N	N

Table S10. Literature support results under different gene coverage of datasets

	Literature support	No support
The original human islet dataset matrix (60.96%)	10	2
Matrix1 (70.01%)	10	3
Matrix2 (80.08%)	10	3
Matrix3 (91.25%)	9	6

Table S11. Computational cost of MDIC3 in simulation datasets

	Running time	Peak memory
gene number=20000, cell number=100	0.66min	7851.54 MiB
gene number=20000, cell number=500	1.02min	8066.65 MiB
gene number=20000, cell number=1000	1.20min	8334.51 MiB
gene number=20000, cell number=1500	1.74min	8602.74 MiB
gene number=20000, cell number=2000	2.04min	8871.08 MiB
gene number=20000, cell number=2500	2.46min	9139.76 MiB
gene number=20000, cell number=3000	2.70min	9408.53 MiB
gene number=20000, cell number=3500	3.06min	9676.65 MiB
gene number=20000, cell number=4000	4.15min	9943.76 MiB
gene number=20000, cell number=4500	4.42min	10210.82 MiB
gene number=20000, cell number=5000	4.95min	10477.86 MiB

Table S12. Computational cost of MDIC3 in real datasets

	Human lesional skin dataset	E14.5 mouse skin dataset	Mouse brain dataset
Data information	1394cells<br>11064genes	13179cells<br>14077genes	3005cells<br>19972gene
Running time	1.6min	16.067min	5.683min
Memory increments	9398.27 MiB	23059.16 MiB	23093.74 MiB

Table S13. Literature support results for communication results using different GRN tools

	Human lesional skin cell dataset		Human islet endocrine cell dataset	
	Literature support	No support	Literature support	No support
GNIPLR	14	13	10	2
GENIE3	13	13	10	2
GRNBoost	13	14	5	1

Supplemental Notes

Note S1. Discussion of the relationship between MDIC3 and GraphFP

First, similar to MDIC3, GraphFP[1] also does not use the L-R databases in its cell-cell interaction prediction process, and its cell-cell interaction prediction results are also overall results. The difference is that GraphFP is a nonlinear Fokker-Planck equation graph based model and dynamic inference framework, and considers the cell-cell interaction as a non-linear quadratic term. MDIC3 is a matrix decomposition model. An expression matrix of single cells contains regulatory information among genes and intercellular crosstalk information. Therefore, matrix decomposition can be used to uncover the regulatory or crosstalk patterns among cells from the expression matrix.

Secondly, GraphFP predicts interactions among different cell types and incorporates cell type information into the prediction process. In contrast, MDIC3 provides communication results among all individual cells. By integrating the results of communication among individual cells. MDIC3 can also obtain communication results among different cell types.

Finally, GraphFP considers cell state transitions as a dynamic process and aims to reconstruct cell state transitions from time-series single-cell transcriptome data, delineating cell-cell interactions that drive cell differentiation, focusing on the changes in cellular communication during the cell state transitions dynamic process. In contrast, MDIC3 primarily focuses on the analysis of cell-cell communication in a particular state. The article of GraphFP explores the changes in neuronal cell interactions at different time points during mouse development, which has parallels with our joint learning of skin development in E13.5 and E14.5 mouse. However, as MDIC3 does not rely on time-series single-cell transcriptome data, it has a wider range of applications compared to GraphFP. For example, the results of MDIC3 can be further used for the joint analysis of cell-cell communications across different disease states (such as the "Joint learning of lesional and nonlesional human skin" section). In addition, since GraphFP is an algorithm designed for predicting cell-cell interactions based on a large amount of dynamic time-series data, it is more suitable for the analysis of time-series data with multiple time points. It may prove more advantageous when exploring changes in cell-cell interactions at different time points.

Note S2. Exploring the effect of different gene coverage on prediction results

The proportion of zeros contained in the human islet dataset matrix we used was 60.96%. To investigate whether different gene coverage affects the prediction results of MDIC3, we randomly selected non-zero elements from the human islet dataset matrix and replaced them with zeros. This process resulted in the creation of three new matrices (Matrix1, Matrix2, Matrix3), containing 70.01% (Matrix1), 80.08% (Matrix2), and 91.25% (Matrix3) of the proportion of zeros, respectively. We re-inferred the cell-cell communications based on these three new matrices using MDIC3, and separately calculated the literature support rates from the results obtained from each matrix.

The literature support rate for the original human islet dataset matrix is 83.3% (10/12) and the literature support rate gradually decreases as the proportion of zeros in the matrix increases: 76.92% (10/13) for Matrix1, 76.92% (10/13) for Matrix2 and 60% (9/15) for Matrix3. These results indicate that the prediction effectiveness of MDIC3 can be affected by the drop-out effect or gene coverage of single cell data, but the fluctuation of the effect is acceptable. When the proportion of zeros in the single cell data reaches 90% or more, the literature support rates for the results predicted by MDIC3 remain over 50%, implying that the MDIC3 algorithm is stable and robust, and the results obtained from different gene coverage are also acceptable.

Note S3. Benchmark against ligand-receptor pairing accuracy

Considering that the CellChat, Cellphonedb, and iTALK algorithms are all L-R-based algorithms with their own L-R databases, we first derived significant L-R pairs obtained by applying each of the three algorithms to the human lesional skin dataset. Then, we obtained the average expression of all significant L-R pairs for each pair of cell type pairs, separately. Finally, we calculated the correlation between the expression results of each cell type pair under the three algorithms separately and the communication strength of each cell type pair obtained by MDIC3.

We applied this benchmarking approach to the human lesional skin dataset. The correlation of the L-R expression results between existing tools (CellChat, CellPhonedb, and iTALK) and MDIC3 with 49 cell types are (PCC: 0.335, p-value: 0.018), (0.414, p-value: 0.003), and (0.418, p-value: 0.002), respectively. This suggests that the results from MDIC3 are significantly correlated with the overall expression of L-R pairs.

Note S4. Benchmarking of how well the matrix decomposition actually work

Given a single-cell gene expression matrix A with size $m \times n$ as input, the matrix A contains m genes and n cells. MDIC3 inferred cell-cell communication through matrix decomposition, dividing the original single-cell gene expression matrix A into three matrices R, Σ, and W. The matrix R uses the adjacency matrix of the gene regulatory network (GRN) obtained by the publicly available algorithm GNIPLR[2], which calculates a unique GRN adjacency matrix. The matrix Σ is the complete singular value matrix of matrix A. The matrix W is the cell-cell communication adjacency matrix and is the only result obtained using the Moore-Penrose pseudo-inverse. We consider that these three matrices R, Σ, and W are able to reconstruct the original single-cell gene expression matrix A. We calculated the Mean Squared Error (MSE) between the reconstructed matrix $R\Sigma W$ and the original single-cell gene expression matrix A using the human lupus nephritis dataset, the E14.5 mouse skin dataset, and the mouse brain dataset respectively. The MSE is calculated as follows:

$$MSE = \frac{1}{m \times n}\sum_{i=1}^{m\times n}(A_i - (R\Sigma W)_i)^2$$

The human lupus nephritis dataset contains 2838 cells and 22447 genes. The E14.5 mouse skin

dataset contains 12179 cells and 14077 genes. The mouse brain dataset contains 3005 cells and 19972 genes. The MSEs between the reconstructed matrix $R\sum W$ and the original single-cell gene expression matrix A under the human lupus nephritis dataset, the E14.5 mouse skin dataset, and the mouse brain dataset are 0.22, 0.25, and 0.24 respectively.

Note S5. The computational cost of MDIC3

We have tested the MDIC3 algorithm in terms of running time and running memory by simulating single-cell expression matrices with different sizes. The simulation steps are as follows:

(1) Randomly generate a matrix A of a given size, with the number of rows m denoting the number of genes simulated and the number of columns n denoting the number of cells simulated.

(2) Randomly generate a matrix R of size $m \times m$ as the simulated GRN adjacency matrix.

(3) Calculate the complete singular value matrix $\sum$ of A, multiplying R and $\sum$.

(4) Calculate the $(R\sum)^{+}A$ as the simulated cell-cell communication adjacency matrix W.

We tested the computational costs of MDIC3 with different simulation matrices containing 20,000 genes and 100 to 5,000 cells and recorded the total running time and peak memory usage for the simulation. The results are shown in Table S8.

We found that when the number of genes reached 20,000 and the number of cells reached 5,000, the computation time for MDIC3 was 4.96 minutes, and the memory consumption was 10.4GB. As the matrix size increases, the memory required for MDIC3 calculations also increases.

We also recorded the running time and memory increments for the human lesional skin cell dataset, E14.5 mouse skin dataset, and mouse brain dataset in Table S9. The E14.5 mouse skin dataset cost the longest running time (about 16 min), and both the E14.5 mouse skin dataset and the mouse brain dataset cost similar memory consumption (about 23GB). It should be noted that the full running cost of MDIC3 is focused on the calculation of GRN using the GNIPLR algorithm. Considering that any tool that infers regulatory networks can be used for MDIC3, and MDIC3 is not limited to GNIPLR, here, we only recorded the running time and memory increments for MDIC3 to solve for cellular communication after the GRN was calculated.

We also recorded the running time and memory increments during the run of the CellChat algorithm on the three datasets. The running time of CellChat on the human lesional skin cell dataset is 1.367 min, and the memory increments are 16.5kb. The running time of CellChat on the E14.5 mouse skin dataset is 7.316 min, and the memory increments are 16kb. The running time of CellChat on the mouse brain dataset is 2.66 min, and the memory increments are 29.2 MiB. After comparison, we found that MDIC3 takes longer to run, requires more running memory, and costs more to run. However, in general, we consider these computational costs acceptable for a personal computer.

Note S6. Details for calculating gene regulatory network (GRN)

Accurate and fast calculation of GRN is critical for MDIC3. Supposing the single-cell gene

expression matrix is $A_{m\times n}$ with m cells and n genes, and the adjacency matrix of GRN is $R_{m\times m}$ from single-cell gene expression. We made some optimizations to the calculation process of the adjacency matrix $R_{m\times m}$.

Step 1: Grouping genes.

The m genes in the single-cell gene expression data can be represented as $g_1, g_2, \cdots, g_m$. We divided m genes into l groups in order from g_1 to g_m, each group contains u genes, $u < m$. To be more specific, the u genes contained in the first gene group G_1 are g_1, g_2, $\cdots$, g_u, the u genes contained in the second gene group G_2 are g_{u+1}, g_{u+2}, $\cdots$, g_{2u}, so on, and genes contained in the l-th gene group G_l are $g_{(l-1)\times u+1}$, $g_{(l-1)\times u+2}$, $\cdots$, g_m.

Step 2: Compute the adjacency matrix through submatrices.

We divide the matrix $R_{m\times m}$ into l^2 blocks and R can be expressed as:

$$R = \begin{pmatrix} R_{11} & R_{12} & \cdots & R_{1l} \\ R_{21} & R_{22} & \cdots & R_{2l} \\ \vdots & \vdots & \cdots & \vdots \\ R_{l1} & R_{l2} & \cdots & R_{ll} \end{pmatrix}$$

Each block R_{ij} is a GRN adjacency submatrix with size $u \times u$ and represents the regulation from the u genes contained in the i-th gene group G_i to the u genes contained in the j-th gene group G_j. So, we convert the problem of calculating the adjacency matrix $R_{m\times m}$ into calculating l^2 adjacency submatrices.

All adjacency submatrix calculations in this paper use the GNIPLR[2] algorithm, where the regularization parameter $\alpha = 0.1$.

Step 3: Integrate to get the complete adjacency matrix.

Integrate all the l^2 submatrices and obtain the complete GRN adjacency matrix $R_{m\times m}$.

Note S7. Details of the aggregated communications

The different tools use different strategies to present their inference communication strength. We used the communication probability inferred by CellChat, the L-R average gene expression inferred by CellPhoneDB, and the L-R average gene expression inferred by iTALK as the inferred communication strength of these three tools. These measures are the default communication strength for the three existing algorithms. The results obtained from each tool were independent, and we did not normalize the communication strengths across the different tools. Furthermore, since none of the three tools mentioned thresholds for inferring results in their articles or program packages, we used the mean value of communication strengths as a unifying criterion. In other words, we calculated the mean value of communication intensity as the threshold for each tool, and our results follow the same way of calculating thresholds.

Details of obtaining the aggregated communication results from CellChat.

Use R package CellChat v1.1.3[3] to infer intercellular communications in scRNA-seq datasets.

The calculation process for each dataset uses default parameters.

The R package of CellChat can calculate the aggregated cell-cell communication results by summarizing the communication probability under each pathway or L-R pair between any two cell types. The aggregated communication results can be directly derived by the R package of CellChat.

Details of obtaining the aggregated communication results from Cellphonedb.

Use Python package Cellphonedb v2.0.0[4] to infer intercellular communications in scRNA-seq datasets.

Use the default command "cellphonedb method statistical_analysis meta.txt counts.txt [--counts-data=gene_name] --iterations=100 --threads=2"

Cellphonedb derives the means of the averaged ligand expression of cells in the ligand cell type and the averaged receptor expression of cells in the receptor cell type under a specific ligand-receptor (L-R) pair and considers the derived result as the communication strength between the two cell types under the specific L-R pair. As the same with CellChat, we summed the communication strengths under each L-R pair as the aggregated communication strength between any two cell types.

Details of obtaining the aggregated communication results from iTALK.

Use R package iTALK v0.1.0[5] to infer intercellular communications in scRNA-seq datasets.

The calculation process for each dataset uses default parameters.

iTALK calculates the averaged ligand expression of cells in the ligand cell type and the averaged receptor expression of cells in the receptor cell type under the specific L-R pair. Like Cellphonedb, we first averaged the mean expression values of ligand and receptor and consider this averaged result as the communication strength between two cell types under the specific L-R pair. Then, as the same with CellChat, the aggregated communication strength between any two cell types will be calculated by summing the communication strength under each L-R pair.

Note S8. Details of the two-side self-representation model

The two-side self-representation model is a matrix decomposition model. Given a single-cell gene expression matrix A with size $m \times n$ as input, the matrix A contains m genes and n cells. The one-side self-representation model of matrix A can be represented as

$$A = ZA \quad \text{(S1)}$$

$$A = AX \quad \text{(S2)}$$

Where matrix Z with size $m \times m$ is the similarity between genes and matrix X with size $n \times n$ is the similarity between cells.

Combined the equation (S1) and (S2), we can obtain the two-side self-representation model to factorize the single-cell gene expression matrix A:

$$A = ZAX \quad \text{(S3)}$$

Then the matrix A can be represented by itself and two other matrices Z and X. The matrix Z

is the left sub-matrix with the size of $m \times m$ and presents the regulatory relationship among genes, the m denotes the number of genes in single-cell expression matrix A; The matrix X is the right sub-matrix with the size of $n \times n$ and can be used to present the relationship among cells, the n denotes the number of cells in single-cell expression matrix A.

If we attempt to obtain the communication relationship among cells, we should figure out X from the equation (S3). The matrix Z can represent the regulatory relationship among genes, so we can use adjacent matrix of matrix A to replace the matrix Z, then matrix X can be solved from equation (S3). In Eqn. (S3), the two-side self-representation model can use the original single-cell expression matrix itself as the mediation matrix to infer the cell-cell communication network. So, the two-side self-representation model can obtain the cell-cell communication network by solving the matrix X:

$$X = (ZA)^{+} A \tag{S4}$$

Where $(ZA)^{+}$ represent the pseudoinverse matrix of (ZA) and can be computed by the Moore-Penrose pseudo-inverse[6] of matrix (ZA). The singular matrix of matrix A can be used to replace the matrix A in two-side self-representation model. Then, we can obtain a new matrix decomposition pattern for matrix A:

$$A = R \sum W \tag{S5}$$

Where the matrix R is the left sub-matrix with the size of $m \times m$ and presents the regulatory relationship among genes, the m denotes the number of genes in single-cell expression matrix A; The matrix W is the right sub-matrix with the size of $n \times n$ and can be used to present the relationship among cells, the n denotes the number of cells in single-cell expression matrix A; The matrix $\sum$ is the singular matrix of matrix A with dimension $m \times n$, which represents the mediation matrix connected the relationships between genes and cells.

Considering that both equation (S3) and (S5) can infer intercellular relationships based on intergenic relationships from single-cell expression profiles, we compared the results of (S3) and (S5) using different datasets. The literature support ratio in the results inferred by the (S5) under each dataset is always higher than the (S3) model (Figure 5A, Figure S5A and S5C). By comparing the final solution results of the two methods, the better performance of equation (S5) may be attributed to the fact that it uses the singular matrix as the mediation matrix, and the singular matrix can better extract the information containing the relationships between genes and cells, while the (S3) model uses the original single-cell expression matrix as the mediation matrix, which may contain additional noises.

Supplemental Reference

S1. Jiang, Q., Zhang, S., and Wan, L. (2022). Dynamic inference of cell developmental complex energy landscape from time series single-cell transcriptomic data. PLoS Comput. Biol. *18*, e1009821. 10.1371/journal.pcbi.1009821.

S2. Zhang, Y.L., Chang, X., and Liu, X.P. (2021). Inference of gene regulatory networks using pseudo-time series data. Bioinformatics *37*, 2423-2431. 10.1093/bioinformatics/btab099.

S3. Jin, S.Q., Guerrero-Juarez, C.F., Zhang, L.H., Chang, I., Ramos, R., Kuan, C.H., Myung, P., Plikus, M.V., and Nie, Q. (2021). Inference and analysis of cell-cell communication using CellChat. Nat. Commun. *12*, 20, 1088. 10.1038/s41467-021-21246-9.

S4. Vento-Tormo, R., Efremova, M., Botting, R.A., Turco, M.Y., Vento-Termo, M., Meyer, K.B., Park, J.E., Stephenson, E., Polanski, K., Goncalves, A., et al. (2018). Single-cell reconstruction of the early maternal-fetal interface in humans. Nature *563*, 347-+. 10.1038/s41586-018-0698-6.

S5. Yuanxin, W., Ruiping, W., Shaojun, Z., Shumei, S., Changying, J., Guangchun, H., Michael, W., Jaffer, A., Andy, F., and Linghua, W. (2019). iTALK: an R Package to Characterize and Illustrate Intercellular Communication. bioRxiv, 507871. 10.1101/507871.

S6. Strang, G. (1980). 3 - WITHDRAWN: ORTHOGONAL PROJECTIONS AND LEAST SQUARES. In Linear Algebra and its Applications (Second Edition), G. Strang, ed. (Academic Press), pp. 103-152. https://doi.org/10.1016/B978-0-12-673660-1.50006-X.
