# Supplementary material for: MDIC3: Matrix decomposition to infer cell-cell communication
Source: Patterns (N Y). 2024 Jan 11;5(2):100911. doi: 10.1016/j.patter.2023.100911 (PMC10873161; doi:10.1016/j.patter.2023.100911)
Supplement: Document S2. Article plus supplemental information [file mmc3.pdf]

Patterns

Article

MDIC3: Matrix decomposition to infer cell-cell communication

Graphical abstract

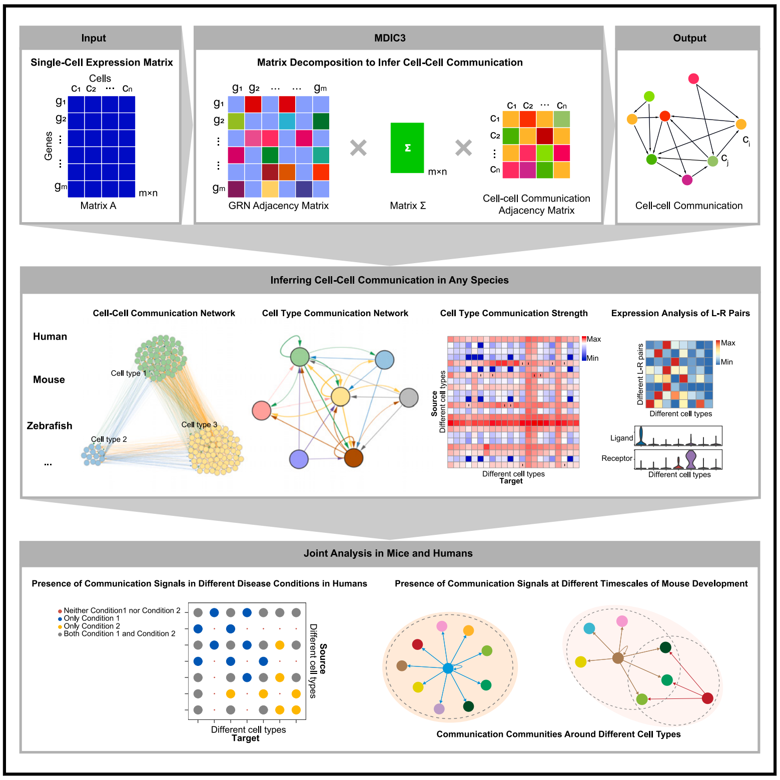

Highlights

- MDIC3 can be used to infer cell-cell communication without prior L-R information
- MDIC3 relies only on single-cell sequencing data and is species independent
- MDIC3 separates information about genes and cells from expression profiles
- MDIC3 contributes to improved cell-cell communication inference resolution

Authors

Yi Liu, Yuelei Zhang, Xiao Chang, Xiaoping Liu

Correspondence

chxlaugh@163.com (X.C.),
xpliu@ucas.ac.cn (X.L.)

In brief

The authors present MDIC3, an unsupervised method for inferring cell-cell communication without prior ligand-receptor information. MDIC3 separates information about genes and cells directly from expression matrices derived from single-cell sequencing data. The authors demonstrate the use of the method in mouse and zebrafish embryonic development and in skin samples from patients with atopic dermatitis.

Liu et al., 2024, Patterns 5, 100911
February 9, 2024 © 2023 The Author(s).
https://doi.org/10.1016/j.patter.2023.100911

CellPress

Patterns

CellPress
OPEN ACCESS

Article

MDIC3: Matrix decomposition to infer cell-cell communication

Yi Liu,[1,2] Yuelei Zhang,[2] Xiao Chang,[3,*] and Xiaoping Liu[1,4,*]
[1]Key Laboratory of Systems Health Science of Zhejiang Province, School of Life Science, Hangzhou Institute for Advanced Study, University of Chinese Academy of Sciences, Hangzhou 310024, China
[2]School of Mathematics and Statistics, Shandong University, Weihai 364209, China
[3]Institute of Statistics and Applied Mathematics, Anhui University of Finance and Economics, Bengbu 233030, China
[4]Lead contact
*Correspondence: chxlaugh@163.com (X.C.), xpliu@ucas.ac.cn (X.L.)
https://doi.org/10.1016/j.patter.2023.100911

THE BIGGER PICTURE Cell-cell communication (CCC) can reveal diverse aspects of life processes and cellular function. CCC involves information exchange among cells via ligand-receptor (L-R) pairs. Existing CCC studies rely on gathering L-R pairs to construct databases and analyze L-R gene co-expression. Building comprehensive L-R databases for different species, however, is challenging, especially considering the sensitivity of CCC inference to database completeness and accuracy. Methods of CCC that do not rely on L-R lists will have opportunities to discover more comprehensive CCC across different species. This holds great practical significance for exploring the functional interactions among cells in different biological states, understanding the biological reasons behind the therapeutic effects of various drugs on diseases for drug development, and elucidating CCC between different species (such as understanding the CCC among hosts and microorganisms to elucidate infection mechanisms).

1 2 3 4 5 **Development/Pre-production:** Data science output has been rolled out/validated across multiple domains/problems

SUMMARY

Crosstalk among cells is vital for maintaining the biological function and intactness of systems. Most existing methods for investigating cell-cell communications are based on ligand-receptor (L-R) expression, and they focus on the study between two cells. Thus, the final communication inference results are particularly sensitive to the completeness and accuracy of the prior biological knowledge. Because existing L-R research focuses mainly on humans, most existing methods can only examine cell-cell communication for humans. As far as we know, there is currently no effective method to overcome this species limitation. Here, we propose MDIC3 (matrix decomposition to infer cell-cell communication), an unsupervised tool to investigate cell-cell communication in any species, and the results are not limited by specific L-R pairs or signaling pathways. By comparing it with existing methods for the inference of cell-cell communication, MDIC3 obtained better performance in both humans and mice.

INTRODUCTION

Crosstalk among cells is a fundamental function that leads multicellular organisms to accomplish complex biological tasks.[1] For example, during early embryonic development, the cell's differentiation and ultimate fate are controlled by communication among neighboring cells.[2] In developed organisms, intercellular communications coordinate the activities of multiple cells for complex organismal processes such as immune response, growth, and homeostasis.[2] Cell-cell communication involves interactions between cells of different types, and it extensively relies on interactions between secreted ligands and cell-surface receptors.[2] Single-cell RNA sequencing (scRNA-seq) technologies can be used to detect the gene expression of each cell, leading to the discovery of cellular information at an unprecedented resolution level, thus greatly facilitating the development of intercellular communication research.[3–6] On the basis of scRNA-seq, the systematic deciphering of the intercellular crosstalk mediated by ligand-receptor (L-R) interactions quickly became a research focus.[3,4,7–10]

This is an open access article under the CC BY license (http://creativecommons.org/licenses/by/4.0/).

To date, many methods have been developed for cell-cell communication, such as CellPhoneDB,[7] CellChat,[4] and iTALK.[8] All of these methods investigate intercellular crosstalk on the basis of the expression intensity of specific L-R pairs, such as their co-expression (sum, mean, or product),[2,7] differential expression[8,11], and expression correlation.[12] However, relying on prior information such as L-R signals to investigate cell-cell communications is a common feature of existing cell-cell communication inference, and it also limits the application of these existing methods to other species without specific L-R pairs or signaling pathways. Thus, a strategy for identifying the overall cell-cell communication potential is still lacking. To date, the majority of existing L-R interaction databases are dominated by human databases, which were manually curated on the basis of experimental studies or literature. One of the representative human L-R databases was compiled and expanded by Ramilowski et al.,[2] which presents the first large-scale map of cell-cell communication between 144 human primary cell types.[2] Subsequently, a more comprehensive human L-R database called CellPhoneDB was built, which considered the subunit architecture for both ligands and receptors.[7] More recently, an L-R database in both mice and humans was published by Jin et al.,[4] which also contains the pairs of L-Rs with corresponding signaling pathways. Although there are many L-R databases, they cannot guarantee that an L-R database contains all of the discovered L-R information. In addition, the ongoing exploration of unknown L-R interactions means that the update of the L-R databases never stops.

In this paper, we propose a new method, MDIC3 (matrix decomposition to infer cell-cell communication), to reveal cell-cell communication through the cooperative analysis of gene regulatory networks (GRNs) and matrix decomposition on the basis of scRNA-seq data. Importantly, our method is independent of prior knowledge, meaning that the inference of cell-cell communication is no longer limited to specific species and does not rely on predefined L-R interactions or signaling pathways. MDIC3 can investigate intercellular communication networks from individual cells to cell types on the basis of existing cell classification labels. We showed the overall capabilities of MDIC3 by applying it to both mouse and human scRNA-seq datasets and evaluated its reliability and effectiveness using a large body of literature. We applied MDIC3 to analyze the communication networks during the early embryonic development of mice and found that the dominant communication signaling during the developmental stages from embryonic day 13.5 (E13.5) to E14.5 changes from dermal cells to epidermal cells. Furthermore, we used MDIC3 for two datasets of lesional (LS) and non-lesional (NL) skin from atopic dermatitis (AD) patients to compare the communication networks in different disease conditions. Our findings indicate that inflammatory phenomena may be present in both LS and NL skin, but the communications among inflammatory cells are more active in LS skin.

RESULTS

Overview of MDIC3

MDIC3 is used to explore cell-cell communication from single-cell expression data. MDIC3 only requires a single-cell gene expression matrix when determining the cell-cell communication network, instead of any prior knowledge such as L-R pairs. Cell-cell communication can be regarded as a special regulatory relationship between cells. The core of MDIC3 lies in determining the regulatory relationships among cells on the basis of the regulatory relationships among genes. The regulatory relationships among genes can be depicted by the GRN, which can be determined from the single-cell gene expression profiles.

MDIC3 is based on the idea of matrix decomposition and tries to separate information about the genes and cells contained in the expression profile matrix. Usually, the single-cell gene expression profile with m genes and n cells can be regarded as a matrix A with size $m \times n$, and the matrix A can be further decomposed into three submatrices (Equation 4). The left submatrix R is the size of $m \times m$, the middle submatrix Σ is the size of $m \times n$, and the right submatrix W is the size of $n \times n$ (Figure 1). The left submatrix R is used to represent the regulatory relationships among genes and can be replaced by the GRN adjacency matrix with size $m \times m$. The middle submatrix Σ is used to connect the relationship between genes and cells and can be replaced by the singular matrix of the matrix A with size $m \times n$. The middle submatrix Σ represents the association information between genes and cells, as the singular matrix can extract the features of the matrix A. On the basis of the single-cell expression matrix A, the left submatrix R, and the middle submatrix Σ, we can obtain the right submatrix W by solving the pseudoinverse matrix (Equation 6). As the single-cell expression profile should contain the regulation information among genes and the crosstalk information among cells, the resolved right submatrix W reflects the regulatory relationships among cells, i.e., cell-cell communication (Figure 1; see methodology section). In fact, cell-cell communication can be regarded as a weighted directed graph/network among cells.

Investigating cell-cell communication in human lupus nephritis

Lupus nephritis (LN) is an autoimmune disease involving extensive cellular communication and is characterized by the infiltration of macrophages, T cells (TCs), and B cells into the kidneys.[13–17] Hence, we used MDIC3 to investigate intercellular communication in LN using a published human single-cell dataset[13] (Table S1). This dataset contains five kinds of cell groups that can be further divided into 22 cell types (Table S1), including the macrophage group (CM0, CM1, CM2, CM3, and CM4), T cell group (CT0a, CT0b, CT1, CT2, CT3a, CT3b, CT4, CT5a, CT5b, and CT6), B cell group (CB0, CB1, CB2a, CB2b, and CB3), dividing cell group (CD0), and epithelial cell group (CE0).

A total of 242 intercellular communications were identified among the 22 cell types by MDIC3, some of which are related to known L-R signaling. By comparing the results from MDIC3 with other tools (CellChat, CellPhoneDB, and iTALK), we found that some intercellular communications can be determined by both our algorithm and the other three tools. For example, the communications from CM4 to both CM2 and CM3 (Figure 2A) may be related to the expression of *CCL2-CCR2* (Figure 2B) L-R signaling and are involved in LN.[18] *CCL2*, a ligand with high expression in CM4, interacts with its receptor *CCR2*, which is highly expressed in both CM2 and CM3. Therefore, the communication from CM4 to both CM2 and CM3 can also be supported by *CCL2-CCR2* L-R signaling. It has been reported that *CCL2*, a

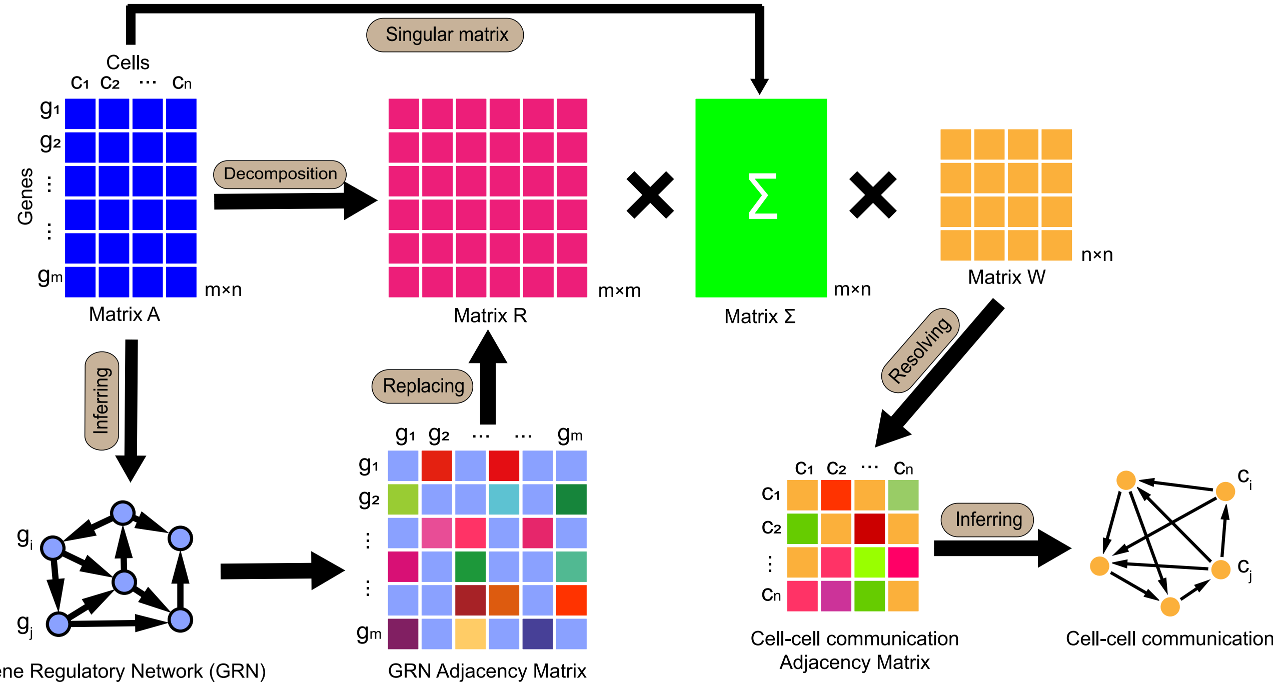

Figure 1. Overview of MDIC3

MDIC3 infers cell-cell communication from the input single-cell gene expression dataset with m genes and n cells. First, the gene expression matrix A can be decomposed into three submatrices, including a left submatrix R with size $m \times m$, a middle submatrix Σ with size $m \times n$, and a right submatrix W with size $n \times n$. Then, the middle submatrix Σ can be obtained by calculating the singular matrix of the gene expression matrix A. Next, the gene regulatory network (GRN) adjacency matrix is used to replace the left submatrix R, where the GRN can be inferred on the basis of the input gene expression matrix A and represented by an adjacency matrix. Finally, the right submatrix W can be resolved on the basis of the above three matrices, including the input gene expression matrix A, the left submatrix R represented by the GRN adjacency matrix, and the middle submatrix Σ represented by the singular matrix. The resolved right submatrix W was used as the cell-cell communication adjacency matrix, and the cell-cell communication network was further investigated.

macrophage chemokine, cooperates with its receptor *CCR2* to promote the recruitment of macrophages to sites of inflammation.[18–20] Studies have shown that the inhibition of *CCL2* may be a novel strategy for the treatment of LN.[20] In addition, the *CCL2-CCR2* signaling may also participate in the communication of CM4 to CB2b, which was detected by MDIC3, CellChat, CellPhoneDB, and iTALK (Figures 2A and S1), because of the high expression of the receptor *CCR2* in CB2b cells (Figure 2B).

It is worth noting that we also identified some intercellular communications that were determined only by our algorithm but did not appear in the results of all other tools. For example, communications from CE0 to CM0, CT1, and CT2 can be detected by MDIC3, CellChat, and iTALK (Figures 2A, S1A, and S1C) but not by CellPhoneDB (Figure S1B). This intercellular communication is consistent with the expression trends of *CX3CL1-CX3CR1* signaling, which plays a pathological role in inflammatory kidney disease[21,22] (Figure 2C). The ligand *CX3CL1* exhibits high expression trends in CE0, while the receptor *CX3CR1* shows high expression trends in CM0, CT1, and CT2. *CX3CL1* has been reported to be produced mainly by renal tubular epithelial cells, and its receptors, such as *CX3CR1*, can be found on leukocytes, including macrophages and TCs.[21–24] This indicates that epithelial cells may be involved in the infiltration of immune cells associated with nephritis, including macrophages and TCs.[21–24] MDIC3 also examines communication from CE0 to CD0, which was also detected by CellChat and iTALK (Figures S1A and S1C) but not by CellPhoneDB (Figures 2A and S1B). The intercellular communication of CE0 to CD0 is possibly related to the expression of *CX3CL1-CX3CR1* signaling, as we found not only that the ligand *CX3CL1* can be expressed by CE0 but also that the receptor *CX3CR1* can be expressed by CD0 (Figure 2C).

Additionally, MDIC3 can also reveal known intercellular communication between B cells and TCs, such as the communication from *CD40*-expressing CB3 to *CD40LG*-expressing CT0a, CT0b, and CT3b, which are involved in the immune regulation of LN[25–28] (Figures 2A and 2C). This communication can also be identified by CellChat and CellPhoneDB (Figures S1A and S1B) but not by iTALK (Figure S1C).

Moreover, we also identified communication from CB3 to CT5a, which appeared in the results of CellChat and CellPhoneDB (Figures S1A and S1B) but did not appear in the results of iTALK (Figures 2A and S1C). This communication is mediated by *CXCL10-CXCR3* L-R signaling. The signaling pathway was found to be significantly enriched in LN patients through biological experiments and immunofluorescence,[18,29] with high expression of the ligand *CXCL10* in CB3 and the receptor *CXCR3* in CT5a (Figure 2C). Furthermore, MDIC3 identified crosstalk among TCs, such as CT6 to CT0a and CT6 to CT5a (Figure 2A), which can also be found by CellChat (Figure S1A) but cannot be found by CellPhoneDB and iTALK (Figures S1B and S1C). This crosstalk was driven by *CXCL10-CXCR3* signaling (Figure 2C), as the ligand

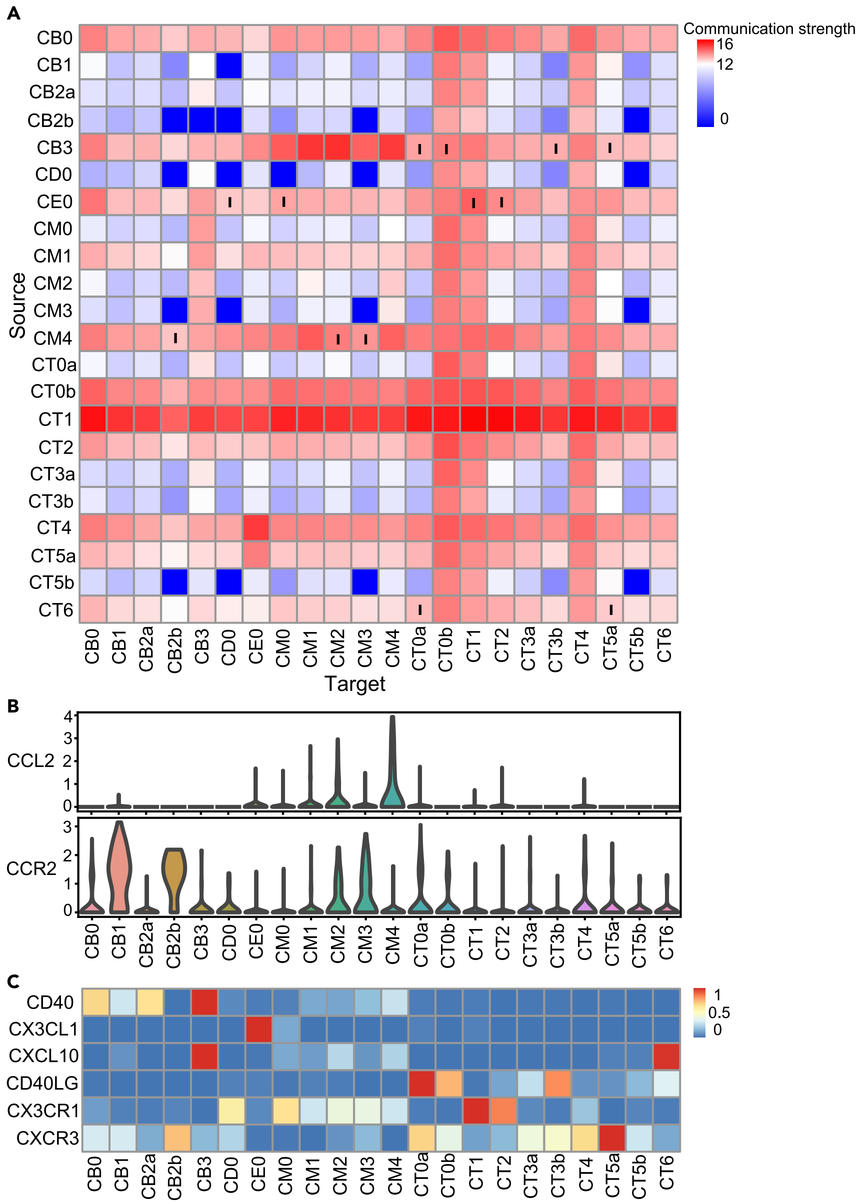

Figure 2. Case study on the human lupus nephritis dataset

(A) Heatmap showing the communication results among cell types determined by MDIC3. Black "I" indicates the communication mentioned in this main text. The row represents the "source" of a communication, and the column represents the "target" of the communication. For example, the black "I" in row "CB3" and column "CT0a" indicate intercellular communication from CB3 to CT0a.

(B) Violin plot shows the expression of the *CCL2-CCR2* ligand-receptor gene pair across different cell types in human lupus nephritis.

(C) Heatmap shows the expression of the *CD40-CD40LG*, *CX3CL1-CX3CR1*, and *CXCL10-CXCR3* ligand-receptor gene pairs across different cell types in human lupus nephritis.

CXCL10 has high expression in CT6, and the receptor *CXCR3* also has significant expression in CT0a and CT5a.

Investigating cell-cell communication in human LS skin

AD is an inflammatory skin disease involving skin immune and barrier abnormalities.[30–32] There are a variety of signaling pathways for immune-inflammatory responses and complex chemokine signaling changes in the AD skin microenvironment. We applied MDIC3 to investigate cell-cell communication in the human AD LS skin scRNA-seq dataset[30] (Table S1). Our findings revealed intercellular communications from the inflammatory FIB to immune cells (dendritic cells [DCs] and TCs) (Figure S2A; Table S1), which is in concordance with a previous study.[30] The inflammatory FIB may interact with immune cells to regulate lymphoid cell organization and type 2 inflammation.[30] Moreover, AD was reported to be associated with the expression of periostin (*POSTN*), and cells expressing *POSTN* have been reported to have direct signaling with immune cells.[33] As expected, the cells expressing *POSTN* were predominantly present in the inflammatory FIB (Figure 3A).

DC groups, including inflammatory DCs, cDC1s, cDC2s, and Langerhans cells (LCs), represent one of the immune cell groups (Table S1). MDIC3 identified communication from inflammatory FIB to inflammatory DC (Figure S2A), a finding consistent with other methods (Figures S2B–S2D) and supported by the *CCL19-CCR7* L-R signaling (Figure S2E), as the ligand *CCL19* and its receptor *CCR7* were only highly expressed in inflammatory FIB and inflammatory DC, respectively (Figure S2E). The communication from inflammatory FIB to inflammatory DC through *CCL19-CCR7* L-R signaling is also in agreement with the immunofluorescence staining result in the original study.[30] Moreover, MDIC3 reveals communication from inflammatory FIB to other cell types of the DC cell group, such as inflammatory FIB to cDC1, inflammatory FIB to cDC2, and inflammatory FIB to LC (Figure S2A). These intercellular communications can be explained in the pathways known to be involved in immune and inflammatory responses.[4] For example, the expression of L-R pairs from the COMPLEMENT[4] pathway, such as *C3* to (*ITGAM*+*ITGB2*) and *C3* to (*ITGAX*+*ITGB2*), was found to contribute to the communication from inflammatory FIB to cDC1, cDC2, and LC (Figure 3B). Moreover, we observed that the communications from inflammatory FIB to cDC2s and LCs can also be inferred by CellChat, CellPhoneDB, and iTALK. Additionally, the communication from inflammatory FIB to cDC1 appeared in the results of CellPhoneDB and iTALK but not in the results of CellChat (Figures S2B–S2D).

The TC group, including inflammatory TC and natural killer T (NKT) cells, represents another immune cell group (Table S1).

CellPress
OPEN ACCESS

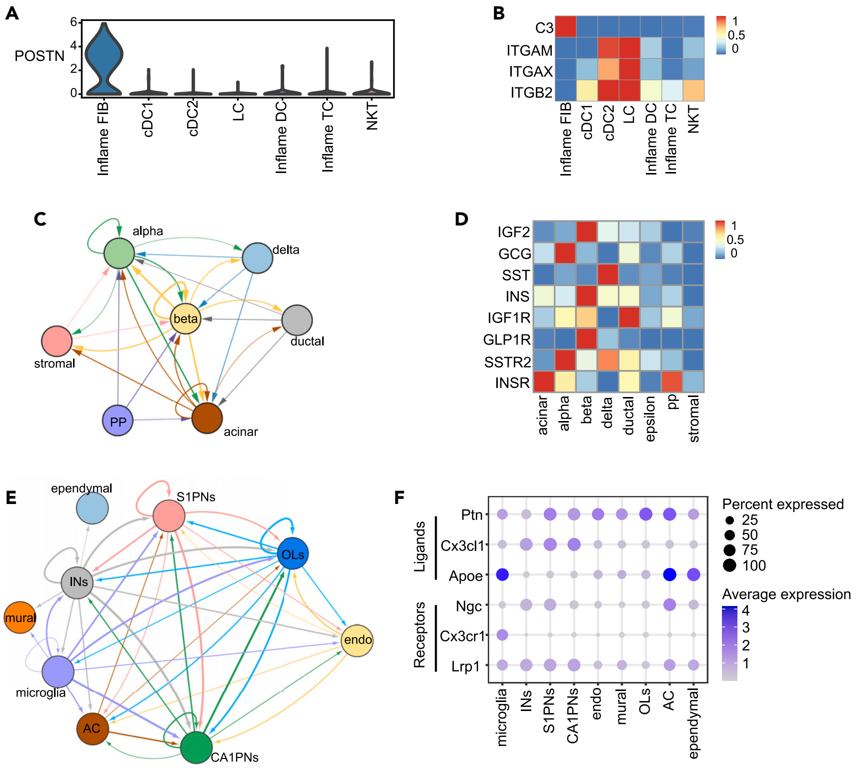

Figure 3. Case study on the human lesional skin dataset, human islet dataset, and mouse brain dataset

(A) Violin plot shows the expression of POSTN across different cell types in human lesional skin.

(B) Heatmap shows the expression of selected ligand-receptor gene pairs under the COMPLEMENT signaling pathway in human lesional skin, including *C3*-(*ITGAM*+*ITGB2*) and *C3*-(*ITGAX*+*ITGB2*). Inflame FIB, inflammatory fibroblasts; cDC, conventional dendritic cell; inflame DC, inflammatory dendritic cell, LC, Langerhans cell; inflame TC, inflammatory T cell; NKT, natural killer T cell.

(C) Communication network among cell types in human islets inferred by MDIC3. The direction of the arrow indicates the direction of signal transference. The edge width represents the communication strength.

(D) Heatmap showing the expression of ligand-receptor signaling (*IGF2-IGF1R*, *GCG-GLP1R*, *SST-SSTR2*, *INS-INSR*) across different cell types in different human islet cells.

(E) Communication network among cell types in the mouse brain inferred by MDIC3. The direction of the arrow indicates the direction of signal transference. The edge width represents the communication strength.

(F) Dot plot depicting the percent expression and average expression of selected ligand-receptor gene pairs (*Ptn-Ngc*, *Cx3cl1-Cx3cr1*, *Apoe-Lrp1*) in different mouse brain cell types. S1PNs, S1 pyramidal neurons; CA1PNs, CA1 pyramidal neurons; INs, interneurons; OLs, oligodendrocytes; AC, astrocytes; endo, vascular endothelial cells.

MDIC3 inferred the communication from inflammatory FIB to inflammatory TC (Figure S2A), a finding consistent with the original analysis.[30] However, the communication signal from the inflammatory FIB to the inflammatory TC can be identified by CellPhoneDB (Figure S2C) and iTALK (Figure S2D) but not by CellChat (Figure S2B). Notably, paracrine effects from inflammatory FIB to NKT cells were reported in the results of MDIC3 (Figure S2A) and iTALK (Figure S2D) but not in the results of CellChat (Figure S2B) or CellPhoneDB (Figure S2C). This paracrine effect can be explained by previous immunofluorescence staining,[34] which showed that *CXCR4*-expressing NKT cells can interact with *CXCL12*-expressing FIB, contributing to the development of allergic skin inflammation in AD patients.[34] Here, the ligand *CXCL12* was expressed only by inflammatory FIB, while NKT cells indeed expressed the receptor *CXCR4* (Figure S2F).

Investigating cell-cell communication in human islets

We used the human islet scRNA-seq dataset to further demonstrate the effect of MDIC3.[35] The five different types of endocrine cells can receive signals from the internal and external environment by autocrine and paracrine mechanisms or contact each other by membrane-bound molecules to form a complex intercellular communication network to complete biological functions, such as maintaining glucose homeostasis and regulating the state of surrounding cells.[36–38] MDIC3 revealed autocrine and paracrine effects focused on endocrine cells, especially cell-cell communication in alpha, beta, and delta cells (Figures 3C and S3A). We compared the findings of MDIC3 with those of other methods (CellChat, CellPhoneDB, and iTALK) (Figures S3B–S3D).

As a result, MDIC3 successfully detected autocrine signaling in beta cells (Figure 3C), which is consistent with existing research showing that beta cells can regulate insulin-mediated gene expression and protein translation in an autocrine manner.[39] Furthermore, autocrine signaling in beta cells may be mediated by *IGF2-IGF1R* L-R signaling, with the ligand *IGF* and receptor *IGF1R* being highly expressed in beta cells (Figure 3D). Indeed, previous studies have elucidated that insulin-like growth factor 2 (*IGF2*) is produced and secreted by human beta cells and is an autocrine activator of insulin-like growth Factor 1 receptor (*IGF1R*) signaling in beta cells.[40] Autocrine factors have important regulatory effects on the proliferation and function of beta cells.[41,42] Notably, autocrine signaling in beta cells was uniquely identified by MDIC3 but not by CellChat, CellPhoneDB, or iTALK (Figures S3B–S3D). This indicates that MDIC3 exhibits greater accuracy than other methods in identifying the autocrine signaling of beta cells. Moreover, the communications from beta cells to ductal cells were detected by MDIC3, CellChat, and CellPhoneDB (Figures 3C, S3B, and S3C) but not by iTALK (Figure S3D). This observation may be associated with the expression of *IGF2-IGF1R* L-R signaling (Figure 3D), attributed to the significant secretion of receptor *IGF1R* in ductal cells.

In addition, MDIC3 was able to identify paracrine communication from delta cells to alpha cells (Figure 3C). Our analysis revealed high expression of somatostatin (*SST*) in delta cells and the receptor *SSTR2* in alpha cells (Figure 3D). This suggests

that the communication from delta cells to alpha cells may be mediated by *SST*-*SSTR2* L-R signaling. Furthermore, our findings are in concordance with previous studies demonstrating that *SST*, secreted by delta cells,[43] can target alpha cells through the receptor *SSTR2* to inhibit glucagon secretion.[43–48] Notably, the paracrine communication from delta cells to alpha cells was not identified by CellChat, CellPhoneDB, and iTALK (Figures S3B–S3D).

MDIC3 also predicted bidirectional paracrine communication between beta cells and alpha cells (Figure 3C). Previous studies have indicated that alpha cells and beta cells are spatially close, and when islets are dispersed into individual cells, most beta cells remain attached to alpha cells.[36,37] This close spatial proximity suggests that beta cells and alpha cells may be directly exposed to each other's secretions, enabling their paracrine communication.[36,49] This direct communication plays a role in controlling the release of biosynthetic and secretory products, as well as cell survival.[50] For example, research has shown that ligand molecules (*GCG* and *INS*) secreted by alpha and beta cells can act as paracrine signals.[49,51–55] We found that the receptor *GLP1R* of *GCG* and the receptor *INSR* of *INS* were indeed significantly expressed on beta cells and alpha cells, respectively. Therefore, bidirectional communication may be mediated by *GCG*-*GLP1R* and *INS*-*INSR* L-R signaling (Figure 3D). Consequently, both spatial proximity and the expression of L-R pairs support our prediction of bidirectional paracrine communications. However, none of the bidirectional communications were predicted by CellChat, CellPhoneDB, or iTALK (Figures S3B–S3D).

Investigating cell-cell communication in E14.5 mouse skin

MDIC3 can analyze intercellular communication in any species because it does not require any prior knowledge, such as L-R databases. To illustrate the broad application of MDIC3, we showed the ability of MDIC3 to identify cell-cell communications in mouse datasets. As CellPhoneDB and iTALK contain L-R information only for humans, they cannot be used to investigate cell-cell communication in mouse data. Therefore, we compared the intercellular communications identified by MDIC3 with those from CellChat, which provides a mouse L-R database. Embryonic mouse skin development involves the proliferation and growth of numerous epidermal and dermal cells and the activation and transduction of various signals that drive the entire developmental process. We analyzed the scRNA-seq dataset of E14.5 embryonic mouse skin,[56] which included dermal cells and epidermal cells.[4] In this mouse dataset, we found some known communications between dermal cells (e.g., FIB-A, FIB-B, FIB-P, DC) and epidermal cells (e.g., basal, basal-P).

Both MDIC3 and CellChat can investigate autocrine communication for basal-P and paracrine communication from basal-P to FIB-A, FIB-B, FIB-P, and DC (Figures S4A and S4B). It has been reported that WNT signaling, generated by the epidermis, is needed for fibroblast proliferation during hair follicle development and drives the crosstalk between the epidermis and dermis through epidermal autocrine and epidermis-to-dermis paracrine signaling to mediate the role of hair follicle development.[57,58] This evidence supports the results showing that epidermal cells, such as basal-P, not only engage in autocrine communication but also in paracrine communication with some dermal cells, such as FIB-A, FIB-B, FIB-P, and DC (Figures S4A and S4B). In addition, we analyzed *Wnt6*-(*Fzd2*+*Lrp6*) L-R signaling, which has a high contribution rate to WNT signaling according to CellChat, and found that its expression can also support paracrine communication from basal-P cells to FIB-A, FIB-B, FIB-P, and DCs (Figure S4C). In other words, basal-P cells exhibit high expression of the ligand *Wnt6*, while FIB-A, FIB-B, FIB-P, and DC cells show high expression of the receptors *Fzd2* and *Lrp6*.

Both MDIC3 and CellChat also found that some epidermal cells (e.g., basal, basal-P) and some dermal cells (e.g., FIB-B, FIB-P, DC) responded to one fibroblast population, FIB-A, suggesting a paracrine effect in FIB-A. Additionally, we also observed an autocrine manner in FIB-A (Figures S4A and S4B), consistent with reports on the autocrine communication of FIB-A.[59–65] The FGF pathway is a known fibroblast growth signaling pathway and is crucial for skin morphogenesis.[59–65] Furthermore, the FGF pathway plays a regulatory role in hair follicle development, and its activation is essential for hair growth.[59–65] We found that the ligand *Fgf7* can promote the growth and proliferation of epidermal cells under the FGF pathway[65] and participates in paracrine communication from FIB-A to epidermal cells (e.g., basal, basal-P) through its receptor *Fgfr2*, as well as autocrine communication of FIB-A and paracrine communication from FIB-A to some other dermal cells (e.g., FIB-B, FIB-P, DC) through the receptor *Fgfr1* (Figure S4D). These observations further underscore the accuracy of MDIC3.

Investigating cell-cell communication in the mouse brain

As the second case study for mouse cell-cell communication, we used a scRNA-seq dataset from a juvenile mouse,[66] including neuron cells (S1PNs, CA1PNs, and INs), vascular endothelial cells (endo), and glial cells represented by microglia.[66] Communication among these cells is critical for the development and function of a normal brain, and their dysregulation can result in neurological disorders.[67–72]

MDIC3 found communication from INs to microglia (Figure 3E), which was not observed in the results of CellChat (Figure S4E). Communication from neuron cells to microglia was reported primarily through *Cx3cl1*-*Cx3cr1* signaling,[73,74] which regulates the apoptosis, proliferation, transcription, and migration of microglia.[75–77] We analyzed *Cx3cl1*-*Cx3cr1* signaling and found that it is consistent with the known specific localization of this signaling[74]; that is, *Cx3cl1* is abundantly expressed on INs, while its receptor *Cx3cr1* is restricted to microglia (Figure 3F).

Additionally, MDIC3 identified communications from microglia to S1PNs, CA1PNs, and INs (Figure 3E), which is consistent with the critical role for microglia in promoting neurogenesis to regulate neural differentiation and synaptic structure,[78–82] yet these interactions were not identified by CellChat (Figure S4E). Indeed, Apoe is an important secreted protein in the brain, and our analysis revealed its high expression in microglia (Figure 3F). Previous research has reported that Apoe is enriched in microglia and overlaps with the signal of the receptor *Lrp1* by immunofluorescence in the brain,[67] while the receptor *Lrp1* is indeed abundantly expressed on S1PNs, CA1PNs, and INs (Figure 3F). Interestingly, bidirectional communications between INs and microglia were identified by MDIC3 (Figure 3E) but not by CellChat (Figure S4E). However, the bidirectional communications between neurons

and glial cells are the basis for the function of the central nervous system and have been reported by previous studies.[75,82,83]

Furthermore, MDIC3 can also identify the autocrine communication of S1PNs and paracrine communication from S1PNs to INs (Figure 3E), which were not detected in the results of CellChat (Figure S4E). These communications may be related to *Ptn*, another important neuromodulator in the brain, which was found to be highly expressed in S1PNs (Figure 3F). Meanwhile, the receptor *Ngc*,[84,85] known for promoting neurite outgrowth,[84,85] was found to be highly expressed in both S1PNs and INs (Figure 3F). Notably, the *Ptn-Ngc* signal[84,85] was not documented in the CellChat L-R database, indicating that methods based on the L-R database may lack the ability to capture new signaling pathways for cell-cell communication.

Moreover, MDIC3 also showcases the communications from neuron cells (S1PNs, CA1PNs, and INs) to endo (Figure 3E), which is consistent with the role of neurons in directing vascular endothelial cell development.[67,86,87] For example, communication from INs to endo may be driven by the vascular endothelial growth factor (VEGF) pathway, such as *Vegfb-Flt1* L-R signaling (Figure S4F). However, the communications from neuron cells to endo cells were not fully identified by CellChat (Figure S4E).

Joint learning of E13.5 and E14.5 mouse skin

We used MDIC3 to detect communication signaling changes at different stages of mouse development, specifically focusing on the E13.5 and E14.5 mouse skin scRNA-seq datasets.[56] We first compared the ratio of cell types in E13.5 and E14.5 mouse skin (Figure 4A). The ratio of spinous epithelial cells (spinous) in E13.5 mouse skin was only 1.45%, while it significantly increased to 11.46% in E14.5 mouse skin (11.46%). In addition, two cell types (DC and pericytes) were found only in E14.5 mouse skin (Figure 4A). By examining the sending and receiving of communication signals (Figure 4B), we found that DCs and pericytes can both receive communication signals from different dermal and epidermal cell types, thus participating in E14.5 mouse skin cell-cell communications. In particular, DCs and pericytes also send communication signals, which are exclusively received by spinous epidermal cells (spinous) (Figure 4B). Notably, spinous exhibited extensive proliferation at E14.5[88] and showed enriched cellular communication signals specific to E14.5 (Figure 4B). Similarly, the proliferative basal cells (basal-P) also displayed abundant E14.5-specific cellular communication signals (Figure 4B). With the change in mouse embryonic development, the gradual activation of the two epidermal cell types (spinous and basal-P) is consistent with the evident epidermis stratification and differentiation program and the signs of basal differentiation around E14.5.[88,89] Furthermore, MDIC3 revealed E14.5-specific bidirectional paracrine communication between spinous and basal-P (Figure 4B). This E14.5-specific bidirectional paracrine effect may be related to the spatial proximity of proliferative basal cells and spinous epidermal cells around E14.5.[88]

At E13.5, dermal cells accounted for a large proportion of the cell number, and most cell-cell communication occurred among different dermal cell types, with fibroblasts (FIB-A, FIB-B, and FIB-P) showing high activity (Figures 4A and 4B). Intriguingly, over a one-day period, the communications among dermal cells and epidermal cells significantly increased, suggesting that epidermal cells gradually participate in cell-cell communication between E13.5 and E14.5 (Figure 4B). This finding may be related to the development of mouse hair follicles,[90] as previous studies showed that vibrissae development progressed dramatically from E13.5 to E14.5.[89] Histology analysis revealed the presence of primary follicles and stratified epithelium structures in the dorsal skin of E16.5 mice.[91] We also found that 41 cell-cell communications occurred among different cell types at both E13.5 and E14.5 (Figure 4B). These communications are essential for both time points of embryonic mouse skin development, but these communications may not be critical at specific time points.

Finally, we examined the top 10 communication strength edges on the basis of E13.5 and E14.5 communication networks among cell types and visualized them as two connectivity-summary networks (Figures 4C and 4D). We found different communication communities for both E13.5 and E14.5 (Figures 4C and 4D). For E13.5, we observed a community centered around dermal cells, represented by muscle cells (muscle), which dominated the outgoing communication signals at E13.5 (Figure 4C). Interestingly, these communications from muscle to other cell types also occurred in E14.5 (Figure 4B), suggesting that the communication activity of muscle may gradually decrease in later mouse development, possibly related to mouse abdominal wall development during mouse embryonic development.[92] Instead, E14.5 exhibited two communication communities with epidermal cells as centers, represented by spinous epidermal cells (spinous) and proliferative basal cells (basal-P), respectively, and they dominated the outgoing communication signals at E14.5 (Figure 4D).

In general, our joint analysis using MDIC3 and the E13.5 and E14.5 mouse skin datasets indicate that in the early stage of mouse development, cellular communications may be dominated by dermal cells, while with the development of mouse embryos, epidermal communication signals may increase. Taken together, MDIC3 enables a comprehensive assessment of cellular communication at different timescales.

Joint learning of LS and NL human skin

We also applied MDIC3 to analyze cell-cell communications in two different AD conditions. One dataset originates from LS (disease) skin of AD patients, while the other is derived from the NL (normal) skin of AD patients. Both datasets encompass 7 cell types (Table S1). Compared with NL skin, the three inflammatory cell types (inflame FIB, inflame DC, and inflame TC) exhibit a greater number of cellular communications in LS skin (Figure 4E) potentially linked to the presence of inflammation in the lesioned skin. AD lesions are characterized by expanded inflammatory cells that may interact with immune cells to regulate lymphoid cell organization and type 2 inflammation.[30] Notably, we identified LS-specific communication in which the signal was sent from inflammatory FIB and received by inflammatory DC (Figure 4E). This finding is consistent with the experimental results reported in the original study.[30]

Furthermore, we found that 8 cell-cell communications appeared in both NL skin and LS skin among different immune cell types (inflame DC, inflame TC, cDC1, cDC2, LC, and NKT) (Figure 4E), suggesting that cellular communications in different AD conditions are always closely linked to the immune response. Notably, we noticed that 14 communication signals related to inflammatory cells (inflame FIB, inflame DC, and inflame TC)

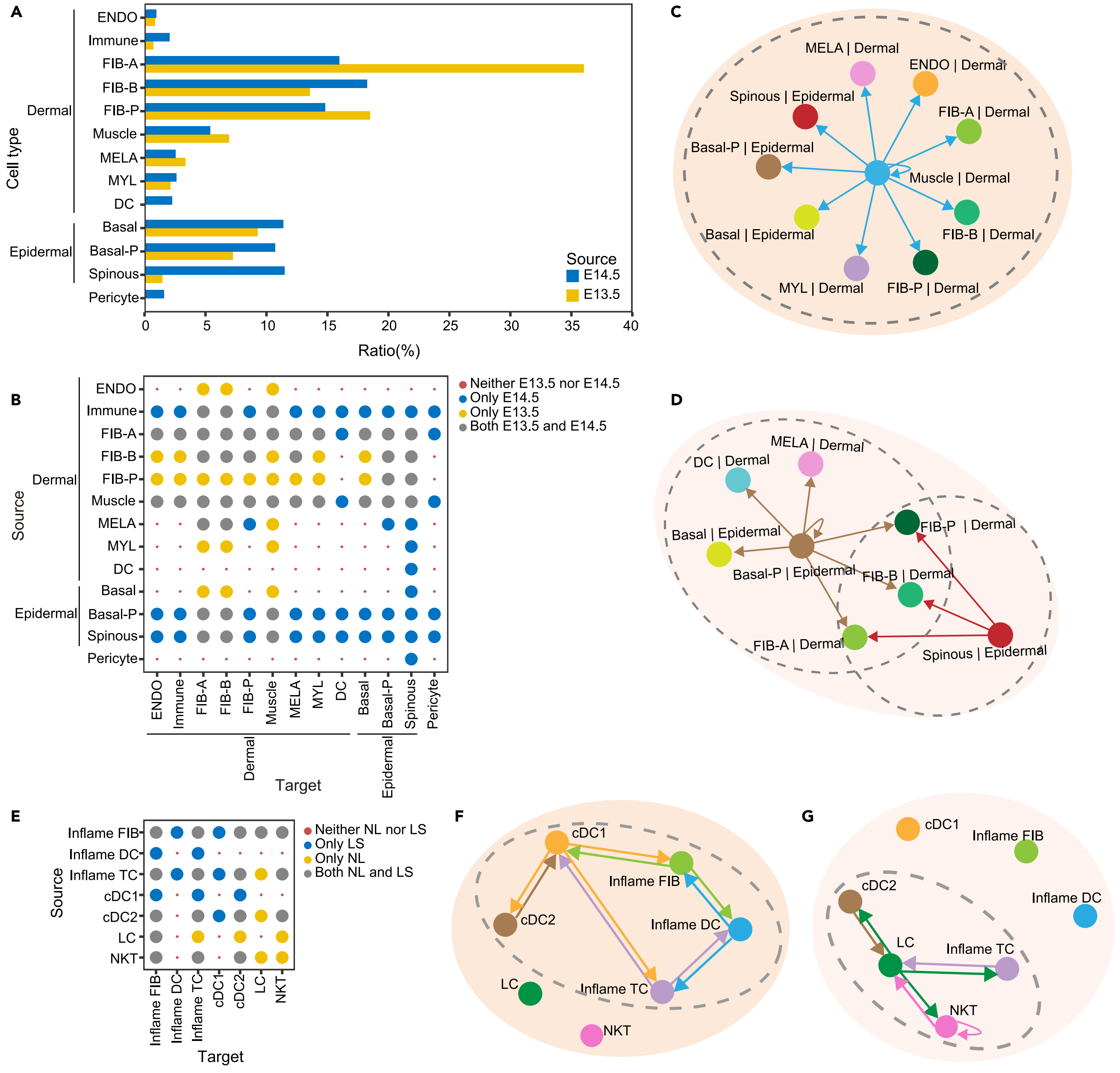

Figure 4. Joint learning at different development stages and in different disease conditions

(A) Comparison of the ratio of different cell types (Table S1) in embryonic day 13.5 (E13.5) and E14.5 mouse skin. FIB-A, fibroblast type A; FIB-B, fibroblast type B; FIB-P, proliferative fibroblasts; DC: dendritic cells; ENDO, endothelial cells; MELA, melanocyte; immune, immune cells; MYL, myeloid cells; muscle, muscle cells; basal, basal cells; basal-P, proliferative basal cells; spinous, spinous epithelial cells.

(B) Comparison of cell-cell communication at the two time points. Significant communications that appeared in both E13.5 and E14.5 are marked with large gray dots. Significant communications that only appeared in E14.5 are marked with large blue dots. Significant communications that only appeared in E13.5 are marked with large yellow dots. Communications that appeared at neither E13.5 nor E14.5 are marked with small red dots.

(C) Visualization of connectivity-summary networks by the top ten communication strength edges on the basis of E13.5. The dashed ovals highlight communication communities centered around different cell types.

(D) Visualization of connectivity-summary networks by the top ten communication strength edges on the basis of E14.5. The dashed ovals highlight communication communities centered around different cell types.

(E) Comparison of cell-cell communication in human lesional (LS) and nonlesional (NL) skin datasets. Significant communications that appeared in both NL and LS are marked with large gray dots. Significant communications that only appeared in LS are marked with large blue dots. Significant communications that only appeared in NL are marked with large yellow dots. Communications that appeared in neither NL nor LS are marked with small red dots. Inflame FIB, inflammatory fibroblasts; cDC, conventional dendritic cell; inflame DC, inflammatory dendritic cell; LC, Langerhans cell; inflame TC, inflammatory T cell; NKT, natural killer T cell.

(F) Visualization of LS-specific communications. The dashed ovals highlight communication communities centered around different cell types.

(G) Visualization of NL-specific communications. The dashed ovals highlight communication communities centered around different cell types.

appeared in both NL skin and LS skin (Figure 4E). These signals suggest that not only inflammatory phenomena exist in LS skin but also that there may be some inflammatory phenomena without obvious inflammatory phenotypes in NL skin. This finding has been mentioned in previous reports that NL skin already shows signs of subclinical inflammation.[93,94] Examining LS-specific communications and NL-specific communications (Figures 4F and 4G), we found that inflammatory cell types (inflame FIB, inflame DC, and inflame TC) were highly active in LS skin, while NL skin was dominated by communications among immune cell types. In fact, inflammatory cells and immune cells are both greatly activated in AD.[93,95–97]

Together, MDIC3 can not only find signal changes in disease conditions but also identify potential signals common to different disease conditions. The joint analyses performed in mice and humans show the ability of MDIC3 to analyze cellular communication across different species.

Comparison of MDIC3 with other cell-cell communication inference methods

We compared MDIC3 with other known methods (i.e., CellChat, CellPhoneDB, and iTALK) to assess their accuracy and effectiveness in identifying intercellular communications in both human and mouse species. We separately applied these four methods to investigate cell-cell communications for five human islet endocrine cell types (alpha, beta, delta, epsilon, and PP cells).[35] MDIC3, CellChat, CellPhoneDB, and iTALK identified 12, 8, 2, and 7 intercellular communications among the five cell types (Figure 5A; Table S2), respectively. We observed that MDIC3 identified 10 particular communications that were not detected by other methods (Figure 5B), iTALK and CellChat separately identified 3 and 4 particular communications for the five cell types, while CellPhoneDB did not identify any particular communication among the five cell types (Figure 5B). This means that MDIC3 can identify more particular communications than other methods. By literature research, we found that 83.33% (10 of 12) of the communications identified by MDIC3 are supported by literature (Figure 5A; Table S2), a ratio significantly higher than those of other methods: CellChat (12.5%), CellPhoneDB (0%), and iTALK (42.86%) (Figure 5A; Table S2). To evaluate the accuracy, we depicted the accuracy of identified cell-cell communications using receiver operating characteristic (ROC) curves for the four methods. We found that the area under the ROC curve (AUC) of MDIC3 was 0.801, which was obviously higher than that of the other three methods: CellChat (0.247), iTALK (0.449), and CellPhoneDB (0.417) (Figure 5C).

Then, we used the human AD scRNA-seq dataset with seven cell types to compare the results of MDIC3 with other tools (CellChat, CellPhoneDB, and iTALK).[30] MDIC3, CellChat, CellPhoneDB, and iTALK separately identified 27, 23, 22, and 15 intercellular communications among the seven cell types (Figure S5A; Table S3). MDIC3 identified 9 particular communications from 27 communications, which were not identified by other methods (Figure S5B). By literature research, we found that 51.85% (14 of 27) of communications identified by MDIC3 can be supported by literature, a ratio higher than the literature-supported communications from CellChat (43.48%) and CellPhoneDB (31.82%) (Figure S5A; Table S3). Additionally, 53.33% (8 of 15) of communications identified by iTALK are supported by literature (Figure S5A), slightly higher than the literature-supported ratio of MDIC3 (Figure S5A). However, MDIC3 identified 27 communications, which is more than the 15 communications from iTALK. Therefore, 14 of 27 communications were supported by literature from MDIC3, and only 8 of 15 communications were supported by the iTALK method (Figure S5A; Table S3). In addition, compared with the ROC curves of the other three methods, MDIC3 achieved the largest AUC (MDIC3, 0.641; CellChat, 0.523; CellPhoneDB, 0.393; iTALK, 0.589) (Figure 5D).

Finally, we selected scRNA-seq mouse data, including nine mouse brain cell types,[66] and compared the cell type communications inferred from MDIC3 and CellChat. We found that MDIC3 and CellChat identified 43 and 34 intercellular communications among the nine cell types, respectively (Figure S5C). MDIC3 and CellChat separately identified 27 and 18 specific communications, respectively, and 16 overlapping communications were identified by both methods (Figure S5D). By literature search, we found that 79.07% (34 of 43) of communications identified by MDIC3 were supported by literature, a ratio significantly higher than the 55.88% (19 of 34) literature-supported ratio from the CellChat method (Figure S5C; Table S4). In addition, MDIC3 achieved a higher AUC than CellChat (MDIC3, 0.711; CellChat, 0.38) (Figure S5E).

Furthermore, we compared the results of MDIC3 with a two-sided self-representation model. The two-sided self-representation model identified 13 intercellular communications among five human islet endocrine cell types (alpha, beta, delta, epsilon, and PP cells),[35] which is slightly higher than MDIC3. However, when both methods have 10 results that could be supported by the literature, only 2 results from MDIC3 had no supported literature, while there were 3 results from the two-sided self-representation model that were not supported by the literature (Figure 5A). Using the human AD scRNA-seq dataset,[30] the two-sided self-representation model identified 25 intercellular communications, with 44% (11 of 25) of these communications being supported by literature (Figure S5A). However, the literature-supported ratio was significantly lower than the 51.85% (14 of 27) achieved by MDIC3 (Figure S5A). Finally, with the scRNA-seq mouse brain dataset,[66] the two-sided self-representation model found only 29 intercellular communication results that could be supported by the literature, with 12 intercellular communication results lacking literature support. In contrast, MDIC3 found 34 intercellular communication results that could be supported by the literature and 9 that lacked literature support (Figure S5C).

In addition to comparisons at the single-cell data level, we also considered the cell type communications enriched in spatial proximity. We used a mouse brain 10X Visium spatial dataset including 1,073 spots. We first deconvoluted the mouse brain 10X Visium spatial dataset using Seurat (https://satijalab.org/seurat/articles/spatial_vignette.html) to predict the proportion of the eight cell types in each spot. Then, the cell type with the largest proportion of each spot was considered as the label of the spot (Figure S6A), and this label was used to investigate potential communications between spots using MDIC3 and CellChat. We assumed that the communications between two cell types with neighboring spots were considered true communication, and the AUCs from MDIC3 and CellChat were 0.572 and 0.432 (Figure S6B; Table S5), respectively. It is worth noting that

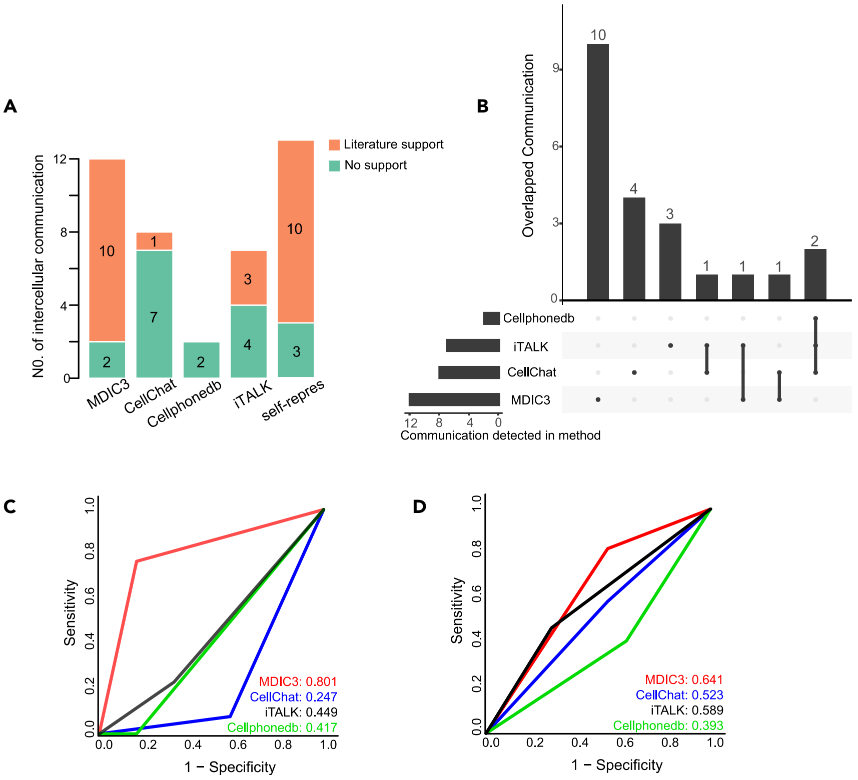

Figure 5. Comparison of the predictive performance of MDIC3 with other methods

(A) Comparison of literature support (Table S2) for the results of intercellular communication among endocrine cells in the human pancreatic islet dataset predicted by five methods (MDIC3, CellChat, CellPhoneDB, iTALK, and the two-sided self-representation model). The horizontal ordinate represents the five methods, and the vertical ordinate is the number of intercellular communications from the five methods. The orange bar represents the number of intercellular communications with literature support, and the green bar represents the number of intercellular communications without literature support from the PubMed database (https://pubmed.ncbi.nlm.nih.gov).

(B) UpSetR plot of predicted cellular communication results among endocrine cells on the human pancreatic islet dataset from four methods (MDIC3, CellChat, CellPhoneDB, and iTALK). The horizontal bar graph in the bottom left represents the total number of cell-cell communications detected by different methods. Dots are used to indicate the corresponding method on the left. If cell-cell communications were detected only by one method, only one gray dot was darkened, and the number of detected cell-cell communications is shown in the bar graph form above. The intersection of the cell-cell communication results obtained by different methods is shown by multiple black dots connected by lines, and the number of the intersection of intercellular communication results is represented in the bar graph form above.

(C) ROC curves of the results of four methods (MDIC3, CellChat, CellPhoneDB, and iTALK) on intercellular communications with literature support (Table S2) among endocrine cells in the human pancreatic islet dataset.

(D) ROC curves of the four methods (MDIC3, CellChat, CellPhoneDB, and iTALK) on intercellular communications with literature support (Table S3) on the human lesional skin dataset.

some of the communications judged to be false positives may be due to multiple cell types mixing in spatially proximate spots. Therefore, these existing methods may not be suitable for the evaluation of cell-cell communications for spatial datasets.

Investigating cell-cell communication in 4 hpf zebrafish embryos

To better demonstrate the applicability to any species of MDIC3, we applied MDIC3 to zebrafish species. Cell-cell communication may be accomplished through contact among different cell types or mediated by proteins and may occur early during cell type determination.[98] We used the 4 hpf (hours post fertilization) zebrafish embryo scRNA-seq dataset[99] to explore cell-cell communications during early zebrafish embryonic development. There are four cell types detected in the 4 hpf zebrafish embryo, namely, epiblast, mesoderm, germline, and enveloping layer (EVL).

Epiblast cells play an important role during early zebrafish embryonic development, with the ability to generate epidermal and neural ectoderm.[100] MDIC3 found communication between epiblasts and mesoderm, which is consistent with a previous report.[98] The interactions between the initial cells of the mesoderm and epiblast determine the neuroectoderm and promote the development of the neuroectoderm.[98] MDIC3 also found that the significant cell-cell communications at the 4 hpf zebrafish embryonic stage were centered around epiblast cells (Figure S7). Bidirectional paracrine signaling exists among the epiblast and mesoderm, germline, and EVL. This phenomenon of cell-cell communication centered around epiblast cells may be related to the formation of intercellular bridges during the early stages of zebrafish embryonic development.[101] Caneparo et al.[101] found that intercellular bridges join a significant fraction of epiblast cells in the zebrafish embryo, reaching several cell diameters in length and spanning across different regions of the developing embryos, and the rate of transfer of membrane proteins along the intercellular bridges is fast enough to mediate cell-cell communication during gastrulation and neurulation. In addition, the communication between EVL and epiblasts may be related to the spatial location of the two different cell types. EVL is the outer epithelial monolayer that protects the embryo and surrounds the entire embryo, including epiblast cells.[98,101] This proximity in spatial location is probably the main reason for the existence of communication between EVL and epiblast cells.

Overall, we applied the MDIC3 algorithm to organisms beyond mice and humans, demonstrating that the MDIC3 algorithm has a wide range of applications for different biological species.

Identifying key L-R pairs from cell-cell communication

To further explain the mechanisms of cell-cell communications predicted by MDIC3, we identified the specific L-R pairs that participated in corresponding cell-cell communications.

We identified L-R pairs involved in communication between the inflammatory FIB and the inflammatory DC cell type in the human AD LS skin cell dataset[30] using MDIC3 and CellChat. MDIC3 identified 2 L-R pairs, while CellChat identified 3 L-R pairs, and the results of MDIC3 are all included in the results of CellChat (Table S6). *CCL19-CCR7* is the L-R pair involved in communication between the inflammatory FIB and the inflammatory DC, identified by immunofluorescence experiments in the original human LS skin cell dataset literature.[30] In addition, *CXCL12-CXCR4* is the L-R pair that plays a role in inflammatory diseases of the skin, including AD,[102] and *CXCL12-CXCR4* signaling is often associated with inflammatory cells.[103] Regarding the L-R pair *MIF*-(*CD74*+*CXCR4*), there are no reports in the literature on its involvement in the communication process of inflame FIB and inflame DC. In addition, as the ligand *MIF* is significantly expressed in more than half of the cell types, it may not be a ligand gene with cell type specificity. Therefore, *MIF*-(*CD74*+*CXCR4*) may not be a specific L-R pair for communication between inflame FIB and inflame DC.

We also identified the L-R pairs significantly involved in different cell-cell communications under the E14.5 mouse skin dataset[56] using MDIC3 and CellChat. We explored L-R pairs involved in cellular communication between the epidermal cell type basal-P and the dermal cell type FIB-B. MDIC3 identified 17 L-R pairs, while CellChat identified 8 L-R pairs. Four L-R pairs were identified to overlap between the two methods (Table S7). We found that 12 of the 13 pairs from MDIC3-specific results were WNT signaling LR pairs, which is consistent with literature reports that epidermal cells control and induce hair follicle development through WNT signaling to coordinate signaling crosstalk between the epidermis and dermis.[57] In addition, WNT signaling has also been reported to be essential for fibroblast proliferation in hair follicle development.[58] The 4 pairs of CellChat-specific results were dominated by the ligands *Ptn* and *Mdk*; however, the genes *Ptn* and *Mdk* were significantly expressed in more than half of the cell types, and thus, they may not be ligand genes with cell type specificity. Furthermore, we explored L-R pairs involved in autocrine communication of the epidermal cell type basal. MDIC3 identified 34 L-R pairs, CellChat identified 9 L-R pairs, and there was no overlapping pair between the results from MDIC3 and CellChat and the results (Table S8). We found that 24 of the 34 L-R pairs resulting from MDIC3 were WNT signaling L-R pairs, which may be related to the experimentally discovered involvement of the WNT signaling pathway in autocrine secretion in the basal layer of the epidermis.[104] Additionally, biological experiments have shown that WNT autocrine signaling from epidermal cells promotes induced hair follicle development.[58] In contrast, 8 of the 9 pairs of L-R results from CellChat resulted in ligand signaling by the genes *Ptn* and *Mdk* (Table S8), which are significantly expressed in more than half of the cell types and therefore may not be ligand genes with cell type specificity.

The main purpose of applying cell-cell communication analysis is to explain cell functions through L-R pairs. By comparison with CellChat, MDIC3 can not only investigate cell-cell communication without prior knowledge but also identify important and specific L-R pairs in cellular communication.

DISCUSSION

In prior research, the single-cell gene expression matrix was decomposed by the one-sided self-representation model to reveal gene regulatory information. However, different variants of the one-sided self-representation model show that single-cell gene expression data not only contain regulatory information among genes but also contain regulatory information among cells. In fact, the expression of a gene in a cell is typically controlled by two pathways. One path is that the gene can be directly regulated by other upstream genes or factors in the same cell. Another path is that the cell can be affected or mediated by other cells in the same tissue or organ, and the gene expression in the cell can be indirectly regulated by other cells. Therefore, a single-cell expression matrix should contain both regulatory information among genes and crosstalk information among cells. Matrix decomposition or factorization can be used to reveal the discipline of regulation or crosstalk among cells from the expression matrix.

MDIC3 is a new method to investigate cell-cell communication by matrix decomposition with a GRN. These are three main advantages of MDIC3 compared with other existing methods. First, the MDIC3 method is based on matrix decomposition for inferring cell-cell communications and does not require any prior biological information, such as the L-R database. Consequently, MDIC3 is not affected by the quality of existing L-R information. In contrast, other methods, such as CellChat, CellPhoneDB, and iTALK, investigate cell-cell communications on the basis of L-R coexpression, making the final results more sensitive to the completeness and accuracy of L-R information (Table S9). Second, MDIC3 does not rely on prior information, allowing it to be applied to any species. However, most other methods investigate cell-cell communications on the basis of L-R information, limiting their applicability to species for which L-R information is available (Table S9). Existing L-R studies are mainly focused on humans, and the collection and establishment of L-R bioinformatics databases are also mainly focused on humans. Although there are a few L-R databases for other species, such as the CellChatDB L-R database including mice, establishing L-R databases for all species is challenging. Therefore, most existing methods are limited by the L-R information collection of species. Third, MDIC3 infers that cell-cell communications do not depend on specific L-R signaling and at the global cell level. In contrast, CellChat, CellPhoneDB, and iTALK investigate cell-cell communications at the local cell level on the basis of specific L-Rs or pathways (Table S9). MDIC3 is in line with the biological nature of cell-cell communication, as cell-cell communication is the result of interactions between individual cells via multiple L-Rs and pathways.

Current studies on intercellular communication mainly rely on gene coexpression of L-R pairs in different cells.[1] However, building a complete and accurate L-R database is a challenging task. To date, there are still many L-R pairs waiting to be discovered, including some receptors lacking known ligands, such as *DR6* (*TNFRSF21*), *RELT*, *TROY*, and *NGFR* from the *TNF* receptor family,[105,106] and some ligands without receptors, such as *IL17D*.[105,107] In addition, with the development of intercellular communication research, more and more information other than L-R pairs has been taken into account, such as the secretion

of metabolites.[108,109] Therefore, existing methods are unable to investigate cell-cell communications with high completeness and accuracy until a comprehensive L-R database is established. To our knowledge, MDIC3 is a rare cell-cell communication algorithm that is not limited to the dependence on biological prior information of specific species. The output of MDIC3 provides a global view of cellular crosstalk at the cell level and can be interpreted as all the cellular communication pairs across all known and unknown L-R pairs, rather than focusing on any specific L-R pairs. The cell-cell communications from MDIC3 avoid the effect of unknown L-R pairs and are in line with the biological nature. Similarly, GraphFP,[110] like MDIC3, does not rely on L-R databases in its cell-cell interaction prediction process, and its results also provide an overall perspective. However, GraphFP is designed for predicting cell-cell interactions on the basis of a large amount of dynamic time-series data, making it more suitable for the analysis of time-series data with multiple time points and may be more advantageous when exploring changes in cell-cell interactions at different time points. More details about the relationship between the two types of approaches can be found in Note S1.

The successful performance of MDIC3 is attributed to three aspects. First, MDIC3 uses GRNs to extract gene regulatory information. The GRN can usually be inferred by gene expression data without prior knowledge, but the accuracy of the GRN can affect the results of cell-cell communication for MDIC3. Therefore, an efficient and accurate method to investigate GRNs from gene expression data is an important tool for this study. Here, we used the GNIPLR[111] algorithm (gene network inference on the basis of projection and lagged regression) to investigate GRNs with high accuracy. In fact, GRN can cover most of the known and unknown gene regulatory information, among which the secretion of L-R and the activity of downstream factors are also included in the results of gene regulation. In addition, it should be noted that MDIC3 is not limited by GNIPLR, and any tool that infers regulatory networks can be used for MDIC3, and the quality of the regulatory network may affect communication inference results. Second, it is worth noting that most existing methods analyze the interaction among cell types, but it cannot be ignored that the life activities of multicellular organisms depend on the mutual communication between a large number of individual cells. Communications between cell types inferred by MDIC3 are based on the global integration of the communication network between individual cells. This derivation in a more detailed dimension greatly enhances the plausibility of the MDIC3 results and provides an effective tool for studying the interactions between individual cells. Considering the high dropout effect in each single cell, we also explored whether different gene coverage affects the prediction results of MDIC3 (Note S2). We found that the MDIC3 algorithm is stable and robust, and the results from different gene coverages are also acceptable (Table S10). Finally, after decomposing the original gene expression data, the obtained singular matrix extracts the association information between genes and cells, which is an important hub connecting the relationship between genes and cells. Although there may be some missing information in the right submatrix when the number of cells is greater than the number of genes, we believe that adjusting according to the method of cell sampling can be considered a reasonable processing method.

For more benchmarking approaches, we calculated whether the global communication results of MDIC3 were significantly correlated with the expression of all L-R pairs from different L-R databases of different algorithms (Note S3). By applying this approach to the human LS skin dataset, we observed a significant correlation between the MDIC3 results and the overall expression of L-R pairs (Note S3). We also benchmarked how well the matrix decomposition actually works by calculating the mean squared error (MSE) between the reconstructed matrix and the original single-cell gene expression matrix (Note S4). Our findings indicated that the MSEs for the human LN dataset, the E14.5 mouse skin dataset, and the mouse brain dataset were 0.22, 0.25, and 0.24, respectively (Note S4).

MDIC3 is species-independent for analyzing cell-cell communication. To validate its effectiveness, we applied MDIC3 to five recently published scRNA-seq datasets[13,30,35,56,66] from two species, human and mouse. As the communication network of individual cells is difficult to verify, we therefore demonstrated MDIC3's ability to investigate communication networks among cell types. Ultimately, we found many inferences that were largely consistent with known biological conclusions. Furthermore, we compared the inference ability of MDIC3 with other existing methods in a unified manner and found that some known biological communication conclusions can be inferred by MDIC3 but do not appear in the results of other methods. Then, we also found some known L-R pairs that were not included in the existing L-R database. This observation not only underscores MDIC3's ability to investigate more comprehensive results compared with other methods but also reveals the challenges associated with constructing and updating L-R databases. Furthermore, we evaluated the performance of MDIC3 using a spatial dataset from mice. We also extended the application of MDIC3 to zebrafish, demonstrating its potential for studying cell communication in diverse species. We recorded the computational cost of MDIC3 in both simulation datasets and real datasets (Note S5), and we considered that the computational costs of MDIC3 are acceptable for a personal computer (Table S11 and Table S12).

EXPERIMENTAL PROCEDURES

Resource availability

Lead contact

The lead contact for questions about this paper is Xiaoping Liu, who can be reached at xpliu@ucas.ac.cn.

Materials availability

No unique materials were generated from this study.

Data and code availability

This paper analyzes existing, publicly available data. The human LN dataset can be accessed via ImmPort repository (https://www.immport.org/shared/home): SDY997.[13] The human LS skin dataset can be obtained via Gene Expression Omnibus (GEO; https://www.ncbi.nlm.nih.gov/geo/) database accession number GEO: GSE147424.[30] The human NL skin dataset can be obtained via GEO: GSE147424.[30] The human islet dataset can be downloaded via GEO: GSE116753.[35] The E14.5 mouse skin dataset can be obtained via GEO: GSE122043.[56] The E13.5 mouse skin dataset can be obtained via GEO: GSE122043.[56] The mouse brain dataset can be obtained via GEO: GSE60361.[66] The 04hpf zebrafish embryo dataset can be obtained via GEO: GSM3067189.[99] The mouse brain 10X Visium spatial dataset can be obtained from the 10X Genomics website: https://www.10xgenomics.com/resources/datasets/mouse-brain-serial-section-1-sagittal-anterior-1-standard-1-0-0. MDIC3 is publicly available as a Python package. Source code and tutorials

have been deposited at the GitHub repository (https://github.com/LYxiaotai/MDIC3). All original code has been deposited at figshare (https://doi.org/10.6084/m9.figshare.24486046.v1) and is publicly available as of the date of publication.[112] Any additional information required to reanalyze the data reported in this paper is available from the lead contact upon request.

Methodology

Examination of the cell-cell communication network using MDIC3

Given a single-cell gene expression matrix A with size $m \times n$ as input, the matrix A contains m genes and n cells. Previous studies[113–116] have focused on decomposing the matrix A by using the one-sided self-representation model to investigate gene regulatory relationships. The formula is as follows:

$$A = ZA \quad \text{(Equation 1)}$$

where matrix Z is of size $m \times m$.

Equation 1 is based on single-cell gene expression data, and the matrix Z with size $m \times m$ represents the GRN adjacency matrix among the m genes, which means that the single-cell gene expression data itself contain gene regulation information.

Similarly, the matrix A can also be decomposed by another one-sided self-representation model:

$$A = AX \quad \text{(Equation 2)}$$

where the matrix X with size $n \times n$ in Equation 2 is able to represent the regulatory relationship among the n cells. Considering that cell-cell communication can be regarded as regulatory among cells, the matrix X may represent the adjacency matrix of the cell-cell communication network, which suggests that the single-cell gene expression data itself also contain cell regulation information.

However, using Equation 2 directly may be limited by the requirement for time-series data.

Notably, by combining the Equation 1 and Equation 2, single-cell gene expression data not only contain regulatory information among genes but also contain regulatory information among cells. Using matrix decomposition can reveal the discipline of regulation from the expression matrix. Therefore, we combined Equation 1 and Equation 2 to obtain the two-sided self-representation model for factorizing the single-cell gene expression matrix A:

$$A = ZAX \quad \text{(Equation 3)}$$

However, Equation 3 uses the original expression matrix as the middle submatrix, and the original single-cell expression matrix may always contain other noise.

Here, we propose a matrix decomposition-based method called MDIC3 to investigate the cell-cell communication network only from a single-cell gene expression matrix A without relying on any prior knowledge, such as L-R pairs. Different from Equation 2, the input single-cell gene expression matrix A in the MDIC3 model is not constrained by time-series data. Different from Equation 3, MDIC3 uses the singular matrix to better extract the information containing the relationships between genes and cells.

The cell-cell communication network can be described as a weighted directed graph that connects individual cells (Figure 1). The cell-cell relationships in the weighted directed graph consist of two parts: communication direction and communication strength. Specifically, an arrow from point i to point j in the weighted directed graph represents that the communication signal direction is sent from cell i and received by cell j, and the weight of the arrow from point i to point j represents the communication strength when the signal is sent from cell i and received by cell j.

Cell-cell communication represents a special type of regulatory relationship among cells. MDIC3 aims to investigate the regulatory relationship among cells on the basis of the regulatory relationships among genes and considers that cell-cell communication information can be derived from the single-cell gene expression profile itself. MDIC3 factorizes the matrix A into three matrices R, Σ, and W (Figure 1):

$$A = R\Sigma W \quad \text{(Equation 4)}$$

First, the matrix R is the left submatrix with a size of $m \times m$, m denotes the number of genes in single-cell expression matrix A, and this matrix R denotes the relationship among the m genes. MDIC3 uses the GRN adjacency matrix of matrix A as the left submatrix R in Equation 4.

Then, the matrix Σ is the middle submatrix with dimension $m \times n$, and this matrix Σ connects the relationships between genes and cells. Considering that the singular matrix has the ability to extract the features of the matrix A, MDIC3 uses the singular value matrix of matrix A as the middle submatrix Σ in Equation 4. The singular matrix corresponding to matrix A can be obtained by decomposing matrix A using the singular value decomposition method.

Finally, the matrix W is the right submatrix with a size of $n \times n$, n denotes the number of cells of matrix A, and this matrix W reflects the relationship among the n cells.

On the basis of the adjacency matrix R of the GRN, the single-cell expression matrix A, and the singular value matrix Σ, MDIC3 can obtain the cell-cell communication network by solving the right submatrix W of Equation 4:

$$W = (R\Sigma)^{-1}A \quad \text{(Equation 5)}$$

Here, Equation 5 is only applicable when the number of cells and the number of genes are equal and matrix $R\Sigma$ is invertible. However, in most cases, the number of genes and the number of cells are not equal, which means that $m \neq n$ and $R\Sigma$ is a nonsquare matrix. In addition, even if $R\Sigma$ is a square matrix, its inverse matrix does not always exist.

Therefore, we adopt the pseudoinverse matrix $(R\Sigma)^{+}$ to solve the matrix W:

$$W = (R\Sigma)^{+}A \quad \text{(Equation 6)}$$

Each item in W indicates how individual cells send to or receive from signals of others, and matrix W is the cell-cell communications inferred from Equation 6.

To be more specific, item W_{ij} represents the value in the ith row and the jth column in matrix W, when $i = j$, W_{ii} represents the autoregulation of cell i, and when $i \neq j$, W_{ij} represents the signals between cells i and j. The sign of W_{ij} represents the communication direction between the cell i and j, and $|W_{ij}|$ denotes the communication strength between the ith and jth cell. That is, if $W_{ij} \geq 0$, the communication signal is sent from cell i to cell j; if $W_{ij} < 0$, the communication signal is sent from cell j to cell i. The final communication between cell i and cell j is determined jointly by W_{ij} and W_{ji}.

In summary, MDIC3 can investigate intercellular relationships on the basis of intergenic relationships from single-cell expression profiles, so we can uncover the network of cell communication among individual cells without using prior knowledge such as L-R databases.

Construction of GRNs (optional)

The GRN is a type of biological network that reflects the regulatory relationships among genes.[117] The MDIC3 method uncovers the intercellular relationships among cells on the basis of the GRN, which can be determined from the single-cell expression profiles. Here, we use the GNIPLR[111] algorithm, taking the single-cell gene expression matrix A with size $m \times n$ as input to investigate its GRN (Note S6). The GNIPLR algorithm projected gene data twice using the LASSO projection algorithm and the linear projection approximation to produce a linear and monotonous pseudotime series and then determined the direction of regulation in combination with lagged regression analyses.[111] Each node of the GRN represents a gene, and each edge from gene i to gene j indicates that gene i can regulate gene j in the network (Figure 1). The GRN can be expressed as an adjacency matrix R with size $m \times m$, where R_{ij} is the ith row and jth column in R and the value of R_{ij} represents the regulatory strength from gene i to gene j (Figure 1).

However, it should be noted that any tool capable of inferring GRNs can potentially replace the GNIPLR algorithm. GRNs with varying quality can impact the inference results of cell-cell communication from MDIC3, yet most existing GRN tools yield similar results regarding cell-cell communication from MDIC3, albeit with subtle differences.

We used two regulatory network inference tools (GENIE3[118] and GRNBoost[119]) to replace the GRN from GNIPLR and compared the cellular network from MDIC3 under the three tools for regulatory network inference tools (GNIPLR, GENIE3, and GRNBoost) in the human LS skin cell dataset and the human islet

endocrine cell dataset. The results of the cell-cell communication network from the three regulatory networks show differences, albeit subtle, among the three GRNs (Table S13). MDIC3 performs better when using the GRN from the GNIPLR algorithm. We believe that MDIC3 is sufficiently robust, and users can also provide their own GRN results for use in MDIC3 inference.

The method of identifying L-Rs from cell-cell communication

We extracted L-R pairs from cell-cell communication and validated the importance of the L-R pairs in the literature. Considering that the CellChatDB L-R database contains both human and mouse L-R information, we obtained human and mouse L-R genes from CellChatDB.

We used a simple method to identify key L-R pairs from cell-cell communication. We can let the gene set contained in the gene expression data be G_1 and the gene set contained in the L-R information extracted from CellChatDB be G_2. We first match the gene set G_1 and the gene set G_2 and then obtain the gene set G_3, $G_3 = G_1 \cap G_2$.

Then, genes in the gene set G_3 were further screened to identify genes with cell type expression specificity: if a gene is significantly expressed in more than half of the cell types, we consider it a universally expressed gene and conclude that it does not possess cell type-specific expression. Therefore, we removed the gene from the gene set G_3. Through these filtering steps, we obtain a new gene set G_4.

Next, on the basis of the gene set G_4, we aim to identify L-R pairs involved in the communication from cell type A (containing m_1 cells) to cell type B (containing m_2 cells) in the following steps:

(1) For a specific cell type, genes whose zero expression values are in more than 70% of cells in a cell type were removed.
(2) For a pair of L-R gene pairs L-R, the ligand gene expression levels of the m_1 cells contained in cell type A were sorted in descending order, and the sorted result was recorded as $L(L_1, L_2 \cdots, L_{m_1})$. The receptor gene expression levels of the m_2 cells contained in cell type B were sorted in descending order, and the sorted result were recorded as $2R(R_1, R_2 \cdots, R_m)$.
(3) The correlation between the expression of ligand gene L_i and the expression of receptor gene R_j was calculated. If $m_1 < m_2$, Pearson's correlation coefficient and significant p value between $L(L_1, L_2 \cdots, L_{m_1})$ and $R(R_1, R_2 \cdots, R_{m_1})$ were calculated. If $m_1 > m_2$, Pearson's correlation coefficient and significant p value between $L(L_1, L_2 \cdots, L_{m_2})$ and $R(R_1, R_2 \cdots, R_{m_2})$ were calculated.
(4) The Bonferroni correction method was used to adjust the p values obtained in (3) for significant receptor pairs. The L-R pairs with corrected p values less than 0.01 were considered significant.

Comparison with other common methods

L-R pairs are used by existing methods as input, but unlike existing methods, MDIC3 only uses L-R pairs for validation and comparison. The analysis of L-R pairs covered in the paper is mainly used for validation of the results and comparison with other tools. To compare with the other three existing tools, we selected L-R pairs that have been reported to be relevant to the biological context of the dataset. We used three human datasets, i.e., the human LN dataset,[13] human LS skin dataset[30] and human islet dataset[35] (Table S1), to compare the results of MDIC3 with three other well-known methods (CellChat,[4] CellPhoneDB,[7] and iTALK[8]). As only CellChat provided the mouse L-R database, we only compared the results of MDIC3 with CellChat for two scRNA-seq mouse datasets: the E14.5 mouse skin dataset[56] and the mouse brain dataset[66] (Table S1). Furthermore, we compared the results of MDIC3 with CellChat for a mouse brain 10X Visium spatial dataset.

We use the AUC to evaluate the accuracy of MDIC3 and other tools. First, we aggregated the communication results from the three other methods (CellChat, CellPhoneDB, and iTALK). As the inferred communications of most existing methods are limited to specific L-R pairs, most of them only output the communication strength on the basis of specific L-R pairs, except CellChat. Accordingly, we calculated each cell-cell communication by aggregating all the communication probabilities under all L-R pairs between the two cells, referring to the process of CellChat. The three methods were used by their default parameters and the L-R database. More details for obtaining the aggregated communication results from other methods can be found in Note S7. Then, we consider the mean value of all cell type communication results as the threshold and calculate the threshold for each method. On the basis of the thresholds of each method, the communication results can be divided into two groups: communications above the threshold value were placed into the communication group (marked as "1" in Tables S2–S4), while those below the threshold were categorized as noncommunication (marked as "0" in Tables S2–S4). We then considered cellular communications supported by the literature as positive samples and those not supported by the literature as negative samples, treating the results as ground truth. Finally, the true positive rate and false-positive rate were calculated by comparison with the ground truth. Therefore, the performance evaluation of the communication results became a dichotomous problem and was evaluated using the AUC. The rules for selecting the literature are as follows: (1) the interaction between two cell types had been reported in the literature, and (2) the existing L-R had been mentioned in the corresponding cells.

We compared the AUC of MDIC3 with CellPhoneDB and iTALK by using the human LS skin scRNA-seq dataset[30] and human islet scRNA-seq dataset.[35] Additionally, we compared the AUC of MDIC3 with CellChat using the human LS skin scRNA-seq dataset,[30] human islet scRNA-seq dataset,[35] and mouse brain scRNA-seq dataset.[66] Furthermore, we compared the AUC of MDIC3 with the two-sided self-representation model using both human and mouse scRNA-seq datasets (Note S8).

Moreover, we also compared the AUC of MDIC3 with CellChat using a mouse brain 10X Visium spatial dataset. We assumed that communication exists only between spatially proximate cell types, where two neighboring spots with main cell types are considered to communicate with each other. If cells are not spatially proximate but are predicted to communicate, that would be a false positive. If cells are spatially proximate but not predicted to communicate, that would be a false negative.

SUPPLEMENTAL INFORMATION

Supplemental information can be found online at https://doi.org/10.1016/j.patter.2023.100911.

ACKNOWLEDGMENTS

This work was supported by the National Key Research and Development Program of China (grant 2022YFA1004800), the Zhejiang Provincial Natural Science Foundation of China (grant LZ22C060001 and LY19C100001), and the research funds of Hangzhou Institute for advanced study, UCAS (grants 2022ZZ01013 and 2022ZZ01016).

AUTHOR CONTRIBUTIONS

X.L., Y.L., and X.C. conceived the project. Y.L., X.L., and Y.Z. developed and implemented the computational approach. X.L. and X.C. supervised the work. Y.L. and X.L. wrote the manuscript.

DECLARATION OF INTERESTS

The authors declare no competing interests.

Received: December 12, 2022
Revised: May 31, 2023
Accepted: December 8, 2023
Published: January 11, 2024

REFERENCES

1. Armingol, E., Officer, A., Harismendy, O., and Lewis, N.E. (2021). Deciphering cell-cell interactions and communication from gene expression. Nat. Rev. Genet. *22*, 71–88.
2. Ramilowski, J.A., Goldberg, T., Harshbarger, J., Kloppmann, E., Lizio, M., Satagopam, V.P., Itoh, M., Kawaji, H., Carninci, P., Rost, B., and Forrest, A.R.R. (2015). A draft network of ligand-receptor-mediated multicellular signalling in human. Nat. Commun. *6*, 7866.

CellPress
OPEN ACCESS

3. Zhang, Y., Liu, T., Hu, X., Wang, M., Wang, J., Zou, B., Tan, P., Cui, T., Dou, Y., Ning, L., et al. (2021). CellCall: integrating paired ligand-receptor and transcription factor activities for cell-cell communication. Nucleic Acids Res. *49*, 8520–8534.

4. Jin, S., Guerrero-Juarez, C.F., Zhang, L., Chang, I., Ramos, R., Kuan, C.H., Myung, P., Plikus, M.V., and Nie, Q. (2021). Inference and analysis of cell-cell communication using CellChat. Nat. Commun. *12*, 1088.

5. Jin, S., MacLean, A.L., Peng, T., and Nie, Q. (2018). scEpath: energy landscape-based inference of transition probabilities and cellular trajectories from single-cell transcriptomic data. Bioinformatics *34*, 2077–2086.

6. Yuan, G.C., Cai, L., Elowitz, M., Enver, T., Fan, G., Guo, G., Irizarry, R., Kharchenko, P., Kim, J., Orkin, S., et al. (2017). Challenges and emerging directions in single-cell analysis. Genome Biol. *18*, 84.

7. Vento-Tormo, R., Efremova, M., Botting, R.A., Turco, M.Y., Vento-Tormo, M., Meyer, K.B., Park, J.E., Stephenson, E., Polański, K., Goncalves, A., et al. (2018). Single-cell reconstruction of the early maternal-fetal interface in humans. Nature *563*, 347–353.

8. Yuanxin, W., Ruiping, W., Shaojun, Z., Shumei, S., Changying, J., Guangchun, H., Michael, W., Jaffer, A., Andy, F., and Linghua, W. (2019). iTALK: an R Package to Characterize and Illustrate Intercellular Communication. Preprint at bioRxiv.

9. Browaeys, R., Saelens, W., and Saeys, Y. (2020). NicheNet: modeling intercellular communication by linking ligands to target genes. Nat. Methods *17*, 159–162.

10. Wang, S., Karikomi, M., MacLean, A.L., and Nie, Q. (2019). Cell lineage and communication network inference via optimization for single-cell transcriptomics. Nucleic Acids Res. *47*, e66.

11. Cillo, A.R., Kürten, C.H.L., Tabib, T., Qi, Z., Onkar, S., Wang, T., Liu, A., Duvvuri, U., Kim, S., Soose, R.J., et al. (2020). Immune Landscape of Viral- and Carcinogen-Driven Head and Neck Cancer. Immunity *52*, 183–199.e9.

12. Cohen, M., Giladi, A., Gorki, A.D., Solodkin, D.G., Zada, M., Hladik, A., Miklosi, A., Salame, T.M., Halpern, K.B., David, E., et al. (2018). Lung Single-Cell Signaling Interaction Map Reveals Basophil Role in Macrophage Imprinting. Cell *175*, 1031–1044.e18.

13. Arazi, A., Rao, D.A., Berthier, C.C., Davidson, A., Liu, Y., Hoover, P.J., Chicoine, A., Eisenhaure, T.M., Jonsson, A.H., Li, S., et al. (2019). The immune cell landscape in kidneys of patients with lupus nephritis (vol 20, pg 902, 2019). Nat. Immunol. *20*, 1404.

14. Costenbader, K.H., Desai, A., Alarcón, G.S., Hiraki, L.T., Shaykevich, T., Brookhart, M.A., Massarotti, E., Lu, B., Solomon, D.H., and Winkelmayer, W.C. (2011). Trends in the Incidence, Demographics, and Outcomes of End-Stage Renal Disease Due to Lupus Nephritis in the US From 1995 to 2006. Arthritis Rheum. *63*, 1681–1688.

15. Tektonidou, M.G., Dasgupta, A., and Ward, M.M. (2016). Risk of End-Stage Renal Disease in Patients With Lupus Nephritis, 1971-2015 A Systematic Review and Bayesian Meta-Analysis. Arthritis Rheumatol. *68*, 1432–1441.

16. Thanou, A., and Merrill, J.T. (2014). Treatment of systemic lupus erythematosus: new therapeutic avenues and blind alleys. Nat. Rev. Rheumatol. *10*, 23–34.

17. Banchereau, R., Hong, S., Cantarel, B., Baldwin, N., Baisch, J., Edens, M., Cepika, A.M., Acs, P., Turner, J., Anguiano, E., et al. (2016). Personalized Immunomonitoring Uncovers Molecular Networks that Stratify Lupus Patients (vol 165, pg 551, 2016). Cell *165*, 1548–1550.

18. Klocke, J., Kopetschke, K., Grießbach, A.S., Langhans, V., Humrich, J.Y., Biesen, R., Dragun, D., Radbruch, A., Burmester, G.R., Riemekasten, G., and Enghard, P. (2017). Mapping urinary chemokines in human lupus nephritis: Potentially redundant pathways recruit CD4(+) and CD8(+) T cells and macrophages. Eur. J. Immunol. *47*, 180–192.

19. Kuroiwa, T., and Lee, E.G. (1998). Cellular interactions in the pathogenesis of lupus nephritis: The role of T cells and macrophages in the amplification of the inflammatory process in the kidney. Lupus *7*, 597–603.

20. Kulkarni, O., Pawar, R.D., Purschke, W., Eulberg, D., Selve, N., Buchner, K., Ninichuk, V., Segerer, S., Vielhauer, V., Klussmann, S., and Anders, H.J. (2007). Spiegelmer inhibition of CCL2/MCP-1 ameliorates lupus nephritis in MRL-(Fas)lpr mice. J. Am. Soc. Nephrol. *18*, 2350–2358.

21. von Vietinghoff, S., and Kurts, C. (2021). Regulation and function of CX3CR1 and its ligand CX3CL1 in kidney disease. Cell Tissue Res. *385*, 335–344.

22. Low, S., Wu, H., Jerath, K., Tibolla, A., Fogal, B., Conrad, R., MacDougall, M., Kerr, S., Berger, V., Dave, R., et al. (2020). VHH antibody targeting the chemokine receptor CX3CR1 inhibits progression of atherosclerosis. mAbs *12*, 1709322.

23. Cockwell, P., Calderwood, J.W., Brooks, C.J., Chakravorty, S.J., and Savage, C.O.S. (2002). Chemoattraction of T cells expressing CCR5, CXCR3 and CX3CR1 by proximal tubular epithelial cell chemokines. Nephrol. Dial. Transplant. *17*, 734–744.

24. Tanaka, Y., Hoshino-Negishi, K., Kuboi, Y., Tago, F., Yasuda, N., and Imai, T. (2020). Emerging Role of Fractalkine in the Treatment of Rheumatic Diseases. ImmunoTargets Ther. *9*, 241–253.

25. Perl, A., Fernandez, D.R., Telarico, T., Doherty, E., Francis, L., and Phillips, P.E. (2009). T-cell and B-cell signaling biomarkers and treatment targets in lupus. Curr. Opin. Rheumatol. *21*, 454–464.

26. Ramanujam, M., Steffgen, J., Visvanathan, S., Mohan, C., Fine, J.S., and Putterman, C. (2020). Phoenix from the flames: Rediscovering the role of the CD40-CD40L pathway in systemic lupus erythematosus and lupus nephritis. Autoimmun. Rev. *19*, 102668.

27. Saxena, R., Mahajan, T., and Mohan, C. (2011). Lupus nephritis: current update. Arthritis Res. Ther. *13*, 240.

28. Schönbeck, U., and Libby, P. (2001). The CD40/CD154 receptor/ligand dyad. Cell. Mol. Life Sci. *58*, 4–43.

29. Enghard, P., Humrich, J.Y., Rudolph, B., Rosenberger, S., Biesen, R., Kuhn, A., Manz, R., Hiepe, F., Radbruch, A., Burmester, G.R., and Riemekasten, G. (2009). CXCR3+CD4+T Cells Are Enriched in Inflamed Kidneys and Urine and Provide a New Biomarker for Acute Nephritis Flares in Systemic Lupus Erythematosus Patients. Arthritis Rheum. *60*, 199–206.

30. He, H., Suryawanshi, H., Morozov, P., Gay-Mimbrera, J., Del Duca, E., Kim, H.J., Kameyama, N., Estrada, Y., Der, E., Krueger, J.G., et al. (2020). Single-cell transcriptome analysis of human skin identifies novel fibroblast subpopulation and enrichment of immune subsets in atopic dermatitis. J. Allergy Clin. Immunol. *145*, 1615–1628.

31. Esaki, H., Ewald, D.A., Ungar, B., Rozenblit, M., Zheng, X., Xu, H., Estrada, Y.D., Peng, X., Mitsui, H., Litman, T., et al. (2015). Identification of novel immune and barrier genes in atopic dermatitis by means of laser capture microdissection. J. Allergy Clin. Immunol. *135*, 153–163.

32. Vickery, B.P. (2007). Skin barrier function in atopic dermatitis. Curr. Opin. Pediatr. *19*, 89–93.

33. Sonnenberg-Riethmacher, E., Miehe, M., and Riethmacher, D. (2021). Periostin in Allergy and Inflammation. Front. Immunol. *12*, 722170.

34. Sun, Z., Kim, J.H., Kim, S.H., Kim, H.R., Zhang, K., Pan, Y., Ko, M.K., Kim, B.M., Chu, H., Lee, H.R., et al. (2021). Skin-resident natural killer T cells participate in cutaneous allergic inflammation in atopic dermatitis. J. Allergy Clin. Immunol. *147*, 1764–1777.

35. Tyler, S.R., Rotti, P.G., Sun, X., Yi, Y., Xie, W., Winter, M.C., Flamme-Wiese, M.J., Tucker, B.A., Mullins, R.F., Norris, A.W., and Engelhardt, J.F. (2019). PyMINEr Finds Gene and Autocrine-Paracrine Networks from Human Islet scRNA-Seq. Cell Rep. *26*, 1951–1964.e8.

36. Caicedo, A. (2013). Paracrine and autocrine interactions in the human islet: More than meets the eye. Semin. Cell Dev. Biol. *24*, 11–21.

37. Bosco, D., Armanet, M., Morel, P., Niclauss, N., Sgroi, A., Muller, Y.D., Giovannoni, L., Parnaud, G., and Berney, T. (2010). Unique Arrangement of alpha- and beta-Cells in Human Islets of Langerhans. Diabetes *59*, 1202–1210.

38. Jain, R., and Lammert, E. (2009). Cell-cell interactions in the endocrine pancreas. Diabetes Obes. Metabol. *11*, 159–167.

39. McDaniel, M.L., Marshall, C.A., Pappan, K.L., and Kwon, G. (2002). Metabolic and autocrine regulation of the mammalian target of rapamycin by pancreatic beta-cells. Diabetes *51*, 2877–2885.

40. Modi, H., Jacovetti, C., Tarussio, D., Metref, S., Madsen, O.D., Zhang, F.P., Rantakari, P., Poutanen, M., Nef, S., Gorman, T., et al. (2015). Autocrine Action of IGF2 Regulates Adult beta-Cell Mass and Function. Diabetes *64*, 4148–4157.

41. Kulkarni, R.N., Holzenberger, M., Shih, D.Q., Ozcan, U., Stoffel, M., Magnuson, M.A., and Kahn, C.R. (2002). beta-cell-specific deletion of the Igf1 receptor leads to hyperinsulinemia and glucose intolerance but does not alter beta-cell mass. Nat. Genet. *31*, 111–115.

42. Kulkarni, R.N., Brüning, J.C., Winnay, J.N., Postic, C., Magnuson, M.A., and Kahn, C.R. (1999). Tissue-specific knockout of the insulin receptor in pancreatic beta cells creates an insulin secretory defect similar to that in type 2 diabetes. Cell *96*, 329–339.

43. Hartig, S.M., and Cox, A.R. (2020). Paracrine signaling in islet function and survival. J. Mol. Med. *98*, 451–467.

44. Taborsky, G.J., Smith, P.H., and Porte, D. (1979). DIFFERENTIAL EFFECTS OF SOMATOSTATIN ANALOGS ON ALPHA-CELLS AND BETA-CELLS OF THE PANCREAS. Am. J. Physiol. *236*, E123–E128.

45. Kumar, U., Sasi, R., Suresh, S., Patel, A., Thangaraju, M., Metrakos, P., Patel, S.C., and Patel, Y.C. (1999). Subtype-selective expression of the five somatostatin receptors (hSSTR1-5) in human pancreatic islet cells - A quantitative double-label immunohistochemical analysis. Diabetes *48*, 77–85.

46. Klaff, L.J., and Taborsky, G.J. (1987). PANCREATIC SOMATOSTATIN IS A MEDIATOR OF GLUCAGON-INHIBITION BY HYPERGLYCEMIA. Diabetes *36*, 592–596.

47. Strowski, M.Z., Parmar, R.M., Blake, A.D., and Schaeffer, J.M. (2000). Somatostatin inhibits insulin and glucagon secretion via two receptor subtypes: An in vitro study of pancreatic islets from somatostatin receptor 2 knockout mice. Endocrinology *141*, 111–117.

48. Cejvan, K., Coy, D.H., and Efendic, S. (2003). Intra-islet somatostatin regulates glucagon release via type 2 somatostatin receptors in rats. Diabetes *52*, 1176–1181.

49. Rodriguez-Diaz, R., Tamayo, A., Hara, M., and Caicedo, A. (2020). The Local Paracrine Actions of the Pancreatic alpha-Cell. Diabetes *69*, 550–558.

50. Meda, P. (2018). Gap junction proteins are key drivers of endocrine function. Biochim. Biophys. Acta Biomembr. *1860*, 124–140.

51. Moede, T., Leibiger, I.B., and Berggren, P.-O. (2020). Alpha cell regulation of beta cell function. Diabetologia *63*, 2064–2075.

52. Rodriguez-Diaz, R., Molano, R.D., Weitz, J.R., Abdulreda, M.H., Berman, D.M., Leibiger, B., Leibiger, I.B., Kenyon, N.S., Ricordi, C., Pileggi, A., et al. (2018). Paracrine Interactions within the Pancreatic Islet Determine the Glycemic Set Point. Cell Metabol. *27*, 549–558.e4.

53. Gromada, J., Franklin, I., and Wollheim, C.B. (2007). alpha-Cells of the endocrine pancreas: 35 years of research but the enigma remains. Endocr. Rev. *28*, 84–116.

54. Franklin, I., Gromada, J., Gjinovci, A., Theander, S., and Wollheim, C.B. (2005). beta-Cell secretory products activate alpha-cell ATP-dependent potassium channels to inhibit glucagon release. Diabetes *54*, 1808–1815.

55. Kawamori, D., Kurpad, A.J., Hu, J., Liew, C.W., Shih, J.L., Ford, E.L., Herrera, P.L., Polonsky, K.S., McGuinness, O.P., and Kulkarni, R.N. (2009). Insulin Signaling in alpha Cells Modulates Glucagon Secretion In Vivo. Cell Metabol. *9*, 350–361.

56. Gupta, K., Levinsohn, J., Linderman, G., Chen, D., Sun, T.Y., Dong, D., Taketo, M.M., Bosenberg, M., Kluger, Y., Choate, K., and Myung, P. (2019). Single-Cell Analysis Reveals a Hair Follicle Dermal Niche Molecular Differentiation Trajectory that Begins Prior to Morphogenesis. Dev. Cell *48*, 17–31.e6.

57. Fu, J., and Hsu, W. (2013). Epidermal Wnt Controls Hair Follicle Induction by Orchestrating Dynamic Signaling Crosstalk between the Epidermis and Dermis. J. Invest. Dermatol. *133*, 890–898.

58. Chen, D., Jarrell, A., Guo, C., Lang, R., and Atit, R. (2012). Dermal beta-catenin activity in response to epidermal Wnt ligands is required for fibroblast proliferation and hair follicle initiation. Development *139*, 1522–1533.

59. Peus, D., and Pittelkow, M.R. (1996). Growth factors in hair organ development and the hair growth cycle. Dermatol. Clin. *14*, 559–572.

60. Ducros, D.L. (1993). FIBROBLAST GROWTH-FACTOR AND EPIDERMAL GROWTH-FACTOR IN HAIR DEVELOPMENT. J. Invest. Dermatol. *101*, S106–S113.

61. Glover, J.D., Wells, K.L., Matthaus, F., Painter, K.J., Ho, W., Riddell, J., Johansson, J.A., Ford, M.J., Jahoda, C.A.B., Klika, V., et al. (2017). Hierarchical patterning modes orchestrate hair follicle morphogenesis. PLoS Biol. *15*, 31.

62. Huh, S.H., Närhi, K., Lindfors, P.H., Häärä, O., Yang, L., Ornitz, D.M., and Mikkola, M.L. (2013). Fgf20 governs formation of primary and secondary dermal condensations in developing hair follicles. Genes Dev. *27*, 450–458.

63. Mukhopadhyay, A., Krishnaswami, S.R., Cowing-Zitron, C., Hung, N.J., Reilly-Rhoten, H., Burns, J., and Yu, B.D. (2013). Negative regulation of Shh levels by Kras and Fgfr2 during hair follicle development. Dev. Biol. *373*, 373–382.

64. Petiot, A., Conti, F.J.A., Grose, R., Revest, J.M., Hodivala-Dilke, K.M., and Dickson, C. (2003). A crucial role for Fgfr2-IIIb signalling in epidermal development and hair follicle patterning. Development *130*, 5493–5501.

65. Richardson, G.D., Bazzi, H., Fantauzzo, K.A., Waters, J.M., Crawford, H., Hynd, P., Christiano, A.M., and Jahoda, C.A.B. (2009). KGF and EGF signalling block hair follicle induction and promote interfollicular epidermal fate in developing mouse skin. Development *136*, 2153–2164.

66. Zeisel, A., Muñoz-Manchado, A.B., Codeluppi, S., Lönnerberg, P., La Manno, G., Juréus, A., Marques, S., Munguba, H., He, L., Betsholtz, C., et al. (2015). Cell types in the mouse cortex and hippocampus revealed by single-cell RNA-seq. Science *347*, 1138–1142.

67. Sheikh, B.N., Bondareva, O., Guhathakurta, S., Tsang, T.H., Sikora, K., Aizarani, N., Sagar Holz, H., Holz, H., Grün, D., Hein, L., and Akhtar, A. (2019). Systematic Identification of Cell-Cell Communication Networks in the Developing Brain. iScience *21*, 273–287.

68. Daneman, R., Zhou, L., Agalliu, D., Cahoy, J.D., Kaushal, A., and Barres, B.A. (2010). The Mouse Blood-Brain Barrier Transcriptome: A New Resource for Understanding the Development and Function of Brain Endothelial Cells. PLoS One *5*, e13741.

69. Matcovitch-Natan, O., Winter, D.R., Giladi, A., Vargas Aguilar, S., Spinrad, A., Sarrazin, S., Ben-Yehuda, H., David, E., Zelada González, F., Perrin, P., et al. (2016). Microglia development follows a stepwise program to regulate brain homeostasis. Science *353*, aad8670.

70. Mathys, H., Adaikkan, C., Gao, F., Young, J.Z., Manet, E., Hemberg, M., De Jager, P.L., Ransohoff, R.M., Regev, A., and Tsai, L.H. (2017). Temporal Tracking of Microglia Activation in Neurodegeneration at Single-Cell Resolution. Cell Rep. *21*, 366–380.

71. Sengillo, J.D., Winkler, E.A., Walker, C.T., Sullivan, J.S., Johnson, M., and Zlokovic, B.V. (2013). Deficiency in Mural Vascular Cells Coincides with Blood–Brain Barrier Disruption in Alzheimer's Disease. Brain Pathol. *23*, 303–310.

72. Vasudevan, A., Long, J.E., Crandall, J.E., Rubenstein, J.L.R., and Bhide, P.G. (2008). Compartment-specific transcription factors orchestrate angiogenesis gradients in the embryonic brain. Nat. Neurosci. *11*, 429–439.

73. Hatori, K., Nagai, A., Heisel, R., Ryu, J.K., and Kim, S.U. (2002). Fractalkine and fractalkine receptors in human neurons and glial cells. J. Neurosci. Res. *69*, 418–426.

74. Kim, K.W., Vallon-Eberhard, A., Zigmond, E., Farache, J., Shezen, E., Shakhar, G., Ludwig, A., Lira, S.A., and Jung, S. (2011). In vivo

CellPress
OPEN ACCESS

structure/function and expression analysis of the CX3C chemokine fractalkine. Blood *118*, E156–E167.

75. Szepesi, Z., Manouchehrian, O., Bachiller, S., and Deierborg, T. (2018). Bidirectional Microglia-Neuron Communication in Health and Disease. Front. Cell. Neurosci. *12*, 323.

76. Al-Aoukaty, A., Rolstad, B., Giaid, A., and Maghazachi, A.A. (1998). MIP-3α, MIP-3β and fractalkine induce the locomotion and the mobilization of intracellular calcium, and activate the heterotrimeric G proteins in human natural killer cells. Immunology *95*, 618–624.

77. Chandrasekar, B., Mummidi, S., Perla, R.P., Bysani, S., Dulin, N.O., Liu, F., and Melby, P.C. (2003). Fractalkine (CX3CL1) stimulated by nuclear factor kappa B (NF-kappa B)-dependent inflammatory signals induces aortic smooth muscle cell proliferation through an autocrine pathway. Biochem. J. *373*, 547–558.

78. Nikolakopoulou, A.M., Dutta, R., Chen, Z., Miller, R.H., and Trapp, B.D. (2013). Activated microglia enhance neurogenesis via trypsinogen secretion. Proc. Natl. Acad. Sci. USA *110*, 8714–8719.

79. Alliot, F., Godin, I., and Pessac, B. (1999). Microglia derive from progenitors, originating from the yolk sac, and which proliferate in the brain. Dev. Brain Res. *117*, 145–152.

80. Stremmel, C., Schuchert, R., Wagner, F., Thaler, R., Weinberger, T., Pick, R., Mass, E., Ishikawa-Ankerhold, H.C., Margraf, A., Hutter, S., et al. (2018). Yolk sac macrophage progenitors traffic to the embryo during defined stages of development. Nat. Commun. *9*, 3699.

81. Paolicelli, R.C., Bolasco, G., Pagani, F., Maggi, L., Scianni, M., Panzanelli, P., Giustetto, M., Ferreira, T.A., Guiducci, E., Dumas, L., et al. (2011). Synaptic Pruning by Microglia Is Necessary for Normal Brain Development. Science *333*, 1456–1458.

82. Zhan, Y., Paolicelli, R.C., Sforazzini, F., Weinhard, L., Bolasco, G., Pagani, F., Vyssotski, A.L., Bifone, A., Gozzi, A., Ragozzino, D., and Gross, C.T. (2014). Deficient neuron-microglia signaling results in impaired functional brain connectivity and social behavior. Nat. Neurosci. *17*, 400–406.

83. Chamera, K., Trojan, E., Szuster-Głuszczak, M., and Basta-Kaim, A. (2020). The Potential Role of Dysfunctions in Neuron-Microglia Communication in the Pathogenesis of Brain Disorders. Curr. Neuropharmacol. *18*, 408–430.

84. González-Castillo, C., Ortuño-Sahagún, D., Guzmán-Brambila, C., Pallàs, M., and Rojas-Mayorquín, A.E. (2015). Pleiotrophin as a central nervous system neuromodulator evidences from the hippocampus. Front. Cell. Neurosci. *8*, 443.

85. Nakanishi, K., Tokita, Y., Aono, S., Ida, M., Matsui, F., Higashi, Y., and Oohira, A. (2010). Neuroglycan C, A Brain-Specific Chondroitin Sulfate Proteoglycan, Interacts with Pleiotrophin, A Heparin-Binding Growth Factor. Neurochem. Res. *35*, 1131–1137.

86. Haigh, J.J., Morelli, P.I., Gerhardt, H., Haigh, K., Tsien, J., Damert, A., Miquerol, L., Muhlner, U., Klein, R., Ferrara, N., et al. (2003). Cortical and retinal defects caused by dosage-dependent reductions in VEGF-A paracrine signaling. Dev. Biol. *262*, 225–241.

87. Ma, S., Santhosh, D., Kumar T, P., and Huang, Z. (2017). A Brain-Region-Specific Neural Pathway Regulating Germinal Matrix Angiogenesis. Dev. Cell *41*, 366–381.e4.

88. Bazzi, H., Fantauzzo, K.A., Richardson, G.D., Jahoda, C.A.B., and Christiano, A.M. (2007). Transcriptional profiling of developing mouse epidermis reveals novel patterns of coordinated gene expression. Dev. Dynam. *236*, 961–970.

89. Byrne, C., Tainsky, M., and Fuchs, E. (1994). Programming gene expression in developing epidermis. Development *120*, 2369–2383.

90. Fu, J., and Hsu, W. (2013). Epidermal Wnt controls hair follicle induction by orchestrating dynamic signaling crosstalk between the epidermis and dermis. J. Invest. Dermatol. *133*, 890–898.

91. Ge, W., Tan, S.J., Wang, S.H., Li, L., Sun, X.F., Shen, W., and Wang, X. (2020). Single-cell Transcriptome Profiling reveals Dermal and Epithelial cell fate decisions during Embryonic Hair Follicle Development. Theranostics *10*, 7581–7598.

92. Nichol, P.F., Corliss, R.F., Yamada, S., Shiota, K., and Saijoh, Y. (2012). Muscle patterning in mouse and human abdominal wall development and omphalocele specimens of humans. Anat. Rec. *295*, 2129–2140.

93. Weidinger, S., and Novak, N. (2016). Atopic dermatitis. Lancet *387*, 1109–1122.

94. Suárez-Fariñas, M., Tintle, S.J., Shemer, A., Chiricozzi, A., Nograles, K., Cardinale, I., Duan, S., Bowcock, A.M., Krueger, J.G., and Guttman-Yassky, E. (2011). Nonlesional atopic dermatitis skin is characterized by broad terminal differentiation defects and variable immune abnormalities. J. Allergy Clin. Immunol. *127*, 954–964.e1-4.

95. Kezic, S., O'Regan, G.M., Lutter, R., Jakasa, I., Koster, E.S., Saunders, S., Caspers, P., Kemperman, P.M., Puppels, G.J., Sandilands, A., et al. (2012). Filaggrin loss-of-function mutations are associated with enhanced expression of IL-1 cytokines in the stratum corneum of patients with atopic dermatitis and in a murine model of filaggrin deficiency. J. Allergy Clin. Immunol. *129*, 1031–1039.e1031.

96. Oyoshi, M.K., Larson, R.P., Ziegler, S.F., and Geha, R.S. (2010). Mechanical injury polarizes skin dendritic cells to elicit a T(H)2 response by inducing cutaneous thymic stromal lymphopoietin expression. J. Allergy Clin. Immunol. *126*, 976–984.e5.

97. Homey, B., Steinhoff, M., Ruzicka, T., and Leung, D.Y.M. (2006). Cytokines and chemokines orchestrate atopic skin inflammation. J. Allergy Clin. Immunol. *118*, 178–189.

98. Sagerström, C.G., Gammill, L.S., Veale, R., and Sive, H. (2005). Specification of the enveloping layer and lack of autoneuralization in zebrafish embryonic explants. Dev. Dynam. *232*, 85–97.

99. Wagner, D.E., Weinreb, C., Collins, Z.M., Briggs, J.A., Megason, S.G., and Klein, A.M. (2018). Single-cell mapping of gene expression landscapes and lineage in the zebrafish embryo. Science *360*, 981–987.

100. Li, L., Song, L., Liu, C., Chen, J., Peng, G., Wang, R., Liu, P., Tang, K., Rossant, J., and Jing, N. (2015). Ectodermal progenitors derived from epiblast stem cells by inhibition of Nodal signaling. J. Mol. Cell Biol. *7*, 455–465.

101. Caneparo, L., Pantazis, P., Dempsey, W., and Fraser, S.E. (2011). Intercellular Bridges in Vertebrate Gastrulation. PLoS One *6*.

102. Abboud, D., and Hanson, J. (2017). Chemokine neutralization as an innovative therapeutic strategy for atopic dermatitis. Drug Discov. Today *22*, 702–711.

103. Harms, M., Habib, M.M.W., Nemska, S., Nicolò, A., Gilg, A., Preising, N., Sokkar, P., Carmignani, S., Raasholm, M., Weidinger, G., et al. (2021). An optimized derivative of an endogenous CXCR4 antagonist prevents atopic dermatitis and airway inflammation. Acta Pharm. Sin. B *11*, 2694–2708.

104. Lim, X., Tan, S.H., Koh, W.L.C., Chau, R.M.W., Yan, K.S., Kuo, C.J., van Amerongen, R., Klein, A.M., and Nusse, R. (2013). Interfollicular epidermal stem cells self-renew via autocrine Wnt signaling. Science *342*, 1226–1230.

105. Noel, F., Massenet-Regad, L., Carmi-Levy, I., Cappuccio, A., Grandclaudon, M., Trichot, C., Kieffer, Y., Mechta-Grigoriou, F., and Soumelis, V. (2021). Dissection of intercellular communication using the transcriptome-based framework ICELLNET. Nat. Commun. *12*.

106. Bossen, C., Ingold, K., Tardivel, A., Bodmer, J.L., Gaide, O., Hertig, S., Ambrose, C., Tschopp, J., and Schneider, P. (2006). Interactions of tumor necrosis factor (TNF) and TNF receptor family members in the mouse and human. J. Biol. Chem. *281*, 13964–13971.

107. Gu, C., Wu, L., and Li, X. (2013). IL-17 family: Cytokines, receptors and signaling. Cytokine *64*, 477–485.

108. Richelle, A., Joshi, C., and Lewis, N.E. (2019). Assessing key decisions for transcriptomic data integration in biochemical networks. PLoS Comput. Biol. *15*, e1007185.

109. Richelle, A., Kellman, B.P., Wenzel, A.T., Chiang, A.W.T., Reagan, T., Gutierrez, J.M., Joshi, C., Li, S., Liu, J.K., Masson, H., et al. (2021).

Model-based assessment of mammalian cell metabolic functionalities using omics data. Cell Rep. Methods *1*.

110. Jiang, Q., Zhang, S., and Wan, L. (2022). Dynamic inference of cell developmental complex energy landscape from time series single-cell transcriptomic data. PLoS Comput. Biol. *18*.

111. Zhang, Y., Chang, X., and Liu, X. (2021). Inference of gene regulatory networks using pseudo-time series data. Bioinformatics *37*, 2423–2431.

112. Liu, Y. (2023). MDIC3 (Figshare). https://doi.org/10.6084/m9.figshare.24486046.v1.

113. Holter, N.S., Maritan, A., Cieplak, M., Fedoroff, N.V., and Banavar, J.R. (2001). Dynamic modeling of gene expression data. Proc. Natl. Acad. Sci. USA *98*, 1693–1698.

114. Bansal, M., Della Gatta, G., and di Bernardo, D. (2006). Inference of gene regulatory networks and compound mode of action from time course gene expression profiles. Bioinformatics *22*, 815–822.

115. Wu, S., Liu, Z.P., Qiu, X., and Wu, H. (2014). Modeling genome-wide dynamic regulatory network in mouse lungs with influenza infection using high-dimensional ordinary differential equations. PLoS One *9*, e95276.

116. Yeung, M.K.S., Tegnér, J., and Collins, J.J. (2002). Reverse engineering gene networks using singular value decomposition and robust regression. Proc. Natl. Acad. Sci. USA *99*, 6163–6168.

117. Davidson, E.H., and Levine, M.S. (2008). Properties of developmental gene regulatory networks. Proc. Natl. Acad. Sci. USA *105*, 20063–20066.

118. Huynh-Thu, V.A., Irrthum, A., Wehenkel, L., and Geurts, P. (2010). Inferring regulatory networks from expression data using tree-based methods. PLoS One *5*, e12776.

119. Moerman, T., Aibar Santos, S., Bravo González-Blas, C., Simm, J., Moreau, Y., Aerts, J., and Aerts, S. (2019). GRNBoost2 and Arboreto: efficient and scalable inference of gene regulatory networks. Bioinformatics *35*, 2159–2161.

Patterns, Volume *5*

Supplemental information

MDIC3: Matrix decomposition

to infer cell-cell communication

Yi Liu, Yuelei Zhang, Xiao Chang, and Xiaoping Liu

Supplemental Figures

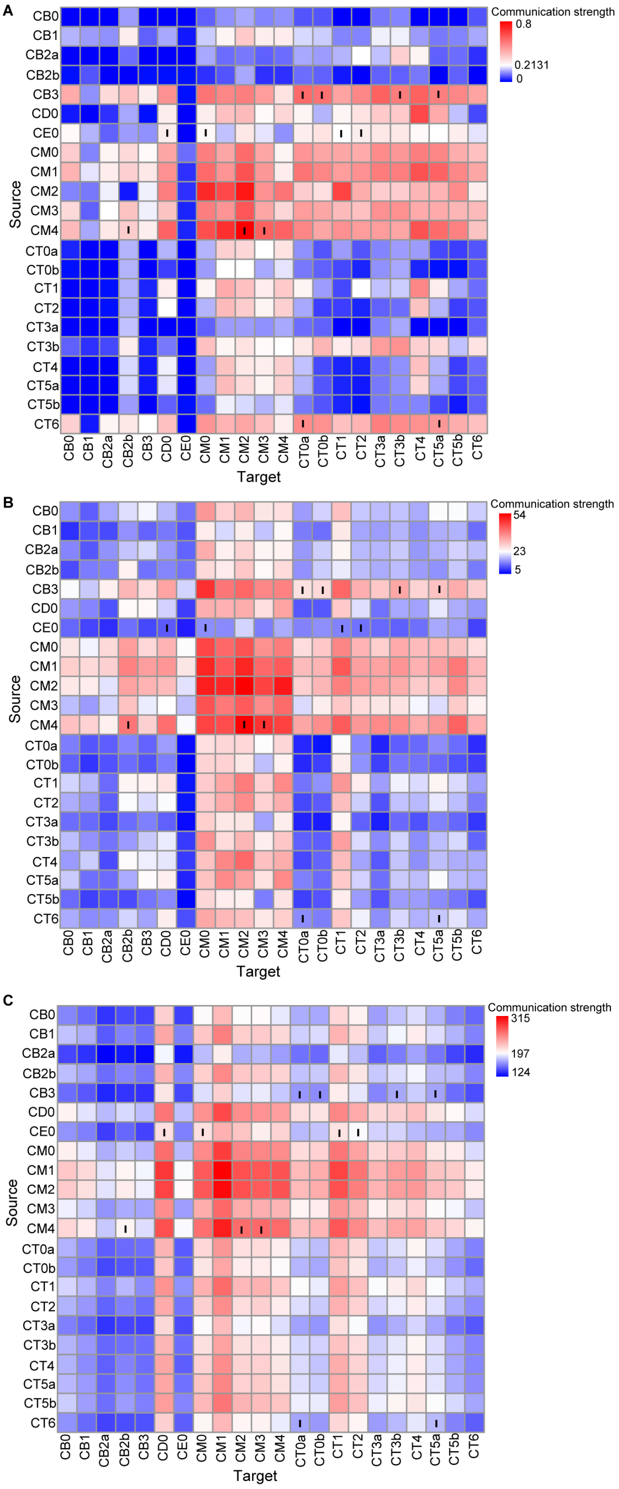

Figure S1. Heatmaps of the inferred intercellular communications on lupus nephritis dataset by existing methods. The heat maps showing the communication among cell types in human lupus nephritis were inferred by **(A)** CellChat, **(B)** Cellphonedb, and **(C)** iTALK. The black "I" indicates interactions mentioned in this main text. The row represents the "source" of a communication, and the column represents the "target" of the communication. For example, the black "I" in row "CB3" and column "CT0a" indicates an intercellular communication from CB3 to CT0a.

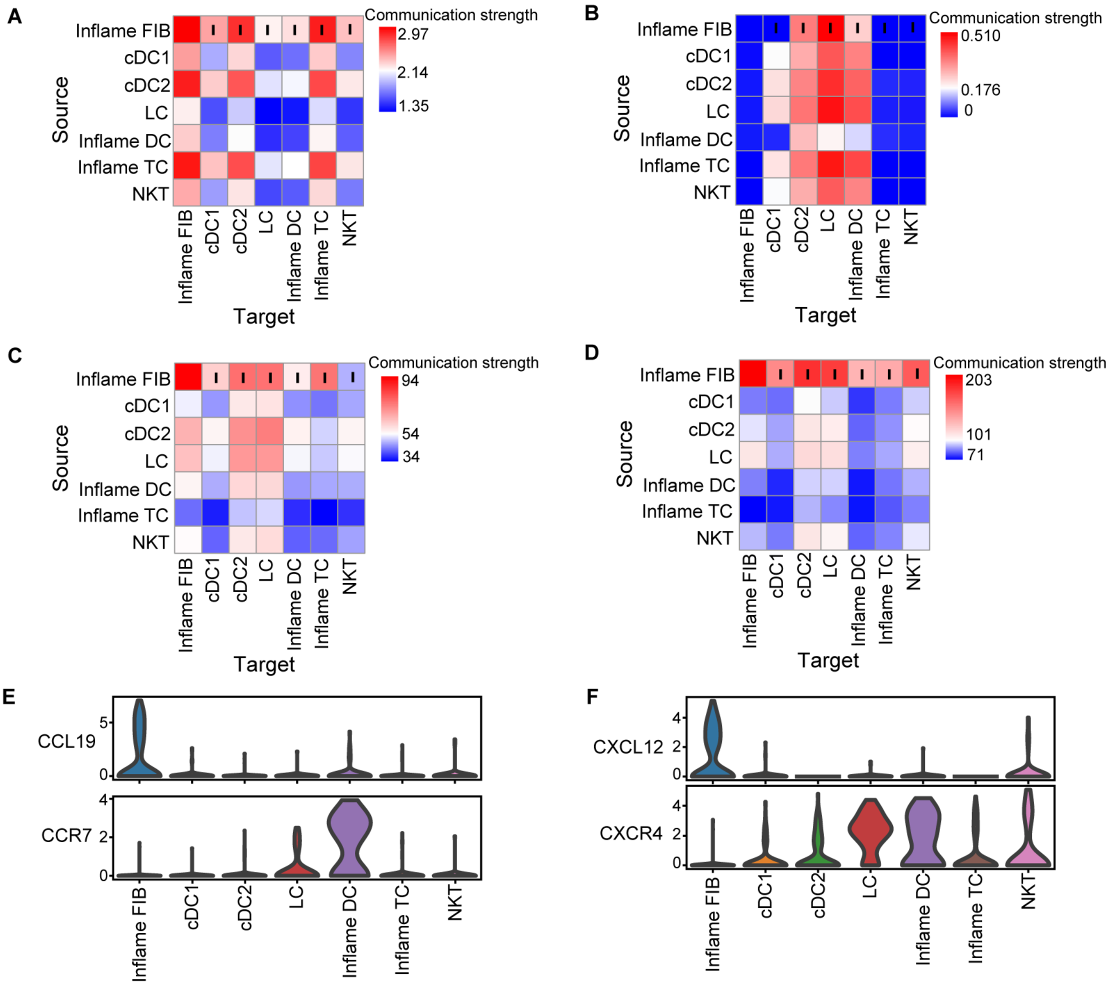

Figure S2. Case study on human lesional skin dataset. The heat maps showing the communication results among cell types in human lesional skin were inferred by **(A)** MDIC3, **(B)** CellChat, **(C)** Cellphonedb, and **(D)** iTALK, respectively. The black "I" indicates interactions mentioned in this main text. The row represents the "source" of a communication, and the column represents the "target" of the communication. For example, the black "I" in row "Inflame FIB" and column "cDC1" means an intercellular communication from Inflame FIB to cDC1. Inflame FIB: inflammatory fibroblasts; cDC: conventional dendritic cell; Inflame DC: inflammatory dendritic cell; LC: Langerhans cell; Inflame TC: inflammatory T cell; NKT: natural killer T cell. **(E)** Violin plot shows the expression of *CCL19-CCR7* signaling in different human lesional skin cell types. **(F)** Violin plot shows the expression of *CXCL12-CXCR4* signaling in different human lesional skin cell types.

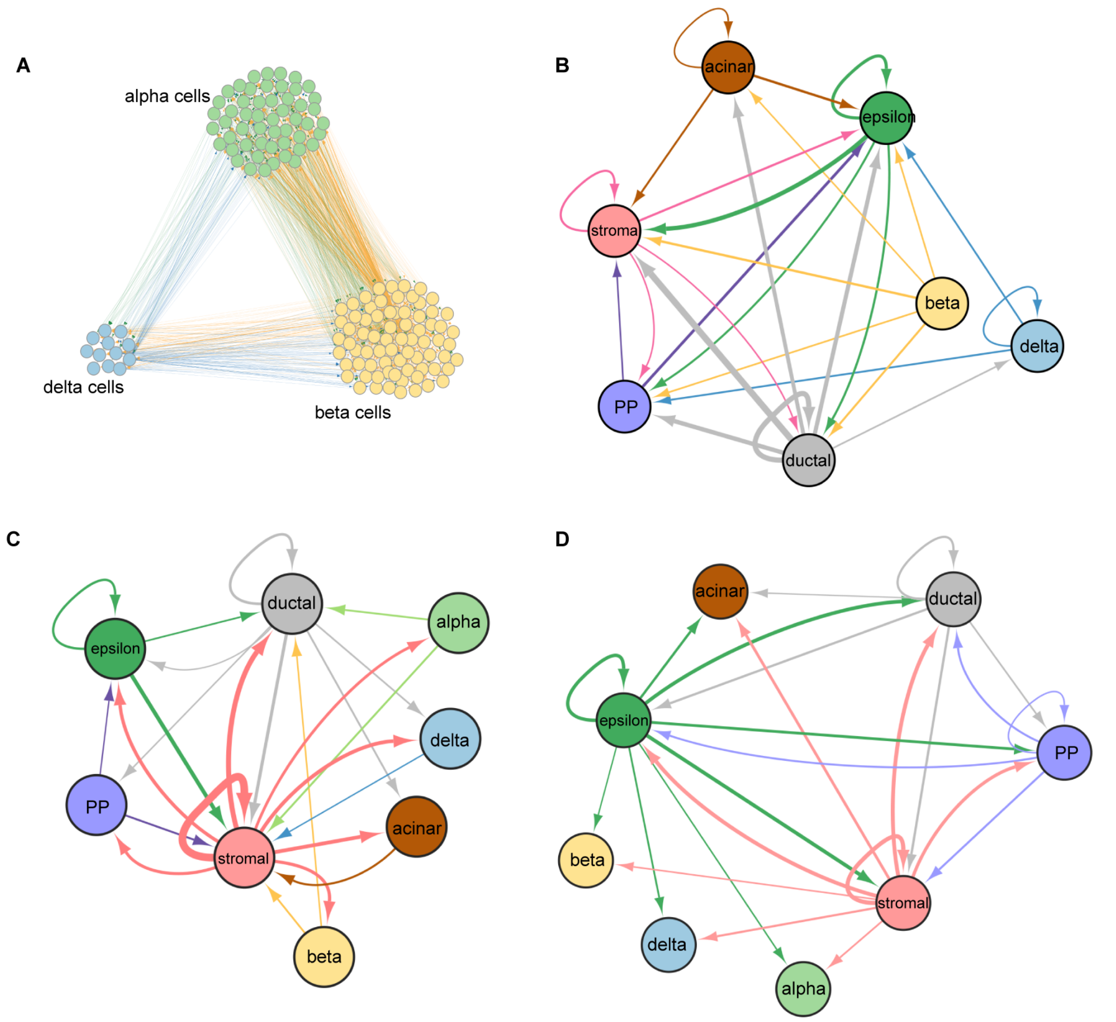

Figure S3. Intercellular communications among human islet cells by MDIC3, CellChat, Cellphonedb, and iTALK. (A) Cell-cell communications among alpha cells, beta cells, and delta cells were inferred by MDIC3. The line with different color indicates that the communication signal is sent from the cell with the same color as the line. The figure shows paracrine interactions among the three cell types. The communication networks among cell types in human islet were inferred by **(B)** CellChat, **(C)** Cellphonedb, and **(D)** iTALK, respectively. The arrow indicates the direction of signal transference, and the edge width represents the communication strength.

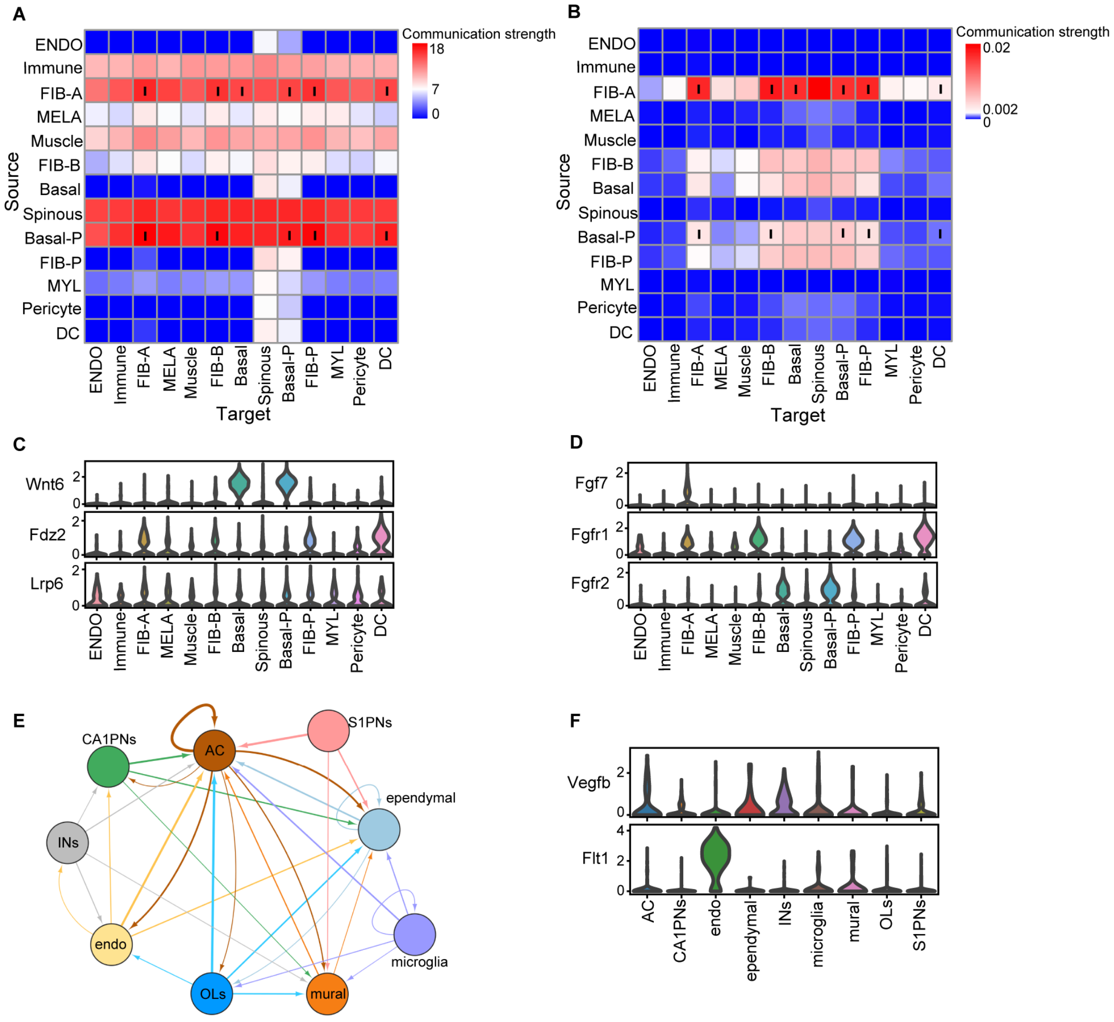

Figure S4. Case study on mouse species datasets. (A) and **(B)** are heatmaps showing the communication results among cell types in E14.5 mouse skin inferred by MDIC3 and CellChat, respectively. Black "I" indicates interactions mentioned in this main text. The row represents the "source" of a communication, and the column represents the "target" of the communication. For example, the black "I" in row "FIB-A" and column "FIB-B" means an intercellular communication from FIB-A to FIB-B. **(C)** Violin plot shows the expression of *Wnt6 - (Fzd2+Lrp6)* gene pairs under the WNT signaling in E14.5 mouse skin cell types. **(D)** Violin plot shows the expression *Fgf7-Fgfr1* and *Fgf7-Fgfr2* gene pairs under the FGF signaling in E14.5 mouse skin cell types. ENDO: endothelial cells; Immune: immune cells; FIB-A: fibroblast type A; MELA: melanocyte; Muscle: muscle cells; FIB-B: fibroblast type B; Basal: basal cells; Spinous: spinous epithelial cells; Basal-P: proliferative basal cells; FIB-P: proliferative fibroblasts; MYL: myeloid cells; DC: Dendritic cells. **(E)** The communication network among cell types in mouse brain was inferred by CellChat. The arrow indicates the direction of signal transference. The edge width represents the communication strength. S1PNs: S1 pyramidal neurons; CA1PNs: CA1 pyramidal neurons; INs: interneurons; OLs: oligodendrocytes; AC: astrocytes; endo: vascular endothelial cells. **(F)** Violin plot shows the expression of *Vegfb-Flt1* L-R signaling for different mouse brain cell types.

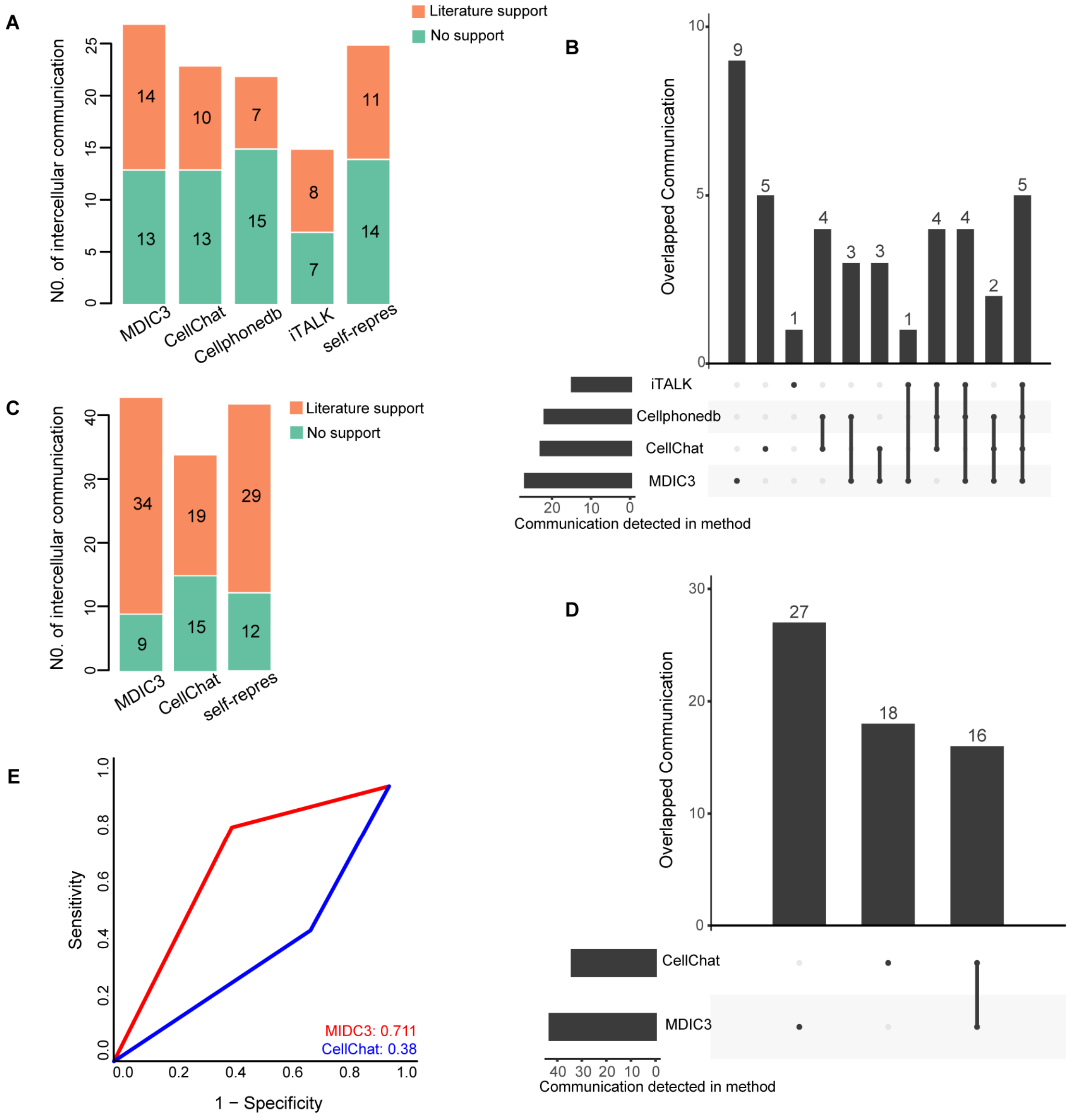

Figure S5. Comparison of the predictive performance of MDIC3 with other methods using scRNA-seq. (A) Comparison of literature support (Table S3) for the intercellular communications by MDIC3 and four other tools (CellChat, Cellphonedb, iTALK, and the two-side self-representation model) on human lesional skin dataset. The horizontal ordinate represents the five methods, and the vertical ordinate is the number of intercellular communications from the five methods. The orange bar represents the number of intercellular communications with literature support, and the green bar represents the number of intercellular communications without literature support from PubMed database (https://pubmed.ncbi.nlm.nih.gov/). **(B)** UpSetR plot of intercellular communications on human lesional skin dataset from four tools (MDIC3, CellChat, Cellphonedb, and iTALK). The horizontal bar graph at the bottom left represents the total number of cell-cell communications detected by different methods. Use a dot to refer to the corresponding method on the left. If cell-cell communications were only detected by one method, only one gray dot is darkened, and the number of the detected cell-cell communications are shown in bar graph form above. The intersection of the cell-cell communication results inferred by

different methods are shown by multiple black dots and a line connecting them, and the number of the intersection of intercellular communication results is represented by the bar graph form above. **(C)** Comparison of literature support (Table S4) for intercellular communications by MDIC3, CellChat and the two-side self-representation model on mouse brain dataset. **(D)** UpSetR plot of intercellular communications on mouse brain dataset from MDIC3 and CellChat. **(E)** ROC curves of MDIC3 and CellChat for literature support (Table S4) on mouse brain dataset.

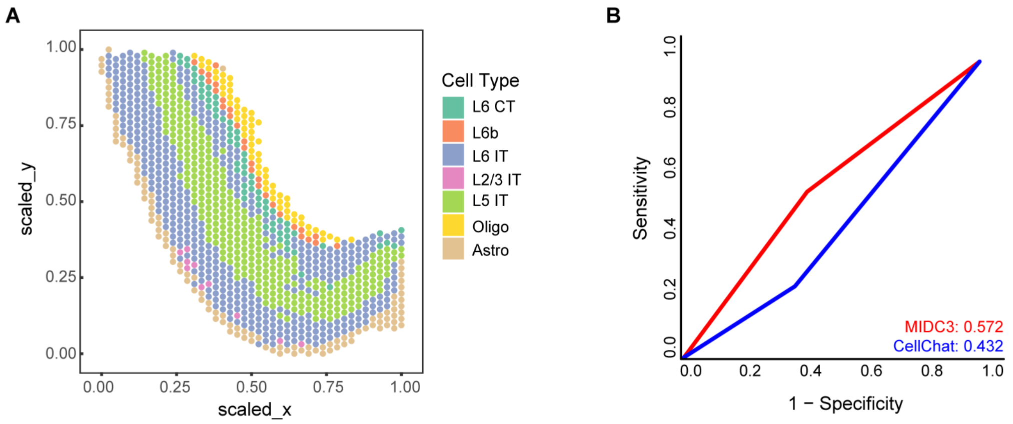

Figure S6. Comparison of the predictive performance of MDIC3 with CellChat using spatial dataset. (A) Spatial plot with pie chart shows the spatial location of each spot. The spot labels were selected by the cell type with the largest proportion of each spot. **(B)** ROC curves of MDIC3 and CellChat for literature support (Table S5) on mouse brain 10X visium spatial dataset.

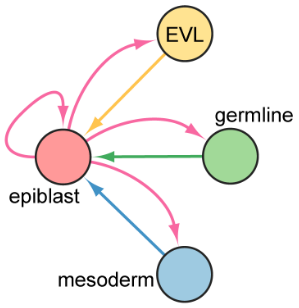

Figure S7. The communication networks among cell types in 4hpf zebrafish embryo inferred by MDIC3

Supplemental Tables

Table S2. Literature curated of the inferred cellular communications between human islet endocrine cells

Cellular Communication	MDIC3	CellChat	Cellphonedb	iTALK	Self-representation	PMID
alpha-alpha	1	0	0	0	1	19817798
alpha-beta	1	0	0	0	1	23022232;19817798
alpha-delta	1	0	0	0	1	32067063;19602585;682984;27390011
alpha-epsilon	0	0	0	0	0	no
alpha-pp	0	0	0	0	1	no
beta-alpha	1	0	0	0	1	23022232;19817798
beta-beta	1	0	0	0	1	26384384;19817798
beta-delta	1	0	0	0	1	no
beta-epsilon	0	1	0	0	0	no
beta-pp	1	1	0	0	1	no
delta-alpha	1	0	0	0	1	32067063
delta-beta	1	0	0	0	1	32067063
delta-delta	0	1	0	0	1	32067063;27390011;27408771
delta-epsilon	0	1	0	0	0	no
delta-pp	0	1	0	0	0	no
epsilon-alpha	0	0	0	1	0	19817798;27408771
epsilon-beta	1	0	0	1	0	19817798;27408771
epsilon-delta	0	0	0	1	0	32067063; 27390011
epsilon-epsilon	0	1	1	1	0	no
epsilon-pp	0	1	0	1	0	no
pp-alpha	1	0	0	0	1	32067063;25445712
pp-beta	1	0	0	0	1	32067063;28069397

pp-delta	0	0	0	0	0	no
pp-epsilon	0	1	1	1	0	no
pp-pp	0	0	0	1	0	no

Table S3. Literature curated of the inferred cellular communications between human lesional skin cells

Cellular Communication	MDIC3	CellChat	Cellphonedb	iTALK	Self-representation	PMID
Inflame FIB-Inflame FIB	1	0	1	1	1	no
Inflame FIB-cDC1	1	0	1	1	1	20538798
Inflame FIB-cDC2	1	1	1	1	1	20538798
Inflame FIB-LC	1	1	1	1	1	20538798
Inflame FIB-Inflame DC	1	1	1	1	1	20538798；32035984
Inflame FIB-Inflame TC	1	0	1	1	1	32035984；25182982
Inflame FIB-NKT	1	0	0	1	1	20538798；33516870
cDC1-Inflame FIB	1	0	0	0	1	no
cDC1-cDC2	1	1	1	0	1	no
cDC1-LC	0	1	1	0	0	no
cDC1-Inflame DC	0	1	0	0	0	no
cDC1-Inflame TC	1	0	0	0	1	20538798；15813816；15113590；11876749；19667061
cDC2-Inflame FIB	1	0	1	0	1	no
cDC2-cDC1	1	1	1	0	1	no
cDC2-cDC2	1	1	1	1	1	no
cDC2-LC	0	1	1	1	1	no
cDC2-Inflame DC	0	1	1	0	1	no
cDC2-Inflame TC	1	0	0	0	1	20538798；15813816；15113590；11876749；19667061
cDC2-NKT	1	0	1	0	1	no
LC-Inflame FIB	1	0	1	1	1	no
LC-cDC1	0	1	0	0	0	no
LC-cDC2	0	1	1	1	0	no

LC-LC	0	1	1	1	0	no
LC-Inflame DC	0	1	0	0	0	no
LC-NKT	0	0	0	1	0	no
Inflame DC-Inflame FIB	1	0	1	0	1	no
Inflame DC-cDC2	0	1	1	0	1	no
Inflame DC-LC	0	1	1	0	0	no
Inflame DC-Inflame TC	1	0	0	0	0	20538798；15813816；15113590；11876749；19667061
Inflame TC-Inflame FIB	1	0	0	0	1	no
Inflame TC-cDC1	1	1	0	0	1	10808183
Inflame TC-cDC2	1	1	0	0	1	10808183
Inflame TC-LC	0	1	0	0	0	10808183
Inflame TC-Inflame DC	1	1	0	0	0	10808183
Inflame TC-Inflame TC	1	0	0	0	1	16815153；10808183；15813816
Inflame TC-NKT	1	0	0	0	0	no
NKT-Inflame FIB	1	0	0	0	1	no
NKT-cDC2	1	1	1	1	0	16978532
NKT-LC	0	1	1	1	0	16978532
NKT-Inflame DC	0	1	0	0	0	16978532
NKT-Inflame TC	1	0	0	0	0	no

Table S4. Literature curated of the inferred cellular communications between mouse brain cells

Cellular Communication	MDIC3	CellChat	Self-representation	PMID
astrocytes-astrocytes	0	1	0	21119776;19243701
astrocytes-ependymal	0	1	0	no
astrocytes-oligodendrocytes	1	1	1	21119776;12966207
astrocytes-ca1pyramidal	1	1	1	24204341
astrocytes-s1pyramidal	1	0	1	16212146
astrocytes-endothelial	0	1	1	19243701
astrocytes-mural	0	1	0	27775719
microglia-astrocytes	1	1	0	35201268;32765501
microglia-microglia	1	1	0	25171395
microglia-ependymal	0	1	0	no
microglia-oligodendrocytes	1	1	1	23981039
microglia-ca1pyramidal	1	0	1	30319362；23650361；31677479
microglia-s1pyramidal	1	0	0	30319362；23650361；31677479
microglia-interneurons	1	0	0	30319362；23650361；31677479
microglia-endothelial	1	0	0	35201268
microglia-mural	1	1	0	23416119
ependymal-astrocytes	0	1	0	21119776
ependymal-ependymal	0	1	0	no
ependymal-oligodendrocytes	0	1	0	21119776;12966207
oligodendrocytes-astrocytes	1	1	1	23717302
oligodendrocytes-microglia	1	0	1	23981039
oligodendrocytes-ependymal	0	1	0	no
oligodendrocytes-oligodendrocytes	1	0	1	33208471
oligodendrocytes-ca1pyramidal	1	0	1	31410988

oligodendrocytes-s1pyramidal	1	0	1	31410988
oligodendrocytes-interneurons	1	0	1	31410988
oligodendrocytes-endothelial	1	1	1	33969732
oligodendrocytes-mural	0	1	1	no
ca1pyramidal-astrocytes	1	1	1	27775719
ca1pyramidal-microglia	0	0	1	31729301
ca1pyramidal-ependymal	0	1	0	no
ca1pyramidal-oligodendrocytes	1	0	1	23717302
ca1pyramidal-ca1pyramidal	1	0	1	28069946；29547843
ca1pyramidal-s1pyramidal	1	0	1	no
ca1pyramidal-interneurons	1	0	1	no
ca1pyramidal-endothelial	1	0	1	31677479;14550787;28535372;25535736
ca1pyramidal-mural	0	1	1	no
s1pyramidal-astrocytes	1	1	1	27775719
s1pyramidal-ependymal	0	1	1	no
s1pyramidal-oligodendrocytes	1	0	0	23717302
s1pyramidal-ca1pyramidal	1	0	1	no
s1pyramidal-s1pyramidal	1	0	1	20369290
s1pyramidal-interneurons	1	0	1	20369290
s1pyramidal-endothelial	1	0	1	31677479;14550787;28535372;25535736
s1pyramidal-mural	0	1	1	no
s1pyramidal-microglia	0	0	1	31729301
interneurons-astrocytes	1	1	1	27775719
interneurons-microglia	1	0	0	31729301;25535736
interneurons-ependymal	1	0	0	no
interneurons-oligodendrocytes	1	0	1	23717302

interneurons-ca1pyramidal	1	1	1	no
interneurons-s1pyramidal	1	0	1	no
interneurons-interneurons	1	0	1	11777931
interneurons-endothelial	1	1	1	31677479;14550787;28535372;25535736
interneurons-mural	1	1	0	no
endothelial-astrocytes	1	1	1	15283992
endothelial-ependymal	0	1	0	no
endothelial-oligodendrocytes	1	0	1	31410988
endothelial-ca1pyramidal	1	1	1	no
endothelial-s1pyramidal	1	0	1	no
endothelial-interneurons	0	1	1	no
mural-astrocytes	0	1	0	30894190;23982198
mural-ependymal	0	1	0	no

Table S5. Results of MDIC3 and CellChat applied on mouse brain 10X visium spatial dataset

Cellular Communication	MDIC3	CellChat	label
Astro-Astro	1	1	1
Astro-L2/3 IT	0	0	1
Astro-L5 IT	1	0	0
Astro-L6 CT	1	0	0
Astro-L6 IT	1	0	1
Astro-L6b	0	0	0
Astro-Oligo	0	1	0
L2/3 IT-Astro	0	1	1
L2/3 IT-L2/3 IT	0	0	1
L2/3 IT-L5 IT	0	0	0
L2/3 IT-L6 CT	0	0	0
L2/3 IT-L6 IT	0	0	1
L2/3 IT-L6b	0	0	0
L2/3 IT-Oligo	0	1	0
L5 IT-Astro	1	1	0
L5 IT-L2/3 IT	0	0	0
L5 IT-L5 IT	1	0	1
L5 IT-L6 CT	1	0	1
L5 IT-L6 IT	1	1	1
L5 IT-L6b	1	0	0
L5 IT-Oligo	1	1	0
L6 CT-Astro	1	1	0
L6 CT-L2/3 IT	0	0	0
L6 CT-L5 IT	1	0	1

L6 CT-L6 CT	1	0	1
L6 CT-L6 IT	1	0	1
L6 CT-L6b	0	0	1
L6 CT-Oligo	0	1	0
L6 IT-Astro	1	1	1
L6 IT-L2/3 IT	0	0	1
L6 IT-L5 IT	1	0	1
L6 IT-L6 CT	1	0	1
L6 IT-L6 IT	1	0	1
L6 IT-L6b	1	0	1
L6 IT-Oligo	1	1	0
L6b-Astro	0	1	0
L6b-L2/3 IT	0	0	0
L6b-L5 IT	1	0	0
L6b-L6 CT	0	0	1
L6b-L6 IT	1	0	1
L6b-L6b	0	0	1
L6b-Oligo	0	1	1
Oligo-Astro	0	1	0
Oligo-L2/3 IT	0	0	0
Oligo-L5 IT	1	0	0
Oligo-L6 CT	0	0	0
Oligo-L6 IT	1	0	0
Oligo-L6b	0	0	1
Oligo-Oligo	0	1	1

Table S6. L-R pairs involved in the communication between inflame FIB and inflame DC by using MDIC3 and CellChat

	MDIC3	CellChat
Inflame FIB-Inflame DC	*CCL19-CCR7*<br>*CXCL12-CXCR4*	*CCL19-CCR7*<br>*CXCL12-CXCR4*<br>*MIF-(CD74+CXCR4)*

Table S7. L-R pairs involved in the communication between Basal-P and FIB-B by using MDIC3 and CellChat

	MDIC3	CellChat
(Basal-P)-(FIB-B)	*Pdgfa-Pdgfra*	*Pdgfa-Pdgfra*
	Pdgfc-Pdgfra	*Pdgfc-Pdgfra*
	Ngf-Ngfr	*Ngf-Ngfr*
	Pdgfa-Pdgfrb	*Pdgfa-Pdgfrb*
	Wnt10a-(Fzd2+Lrp5)	*Mdk-Sdc2*
	Wnt10a-(Fzd2+Lrp6)	*Mdk-Lrp1*
	Wnt16-(Fzd2+Lrp5)	*Ptn-Sdc2*
	Wnt16-(Fzd2+Lrp6)	*Ptn-Ncl*
	Wnt4-(Fzd2+Lrp5)	
	Wnt4-(Fzd2+Lrp6)	
	Wnt3-(Fzd2+Lrp5)	
	Wnt3 -(Fzd2+Lrp6)	
	Wnt6-(Fzd2+Lrp5)	
	Wnt6-(Fzd2+Lrp6)	
	Wnt7b-(Fzd2+Lrp5)	
	Wnt7b-(Fzd2+Lrp6)	
	Mpzl1-Mpzl1	

Table S8. L-R pairs involved in autocrine communication of Basal by using MDIC3 and CellChat

	MDIC3	CellChat
Basal-Basal	*Wnt10a-(Fzd6+Lrp5)*	*Pdgfa-Pdgfrb*
	Wnt10a-(Fzd6+Lrp6)	*Mdk-Sdc1*
	Wnt16-(Fzd6+Lrp5)	*Mdk-Sdc4*
	Wnt16-(Fzd6+Lrp6)	*Mdk-(ITGA6+ITGB1)*
	Wnt3-(Fzd10+Lrp5)	*Mdk-Ncl*
	Wnt3-(Fzd10+Lrp6)	*Ptn-Sdc1*
	Wnt4-(Fzd10+Lrp5)	*Ptn-Sdc2*
	Wnt4-(Fzd10+Lrp6)	*Ptn-Sdc4*
	Wnt7b-(Fzd10+Lrp5)	*Ptn-Nc1*
	Wnt7b-(Fzd10+Lrp6)	
	Wnt10a-(Fzd10+Lrp5)	
	Wnt10a-(Fzd10+Lrp6)	
	Wnt7b-(Fzd6+Lrp5)	
	Wnt7b-(Fzd6+Lrp6)	
	Wnt6-(Fzd10+Lrp6)	
	Wnt3-(Fzd6+Lrp5)	
	Wnt3-(Fzd6+Lrp6)	
	Wnt16-(Fzd10+Lrp5)	
	Wnt16-(Fzd10+Lrp6)	
	Wnt4-(Fzd6+Lrp5)	
	Wnt4-(Fzd6+Lrp6)	
	Wnt6-(Fzd6+Lrp5)	
	Wnt6-(Fzd6+Lrp6)	

Wnt6-(Fzd10+Lrp5)
Cdh1-Cdh1
Cadm1-Cadm1
Bmp7-(Bmpr1a+Acvr2a)
Bmp7-(Bmpr1a+Acvr2b)
Bmp7-(Bmpr1a+Bmpr2)
Agrn-Dag1
Lamb1-Dag1
Lamb1-(Itga6+Itgb4)
Col4a1-Sdc4
Col4a2-Sdc4

Table S9. Comparison of the feature of MDIC3 with other tools

		MDIC3	CellChat	Cellphonedb	iTALK
**Input**	Need for L-R database	N	Y	Y	Y
	Species restriction	any species	human and mouse	human	human
**Approach**	Basis of inference level	single cells	cell groups	cell groups	Cell groups
	Inference score	global	L-R or pathway	L-R	L-R
**Output**	Predict global communication	Y	N	N	N

Table S10. Literature support results under different gene coverage of datasets

	Literature support	No support
The original human islet dataset matrix (60.96%)	10	2
Matrix1 (70.01%)	10	3
Matrix2 (80.08%)	10	3
Matrix3 (91.25%)	9	6

Table S11. Computational cost of MDIC3 in simulation datasets

	Running time	Peak memory
gene number=20000, cell number=100	0.66min	7851.54 MiB
gene number=20000, cell number=500	1.02min	8066.65 MiB
gene number=20000, cell number=1000	1.20min	8334.51 MiB
gene number=20000, cell number=1500	1.74min	8602.74 MiB
gene number=20000, cell number=2000	2.04min	8871.08 MiB
gene number=20000, cell number=2500	2.46min	9139.76 MiB
gene number=20000, cell number=3000	2.70min	9408.53 MiB
gene number=20000, cell number=3500	3.06min	9676.65 MiB
gene number=20000, cell number=4000	4.15min	9943.76 MiB
gene number=20000, cell number=4500	4.42min	10210.82 MiB
gene number=20000, cell number=5000	4.95min	10477.86 MiB

Table S12. Computational cost of MDIC3 in real datasets

	Human lesional skin dataset	E14.5 mouse skin dataset	Mouse brain dataset
Data information	1394cells<br>11064genes	13179cells<br>14077genes	3005cells<br>19972gene
Running time	1.6min	16.067min	5.683min
Memory increments	9398.27 MiB	23059.16 MiB	23093.74 MiB

Table S13. Literature support results for communication results using different GRN tools

	Human lesional skin cell dataset		Human islet endocrine cell dataset	
	Literature support	No support	Literature support	No support
GNIPLR	14	13	10	2
GENIE3	13	13	10	2
GRNBoost	13	14	5	1

Supplemental Notes

Note S1. Discussion of the relationship between MDIC3 and GraphFP

First, similar to MDIC3, GraphFP[1] also does not use the L-R databases in its cell-cell interaction prediction process, and its cell-cell interaction prediction results are also overall results. The difference is that GraphFP is a nonlinear Fokker-Planck equation graph based model and dynamic inference framework, and considers the cell-cell interaction as a non-linear quadratic term. MDIC3 is a matrix decomposition model. An expression matrix of single cells contains regulatory information among genes and intercellular crosstalk information. Therefore, matrix decomposition can be used to uncover the regulatory or crosstalk patterns among cells from the expression matrix.

Secondly, GraphFP predicts interactions among different cell types and incorporates cell type information into the prediction process. In contrast, MDIC3 provides communication results among all individual cells. By integrating the results of communication among individual cells. MDIC3 can also obtain communication results among different cell types.

Finally, GraphFP considers cell state transitions as a dynamic process and aims to reconstruct cell state transitions from time-series single-cell transcriptome data, delineating cell-cell interactions that drive cell differentiation, focusing on the changes in cellular communication during the cell state transitions dynamic process. In contrast, MDIC3 primarily focuses on the analysis of cell-cell communication in a particular state. The article of GraphFP explores the changes in neuronal cell interactions at different time points during mouse development, which has parallels with our joint learning of skin development in E13.5 and E14.5 mouse. However, as MDIC3 does not rely on time-series single-cell transcriptome data, it has a wider range of applications compared to GraphFP. For example, the results of MDIC3 can be further used for the joint analysis of cell-cell communications across different disease states (such as the "Joint learning of lesional and nonlesional human skin" section). In addition, since GraphFP is an algorithm designed for predicting cell-cell interactions based on a large amount of dynamic time-series data, it is more suitable for the analysis of time-series data with multiple time points. It may prove more advantageous when exploring changes in cell-cell interactions at different time points.

Note S2. Exploring the effect of different gene coverage on prediction results

The proportion of zeros contained in the human islet dataset matrix we used was 60.96%. To investigate whether different gene coverage affects the prediction results of MDIC3, we randomly selected non-zero elements from the human islet dataset matrix and replaced them with zeros. This process resulted in the creation of three new matrices (Matrix1, Matrix2, Matrix3), containing 70.01% (Matrix1), 80.08% (Matrix2), and 91.25% (Matrix3) of the proportion of zeros, respectively. We re-inferred the cell-cell communications based on these three new matrices using MDIC3, and separately calculated the literature support rates from the results obtained from each matrix.

The literature support rate for the original human islet dataset matrix is 83.3% (10/12) and the literature support rate gradually decreases as the proportion of zeros in the matrix increases: 76.92% (10/13) for Matrix1, 76.92% (10/13) for Matrix2 and 60% (9/15) for Matrix3. These results indicate that the prediction effectiveness of MDIC3 can be affected by the drop-out effect or gene coverage of single cell data, but the fluctuation of the effect is acceptable. When the proportion of zeros in the single cell data reaches 90% or more, the literature support rates for the results predicted by MDIC3 remain over 50%, implying that the MDIC3 algorithm is stable and robust, and the results obtained from different gene coverage are also acceptable.

Note S3. Benchmark against ligand-receptor pairing accuracy

Considering that the CellChat, Cellphonedb, and iTALK algorithms are all L-R-based algorithms with their own L-R databases, we first derived significant L-R pairs obtained by applying each of the three algorithms to the human lesional skin dataset. Then, we obtained the average expression of all significant L-R pairs for each pair of cell type pairs, separately. Finally, we calculated the correlation between the expression results of each cell type pair under the three algorithms separately and the communication strength of each cell type pair obtained by MDIC3.

We applied this benchmarking approach to the human lesional skin dataset. The correlation of the L-R expression results between existing tools (CellChat, CellPhonedb, and iTALK) and MDIC3 with 49 cell types are (PCC: 0.335, p-value: 0.018), (0.414, p-value: 0.003), and (0.418, p-value: 0.002), respectively. This suggests that the results from MDIC3 are significantly correlated with the overall expression of L-R pairs.

Note S4. Benchmarking of how well the matrix decomposition actually work

Given a single-cell gene expression matrix A with size $m \times n$ as input, the matrix A contains m genes and n cells. MDIC3 inferred cell-cell communication through matrix decomposition, dividing the original single-cell gene expression matrix A into three matrices R, $\sum$, and W. The matrix R uses the adjacency matrix of the gene regulatory network (GRN) obtained by the publicly available algorithm GNIPLR[2], which calculates a unique GRN adjacency matrix. The matrix $\sum$ is the complete singular value matrix of matrix A. The matrix W is the cell-cell communication adjacency matrix and is the only result obtained using the Moore-Penrose pseudo-inverse. We consider that these three matrices R, $\sum$, and W are able to reconstruct the original single-cell gene expression matrix A. We calculated the Mean Squared Error (MSE) between the reconstructed matrix $R\sum W$ and the original single-cell gene expression matrix A using the human lupus nephritis dataset, the E14.5 mouse skin dataset, and the mouse brain dataset respectively. The MSE is calculated as follows:

$$MSE = \frac{1}{m \times n}\sum_{i=1}^{m \times n}(A_i - (R\sum W)_i)^2$$

The human lupus nephritis dataset contains 2838 cells and 22447 genes. The E14.5 mouse skin

dataset contains 12179 cells and 14077 genes. The mouse brain dataset contains 3005 cells and 19972 genes. The MSEs between the reconstructed matrix $R\sum W$ and the original single-cell gene expression matrix A under the human lupus nephritis dataset, the E14.5 mouse skin dataset, and the mouse brain dataset are 0.22, 0.25, and 0.24 respectively.

Note S5. The computational cost of MDIC3

We have tested the MDIC3 algorithm in terms of running time and running memory by simulating single-cell expression matrices with different sizes. The simulation steps are as follows:

(1) Randomly generate a matrix A of a given size, with the number of rows m denoting the number of genes simulated and the number of columns n denoting the number of cells simulated.

(2) Randomly generate a matrix R of size $m \times m$ as the simulated GRN adjacency matrix.

(3) Calculate the complete singular value matrix $\sum$ of A, multiplying R and $\sum$.

(4) Calculate the $(R\sum)^{+}A$ as the simulated cell-cell communication adjacency matrix W.

We tested the computational costs of MDIC3 with different simulation matrices containing 20,000 genes and 100 to 5,000 cells and recorded the total running time and peak memory usage for the simulation. The results are shown in Table S8.

We found that when the number of genes reached 20,000 and the number of cells reached 5,000, the computation time for MDIC3 was 4.96 minutes, and the memory consumption was 10.4GB. As the matrix size increases, the memory required for MDIC3 calculations also increases.

We also recorded the running time and memory increments for the human lesional skin cell dataset, E14.5 mouse skin dataset, and mouse brain dataset in Table S9. The E14.5 mouse skin dataset cost the longest running time (about 16 min), and both the E14.5 mouse skin dataset and the mouse brain dataset cost similar memory consumption (about 23GB). It should be noted that the full running cost of MDIC3 is focused on the calculation of GRN using the GNIPLR algorithm. Considering that any tool that infers regulatory networks can be used for MDIC3, and MDIC3 is not limited to GNIPLR, here, we only recorded the running time and memory increments for MDIC3 to solve for cellular communication after the GRN was calculated.

We also recorded the running time and memory increments during the run of the CellChat algorithm on the three datasets. The running time of CellChat on the human lesional skin cell dataset is 1.367 min, and the memory increments are 16.5kb. The running time of CellChat on the E14.5 mouse skin dataset is 7.316 min, and the memory increments are 16kb. The running time of CellChat on the mouse brain dataset is 2.66 min, and the memory increments are 29.2 MiB. After comparison, we found that MDIC3 takes longer to run, requires more running memory, and costs more to run. However, in general, we consider these computational costs acceptable for a personal computer.

Note S6. Details for calculating gene regulatory network (GRN)

Accurate and fast calculation of GRN is critical for MDIC3. Supposing the single-cell gene

expression matrix is $A_{m\times n}$ with *m* cells and *n* genes, and the adjacency matrix of GRN is $R_{m\times m}$ from single-cell gene expression. We made some optimizations to the calculation process of the adjacency matrix $R_{m\times m}$.

Step 1: Grouping genes.

The m genes in the single-cell gene expression data can be represented as $g_1, g_2, \cdots, g_m$. We divided m genes into l groups in order from g_1 to g_m, each group contains u genes, $u < m$. To be more specific, the u genes contained in the first gene group G_1 are g_1, g_2, $\cdots$, g_u, the u genes contained in the second gene group G_2 are g_{u+1}, g_{u+2}, $\cdots$, g_{2u}, so on, and genes contained in the l-th gene group G_l are $g_{(l-1)\times u+1}$, $g_{(l-1)\times u+2}$, $\cdots$, g_m.

Step 2: Compute the adjacency matrix through submatrices.

We divide the matrix $R_{m\times m}$ into l^2 blocks and R can be expressed as:

$$R = \begin{pmatrix} R_{11} & R_{12} & \cdots & R_{1l} \\ R_{21} & R_{22} & \cdots & R_{2l} \\ \vdots & \vdots & \cdots & \vdots \\ R_{l1} & R_{l2} & \cdots & R_{ll} \end{pmatrix}$$

Each block R_{ij} is a GRN adjacency submatrix with size $u\times u$ and represents the regulation from the u genes contained in the i-th gene group G_i to the u genes contained in the j-th gene group G_j. So, we convert the problem of calculating the adjacency matrix $R_{m\times m}$ into calculating l^2 adjacency submatrices.

All adjacency submatrix calculations in this paper use the GNIPLR[2] algorithm, where the regularization parameter $\alpha = 0.1$.

Step 3: Integrate to get the complete adjacency matrix.

Integrate all the l^2 submatrices and obtain the complete GRN adjacency matrix $R_{m\times m}$.

Note S7. Details of the aggregated communications

The different tools use different strategies to present their inference communication strength. We used the communication probability inferred by CellChat, the L-R average gene expression inferred by CellPhoneDB, and the L-R average gene expression inferred by iTALK as the inferred communication strength of these three tools. These measures are the default communication strength for the three existing algorithms. The results obtained from each tool were independent, and we did not normalize the communication strengths across the different tools. Furthermore, since none of the three tools mentioned thresholds for inferring results in their articles or program packages, we used the mean value of communication strengths as a unifying criterion. In other words, we calculated the mean value of communication intensity as the threshold for each tool, and our results follow the same way of calculating thresholds.

Details of obtaining the aggregated communication results from CellChat.

Use R package CellChat v1.1.3[3] to infer intercellular communications in scRNA-seq datasets.

The calculation process for each dataset uses default parameters.

The R package of CellChat can calculate the aggregated cell-cell communication results by summarizing the communication probability under each pathway or L-R pair between any two cell types. The aggregated communication results can be directly derived by the R package of CellChat.

Details of obtaining the aggregated communication results from Cellphonedb.

Use Python package Cellphonedb v2.0.0[4] to infer intercellular communications in scRNA-seq datasets.

Use the default command "cellphonedb method statistical_analysis meta.txt counts.txt [--counts-data=gene_name] --iterations=100 --threads=2"

Cellphonedb derives the means of the averaged ligand expression of cells in the ligand cell type and the averaged receptor expression of cells in the receptor cell type under a specific ligand-receptor (L-R) pair and considers the derived result as the communication strength between the two cell types under the specific L-R pair. As the same with CellChat, we summed the communication strengths under each L-R pair as the aggregated communication strength between any two cell types.

Details of obtaining the aggregated communication results from iTALK.

Use R package iTALK v0.1.0[5] to infer intercellular communications in scRNA-seq datasets.

The calculation process for each dataset uses default parameters.

iTALK calculates the averaged ligand expression of cells in the ligand cell type and the averaged receptor expression of cells in the receptor cell type under the specific L-R pair. Like Cellphonedb, we first averaged the mean expression values of ligand and receptor and consider this averaged result as the communication strength between two cell types under the specific L-R pair. Then, as the same with CellChat, the aggregated communication strength between any two cell types will be calculated by summing the communication strength under each L-R pair.

Note S8. Details of the two-side self-representation model

The two-side self-representation model is a matrix decomposition model. Given a single-cell gene expression matrix A with size $m \times n$ as input, the matrix A contains m genes and n cells. The one-side self-representation model of matrix A can be represented as

$$A = ZA \tag{S1}$$

$$A = AX \tag{S2}$$

Where matrix Z with size $m \times m$ is the similarity between genes and matrix X with size $n \times n$ is the similarity between cells.

Combined the equation (S1) and (S2), we can obtain the two-side self-representation model to factorize the single-cell gene expression matrix A:

$$A = ZAX \tag{S3}$$

Then the matrix A can be represented by itself and two other matrices Z and X. The matrix Z

is the left sub-matrix with the size of $m \times m$ and presents the regulatory relationship among genes, the m denotes the number of genes in single-cell expression matrix A; The matrix X is the right sub-matrix with the size of $n \times n$ and can be used to present the relationship among cells, the n denotes the number of cells in single-cell expression matrix A.

If we attempt to obtain the communication relationship among cells, we should figure out X from the equation (S3). The matrix Z can represent the regulatory relationship among genes, so we can use adjacent matrix of matrix A to replace the matrix Z, then matrix X can be solved from equation (S3). In Eqn. (S3), the two-side self-representation model can use the original single-cell expression matrix itself as the mediation matrix to infer the cell-cell communication network. So, the two-side self-representation model can obtain the cell-cell communication network by solving the matrix X:

$$X = (ZA)^{+} A \qquad \text{(S4)}$$

Where $(ZA)^{+}$ represent the pseudoinverse matrix of (ZA) and can be computed by the Moore-Penrose pseudo-inverse[6] of matrix (ZA). The singular matrix of matrix A can be used to replace the matrix A in two-side self-representation model. Then, we can obtain a new matrix decomposition pattern for matrix A:

$$A = R \sum W \qquad \text{(S5)}$$

Where the matrix R is the left sub-matrix with the size of $m \times m$ and presents the regulatory relationship among genes, the m denotes the number of genes in single-cell expression matrix A; The matrix W is the right sub-matrix with the size of $n \times n$ and can be used to present the relationship among cells, the n denotes the number of cells in single-cell expression matrix A; The matrix $\sum$ is the singular matrix of matrix A with dimension $m \times n$, which represents the mediation matrix connected the relationships between genes and cells.

Considering that both equation (S3) and (S5) can infer intercellular relationships based on intergenic relationships from single-cell expression profiles, we compared the results of (S3) and (S5) using different datasets. The literature support ratio in the results inferred by the (S5) under each dataset is always higher than the (S3) model (Figure 5A, Figure S5A and S5C). By comparing the final solution results of the two methods, the better performance of equation (S5) may be attributed to the fact that it uses the singular matrix as the mediation matrix, and the singular matrix can better extract the information containing the relationships between genes and cells, while the (S3) model uses the original single-cell expression matrix as the mediation matrix, which may contain additional noises.

Supplemental Reference

S1. Jiang, Q., Zhang, S., and Wan, L. (2022). Dynamic inference of cell developmental complex energy landscape from time series single-cell transcriptomic data. PLoS Comput. Biol. *18*, e1009821. 10.1371/journal.pcbi.1009821.

S2. Zhang, Y.L., Chang, X., and Liu, X.P. (2021). Inference of gene regulatory networks using pseudo-time series data. Bioinformatics *37*, 2423-2431. 10.1093/bioinformatics/btab099.

S3. Jin, S.Q., Guerrero-Juarez, C.F., Zhang, L.H., Chang, I., Ramos, R., Kuan, C.H., Myung, P., Plikus, M.V., and Nie, Q. (2021). Inference and analysis of cell-cell communication using CellChat. Nat. Commun. *12*, 20, 1088. 10.1038/s41467-021-21246-9.

S4. Vento-Tormo, R., Efremova, M., Botting, R.A., Turco, M.Y., Vento-Termo, M., Meyer, K.B., Park, J.E., Stephenson, E., Polanski, K., Goncalves, A., et al. (2018). Single-cell reconstruction of the early maternal-fetal interface in humans. Nature *563*, 347-+. 10.1038/s41586-018-0698-6.

S5. Yuanxin, W., Ruiping, W., Shaojun, Z., Shumei, S., Changying, J., Guangchun, H., Michael, W., Jaffer, A., Andy, F., and Linghua, W. (2019). iTALK: an R Package to Characterize and Illustrate Intercellular Communication. bioRxiv, 507871. 10.1101/507871.

S6. Strang, G. (1980). 3 - WITHDRAWN: ORTHOGONAL PROJECTIONS AND LEAST SQUARES. In Linear Algebra and its Applications (Second Edition), G. Strang, ed. (Academic Press), pp. 103-152. https://doi.org/10.1016/B978-0-12-673660-1.50006-X.
